# One Dianionic Luminophore with Three Coordination Modes Binding Four Different Metals: Toward Unexpectedly Phosphorescent Transition Metal Complexes

**DOI:** 10.1002/advs.202306801

**Published:** 2023-12-31

**Authors:** Thomas M. Kirse, Iván Maisuls, Leander Spierling, Alexander Hepp, Jutta Kösters, Cristian A. Strassert

**Affiliations:** ^1^ Institut für Anorganische und Analytische Chemie Universität Münster Corrensstr. 28/30 48149 Münster Germany; ^2^ CiMiC SoN and CeNTech Universität Münster Heisenbergstr. 11 48149 Münster Germany

**Keywords:** phosphorescence, photophysics, spectroscopy, synthesis, transition metal complexes

## Abstract

This work reports on a battery of coordination compounds featuring a versatile dianionic luminophore adopting three different coordination modes (mono, bi, and tridentate) while chelating Pd(II), Pt(II), Au(III), and Hg(II) centers. An in‐depth structural characterization of the ligand precursor (**H_2_L**) and six transition metal complexes (**[HLPdCN*t*Bu]**, **[LPtCl]**, **[LPtCN*t*Bu]**, **[LPtCNPhen]**, **[HLHgCl]**, and **[LAuCl]**) is presented. The influence of the cations and coordination modes of the luminophore and co‐ligands on the photophysical properties (including photoluminescence quantum yields (*Φ*_L_), excited state lifetimes (*τ*), and average (non‐)radiative rate constants) are evaluated at various temperatures in different phases. Five complexes show interesting photophysical properties at room temperature (RT) in solution. Embedment in frozen glassy matrices at 77 K significantly boosts their luminescence by suppressing radiationless deactivation paths. Thus, the Pt(II)‐based compounds provide the highest efficiencies, with slight variations upon exchange of the ancillary ligand. In the case of **[HLPdCNtBu]_,_** both *Φ*_L_ and *τ* increase over 30‐fold as compared to RT. Furthermore, the Hg(II) complex achieves, for the first time in its class, a *Φ*_L_ exceeding 60% and millisecond‐range lifetimes. This demonstrates that a judicious ligand design can pave the way toward versatile coordination compounds with tunable excited state properties.

## Graphical abstract

The synthesis of a dianionic luminophore with three coordination modes able to bind four different metal cations (Pd(II), Pt(II), Au(III), and Hg(II), i.e., with *d* ^8^ or *d* ^10^ electronic configurations) is presented, as well as its unexpectedly phosphorescent transition metal complexes.

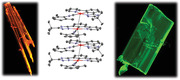

## 1 Introduction

In recent decades, late transition metal complexes have garnered substantial interest due to their potential applications in a variety of fields, such as optoelectronics,^[^ ^1^, ^2^, ^3^, ^4^, ^5^, ^6^, ^7^ ^]^ (photo‐)catalysis,^[^ ^8^, ^9^, ^10^ ^]^ solar energy conversion,^[^ ^11^, ^12^, ^13^ ^]^ as photoluminescent dyes,^[^ ^14^, ^15^, ^16^, ^17^, ^18^, ^19^, ^20^, ^21^ ^]^ and in metal‐based medicine,^[^ ^22^, ^23^, ^24^, ^25^, ^26^, ^27^, ^28^ ^]^ among others. Particularly, coordination compounds featuring second and third‐row transition metal centers, such as Pt(II), Pd(II), Au(III), Ru(II), and Re(I), have emerged as promising candidates for the development of photofunctional materials, owing to their physicochemical characteristics, including thermal and photochemical stability, excited‐state properties, and large Stokes‐shifts paired with long excited state lifetimes, among others.^[^ ^18^, ^19^, ^29^, ^30^, ^31^, ^32^, ^33^, ^34^, ^35^, ^36^, ^37^, ^38^, ^39^, ^40^, ^41^, ^42^, ^43^ ^]^ As stated in the bibliographic literature for a vast number of metals, the photophysical and photochemical properties of transition metal complexes can be finely tuned by judiciously combining the chromophoric ligand (whether bi, tri, or tetradentate) with a specific monodentate ancillary co‐ligand possessing different degrees of donor and acceptor character.^[^ ^4^, ^34^, ^35^, ^36^, ^44^, ^45^, ^46^, ^47^, ^48^, ^49^ ^]^ Various designs of chromophores with a variety of coordinating atoms, as well as different binding patterns, have been established for different transition metal complexes. Pyridine has been commonly employed as a directing group in C‐H functionalization reactions, owing to its notable ability to bind to transition metals, which facilitates the C‐H activation step.^[^ ^50^, ^51^, ^52^, ^53^, ^54^, ^55^, ^56^ ^]^ Furthermore, cyclometallation has emerged as an appealing strategy to modulate the ligand field strength in luminescent coordination compounds by the formation of a covalent carbon‐to‐metal bond. This results in a significant increase of the *d*‐orbital splitting through the strong sigma‐donating character of the C_aryl_‐species.^[^ ^38^, ^57^ ^]^ Therefore, cyclometallating bidentate chelators featuring pyridine‐based N‐donors (i.e., C^N), in conjunction with three different classes of tridentate ligands incorporating N^C^N, C^N^N, and C^N^C motifs, have been investigated with metal ions including Ni(II), Pd(II), Pt(II), Ir(III), among others.^[^ ^36^, ^37^, ^38^, ^58^, ^59^, ^60^, ^61^, ^62^, ^63^, ^64^, ^65^, ^66^, ^67^, ^68^, ^69^, ^70^, ^71^, ^72^, ^73^, ^74^ ^]^ While abundant literature is found regarding symmetric tridentate ligands based on the N^C^N pattern established by Williams and co‐workers,^[^ ^75^ ^]^ asymmetric pincer chelators have received limited attention in comparison to their counterparts, mainly due to the relatively intricate synthetic procedures required.^[^ ^62^, ^65^, ^66^, ^67^, ^68^, ^69^, ^70^, ^76^ ^]^ In the case of the asymmetric ligands, while most of the features regarding the coordination‐chemical structure remain practically constant, the replacement of a pyridyl unit by a smaller *π*‐system (like 1*H*‐1,2,4‐triazole) alters the nature of the ligand‐centered orbitals; thus the photophysical properties of the complexes can be modified.

Moreover, the dianionic nature of the ligand precursor, in conjunction with group 10 dications, such as Ni(II), Pd(II), and Pt(II), paves the road for novel coordination compounds using neutral monodentate *σ*‐donors with varying degrees of *π*‐accepting character, including co‐ligands based on isonitriles, pyridine‐derivatives, or phosphanes.^[^ ^4^, ^35^, ^36^, ^44^, ^45^, ^47^, ^77^, ^78^, ^79^ ^]^ This approach also offers an easier way of modulating both the structural and photophysical properties of the resulting complexes, not only by variation of the bulkiness and degree of co‐planarization, but also by atomic number of the chelated atom.

Due to the electron configuration of *d* ^8^‐metal ions, the derived complexes tend to have a square planar coordination geometry; thus, metal–metal interactions with electronic coupling can be attained. Pt(II)‐based complexes are known to have a clear tendency toward aggregation, depending both on their spatial configuration and microenvironment (e.g., sterical hindrance and bulkiness of the ligand, among others), where the Pt–Pt distances can reach values below 3.5 Å.^[^ ^35^, ^36^, ^45^, ^80^, ^81^, ^82^, ^83^ ^]^ At this short distance, the intermolecular coupling of the $d_{z^{2}}$ orbitals with their lobes protruding out of the coordination plane are favored and hence excimers or aggregates can be formed. In general, the emerging red‐shifted luminescence of aggregated species in Pt(II)‐based compounds stems from excited states with metal–metal‐to‐ligand charge‐transfer character (MMLCT, $d_{z^{2}}$‐$d_{z^{2}}$‐π*‐π*), whereas the emission of the monomers occurs mostly from metal‐perturbed ligand‐centered triplet states (^3^MP‐LC) as admixtures of ligand‐centered (LC, *π*‐π*) and metal‐to‐ligand charge‐transfer (MLCT, *d*‐π*) configurations.^[^ ^3^, ^4^, ^16^, ^17^, ^59^, ^84^, ^85^, ^86^, ^87^ ^]^ In contrast, while Pd(II) based compounds are mostly known for their catalytic activity, there is only a scarce number of reports regarding luminescent complexes.^[^ ^60^, ^81^, ^88^ ^]^ Due to their significantly lower ligand‐field splitting, dissociative metal‐centered (MC, π/d‐d*) states of Pd(II) complexes are thermally accessible, favoring a nonradiative deactivation by excited state distortion and conical intersections with the ground state.^[^ ^81^, ^87^, ^88^, ^89^ ^]^ Additionally, *d* ^10^‐configured metal chelates are promising candidates for the production of photoactive materials particularly in terms of photo‐/electro‐luminescence, owing to their relatively low cost and thermal stability.^[^ ^90^, ^91^ ^]^ In addition to linear *d* ^10^‐configured coordination compounds and despite the widespread interest in square planar *d^8^*‐based complexes with tridentate mono‐/di‐anionic luminophores, comparable studies regarding the isoelectronic Au(III) ions are less abundant.^[^ ^39^, ^40^, ^41^, ^42^, ^43^, ^92^, ^93^ ^]^ Even though Au(III) holds significant promise as a cost‐effective alternative to Pt/Pd in the development of innovative triplet emitters, its utilization still remains less explored, mainly due to the lack of adequate ligands.^[^ ^39^, ^40^, ^41^, ^42^, ^43^ ^]^

In this work, a new cyclometallating dianionic N^C^N‐type ligand offering three different coordination modes (mono‐/bi‐/tridentate) and enabling the complexation of four different transition metal ions, including Pd(II), Pt(II), Au(III), and Hg(II), is presented. Thus, six new complexes were synthesized and characterized. In contrast to the already well‐established synthesis of monoanionic N^C^N‐type ligands,^[^ ^38^, ^65^, ^68^, ^76^, ^94^, ^95^ ^]^ neutral complexes of M(II)‐ and M(III)‐ions (where M is a transition metal) can be obtained in combination with neutral or monoanionic monodentate co‐ligands, respectively, due to their intrinsic dianionic character. Hence, the monodentate co‐ligands were carefully chosen in order to modulate the excited electronic states of the complexes, as well as the photophysical and structural properties. All compounds (ligand precursors and complexes) were characterized by means of two‐dimensional nuclear magnetic resonance spectroscopy (2D‐NMR, ^1^H, ^13^C, ^15^N, and ^195^Pt) and exact mass spectrometry (EM‐MS). In addition, the crystal structure of four complexes was elucidated by means of X‐ray diffractometric (XRD) analysis investigating their molecular structure, as well as molecular packing in the crystal. In addition, a complete study of the photophysical properties is presented in liquid solutions at room temperature (RT, both air‐equilibrated and Ar‐purged) as well as in frozen glassy matrices at 77 K by time‐resolved and steady‐state photoluminescence spectroscopy. Also, the crystalline samples were analyzed by means of time‐resolved photoluminescence micro(spectro)scopy using 1‐photon and 2‐photon excitation sources (SPE and TPE, respectively).

In summary, a new dianionic N^C^N‐type ligand capable of chelating different transition metal cations is presented, where depending on the transition metal and the monodentate ancillary ligand, different coordination modes and photophysical properties (such as the emission maxima, lifetimes, and quantum yields) become accessible.

## 2 Results and Discussion

### 2.1 Synthesis and Characterization

**Scheme 1:**
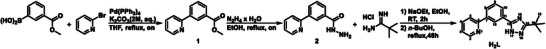
General synthetic procedure to obtain the herein reported ligand precursor **H**_**2**_ **L**. Further information regarding synthesis, purification, and characterization are detailed in the Supporting Information. ^*^on: Overnight.

Due to the poor reactivity of benzonitrile and its derivatives when simply using hazardous hydrazine, an alternative synthetic pathway was explored for the synthesis of a dianionic cyclometallating, triazol‐containing ligand precursor with an N^C^N‐type coordination motif. The reactions were carried out by adapting and optimizing methodologies from earlier studies on comparable syntheses.^[^ ^35^, ^96^, ^97^ ^]^ First, 3‐(methoxycarbonyl)phenylboronic acid was reacted with 2‐bromopyridine in a Suzuki‐Miyaura cross‐coupling reaction to obtain methyl 3‐(pyridin‐2‐yl)benzoate (**1**) in good yields (80%). In a second step, **1** was reacted with a tenfold excess of hydrazine hydrate in order to obtain the corresponding 3‐(pyridin‐2‐yl)benzohydrazide (**2**) in quantitative amounts (100%). The reaction of pivalamidine hydrochloride in ethanolic solution with sodium ethoxide led to pivalamidine (free base), which further reacted with **2** in a cyclization reaction under elimination of water to provide the ligand precursor **H_2_L** in good yields (65%), as detailed in **Scheme** **1**.

**Scheme 2:**
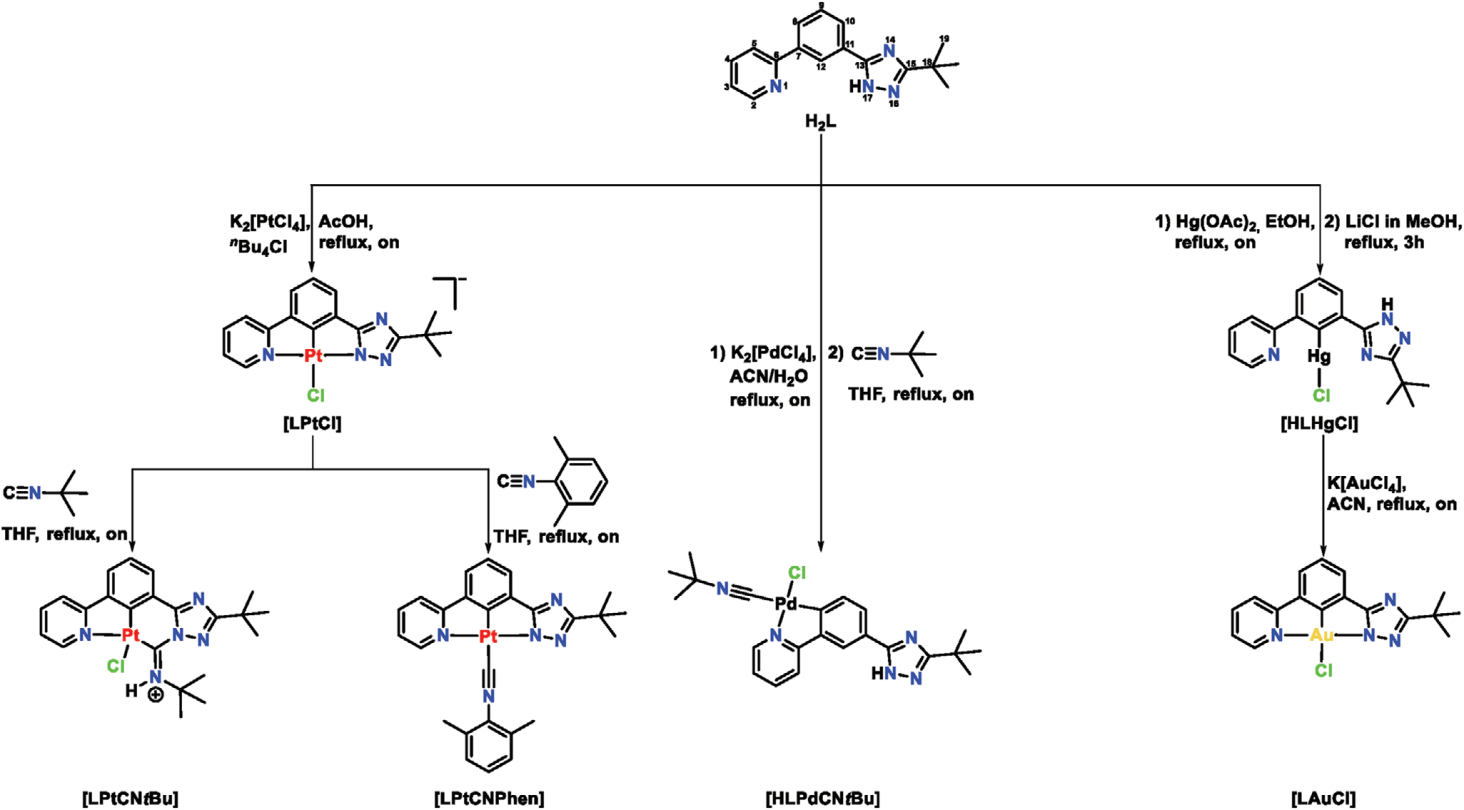
General synthetic procedure to obtain the herein reported complexes with the dianionic N^C^N ligand. Further information regarding synthesis, purification, and characterization are detailed in the Supporting Information. ^*^on: Overnight.

The coordination of **H_2_L** with various metal precursors (Pt^II^, Pd^II^, Au^III^, and Hg^II^) in conjunction with diverse ancillary ligands resulted in six novel coordination compounds. With regards to the Pt(II) complexes, cyclometalation of K_2_[PtCl_4_] in glacial acetic acid led to **[LPtCl]** in good yields (60%). Further reaction of **[LPtCl]** in tetrahydrofuran (THF) under reflux conditions with two different isonitriles (*tert*‐butylisonitrile and 2,6‐dimethylphenyl isonitrile) gave two different coordination motifs for the corresponding isonitrile moiety, and the complexes **[LPtCN*t*Bu]** as well as **[LPtCNPhen]** were obtained (**Scheme** **2**). Further endeavors involving ligand exchange procedures utilizing **[LPtCl]** and various neutral monodentate co‐ligands (including pyridines and phosphanes) were explored. However, these attempts were unsuccessful in yielding the desired complexes. Attempts toward comparable Pd(II) compounds were carried out in the same manner using different Pd(II) precursors (Li_2_[PdCl_4_], Na_2_[PdCl_4_] and K_2_[PdCl_4_]), as already published by Soro et al. for comparable N^C^N‐coordinated compounds.^[^ ^95^ ^]^ Alternative Pd(II)‐sources, including PdCl_2_ or PdOAc, as well as different solvent combinations, were evaluated but failed to yield the target compound. In particular, a stoichiometric amount of **H_2_L** was reacted with K_2_[PdCl_4_] in a boiling mixture of acetonitrile (ACN) and water (H_2_O) overnight (on), followed by a direct ligand exchange with *tert*‐butylisonitrile (*t*Bu‐NC) in THF under reflux (Scheme 2). Interestingly, instead of the expected cyclometalation at the C12 position (required for a tridentate coordination pattern), a rotation of the pyridine unit was observed, leading to a cyclometalation at the other adjacent carbon (C8) with respect to the pyridine moiety and, consequently, to a bidentate coordination around the Pd(II)‐center (**[HLPdCN*t*Bu]**). This interesting outcome can be explained considering a C‐H activation directed by the initial coordination of the pyridine unit to the Pd(II)‐center. Furthermore, the lack of *ortho* CH‐activation could be related to the absence of Cl‐dissociation deriving from the corresponding Pd(II)‐precursor, due to the harder nature of Pd(II) (as compared to Pt(II) with concomitantly increased bulk of the [PdCl_4_]^−^ species). Interestingly, no other coordination motif was obtained by changing the Pd(II) precursor. Based on the direct comparison of Pt(II) and Pd(II), it can be assumed that pre‐coordination of both N‐donors, namely N‐pyridine and N‐triazole, is necessary to facilitate the C‐H activation of *d^8^*‐configured metal ions at the C12 position, which corresponds to a highly hindered intermediate, as previously reported.^[^ ^62^ ^]^ Nonetheless, further efforts toward a C12 cyclometalated Pd(II)‐complex via a transmetalation reaction involving a Hg(II) intermediate were made. A slightly modified literature procedure^[^ ^95^ ^]^ was followed in order to attain the Hg(II) complex; briefly, a stoichiometric amount of mercury(II) acetate (Hg(OAc)_2_) and **H_2_L** were refluxed overnight in ethanol (EtOH), followed by addition of a LiCl solution in methanol (MeOH) to yield **[HLHgCl]** (70%). Subsequently, various reaction conditions were systematically examined in an attempt to synthesize the C12 cyclometalated Pd(II)‐complex through transmetalation. However, these efforts failed to yield the desired complex. Further details about the synthesis and purification can be found in the Supporting Information.

Finally, a slightly modified procedure was followed in order to obtain an **H_2_L**‐based Au(III) complex. Hence, a single reaction path was followed, where **[HLHgCl]** was mixed with K[AuCl_4_] in ACN under reflux to yield **[LAuCl]**. Further attempts toward the co‐ligand exchange involving **[LAuCl]** and different neutral or monoanionic monodentate species were tested (cyanido, 3,6‐di‐*tert*‐butylcarbazolato, *tert*‐butylisonitrile and 2,6‐dimethylphenyl isonitrile), yet without the expected success.

The structures of all compounds were assessed by 2D‐NMR spectroscopy and EM‐MS (see Figures S1–S65, Supporting Information) where all the signals were unambiguously assigned. Single crystals suitable for X‐ray diffractometric analysis were obtained by slow diffusion of *n*‐hexane into saturated dichloromethane (DCM)/MeOH or ethyl acetate (EtOAc) solutions. A compilation of the detailed experimental procedures and analytical data is provided in the Supporting Information.

### 2.2 Structural Characterization

**Figure 1:**
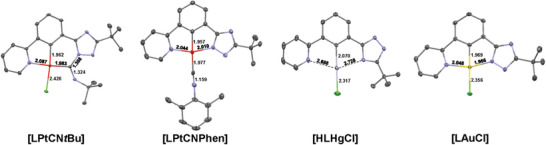
Molecular structure in single crystals and ORTEP diagrams of **[LPtCNtBu]**, **[LPtCNPhen]**, **[HLHgCl]**, and **[LAuCl]** (displacement ellipsoids are shown at 50% probability). The average bond lengths (*d*) are also detailed. Hydrogen atoms are omitted for clarity. For labelled molecules, see Figures S66–S70, Supporting Information.

The molecular structures in single crystals were determined by XRD analysis for **[LPtCNtBu]**, **[LPtCNPhen]**, **[HLHgCl]**, and **[LAuCl]**, as shown in **Figure** **1**. Attempts to obtain suitable single crystals of **[HLPdCN*t*Bu]** were not successful. Selected data and appropriate refinement parameters can be found in the next section, as well as in the Supporting Information. Further details regarding molecular structures in single crystals as Oak Ridge themal‐ellipsoid plot (ORTEP) diagrams as well as the molecular packing for each compound can be found in the Supporting Information.

As mentioned before, cyclometalation at the C12 position was observed in all cases. However, coordination via the N^C^N‐mode was demonstrated for **[LPtCNPhen]** and **[LAuCl]**, whereas a linear complex was found for **[HLHgCl]**. When comparing **[LPtCNPhen]** and **[LAuCl]**, similar bond distances between the metal center and the tridentate chelator were observed. In the case of Au(III), the M‐N_triazole_ distance is shorter, which can be related to the higher charge density at the Au(III) center, particularly if compared with Pt(II), leading to a stronger electrostatic interaction and therefore a shorter bond distance. Due to the linear coordination of Hg(II) in **[HLHgCl]**, the M‐N_triazole_/N_pyridine_ distances are remarkably longer (compared to covalent Hg(II)‐N bonds), together with a slight rotation of the triazole and pyridine units (9.8° and 14.9°, respectively) out of the C‐M‐Cl coordination plane (including a 180° twisted triazole), contrasting with those observed in tridentate‐coordinated complexes (see Figures S68 and S69, Supporting Information).

**Scheme 3:**
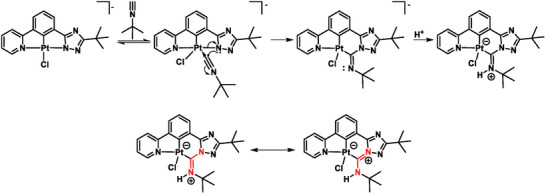
Proposed mechanism toward the obtained **[LPtCNtBu]** structure (top). Resonance structures of the open‐chained bis(imino)carbene‐like species (bottom).

Regarding both Pt(II) complexes, substantial differences were observed for **[LPtCNPhen]**, as a distorted square‐planar coordination around the Pt(II)‐center was observed with binding angles ranging from 79° (N_pyridine_‐Pt‐C_phenyl_) up to 103° (C_CNPhen_‐Pt‐N_pyridine_). The CN‐bond length (1.159 Å) corresponds to a triple bond, while the ancillary ligand is completely coplanar with the tridentate ligand. However, in the case of **[LPtCNtBu]**, a surprisingly different structure was observed. As shown in Figure 1 and Figure S66, Supporting Information, the carbon atom of the isonitrile is inserted into the Pt‐N_triazole_ bond, leading to a six‐ (Pt‐phenyl‐triazole‐CN*t*Bu) instead of a five‐ (Pt‐phenyl‐triazole) membered chelate‐metal ring, together with the formation of an open‐chained bis(imino)carbene‐like structure (**Scheme** **3**), in agreement with recent observations on a related compound.^[^ ^98^ ^]^

These findings can be explained by an intramolecular reaction between the negatively charged nitrogen atom of the deprotonated triazole ring (N_triazole_) and the isonitrile, where the carbon atom of the isonitrile (CN) is attacked by the free electron pair of the N_triazole_ while leading to the insertion of the C_CN_ *_t_*_Bu_ into the Pt‐N_triazole_‐bond. As a consequence, an elongation of the C‐N bond that can be correlated with a decrease in bond order going from a triple (1.166–1.184 Å) to a double bond (1.324 Å), is revealed.^[^ ^99^, ^100^, ^101^ ^]^ In this case, a slightly distorted square‐planar coordination around the Pt(II)‐metal center was observed, where the X‐Pt‐Y bond angle spans from 81° (C_phenyl_‐Pt‐N_pyridine_) up to 94° (C_CN_ *_t_*_Bu_‐Pt‐C_phenyl_).

**Figure 2:**
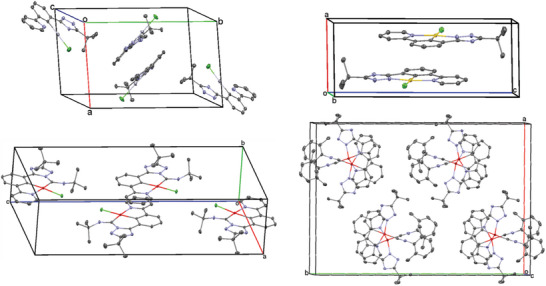
Packing diagram in the unit cell of **[HLHgCl]** (top left), **[LAuCl]** (top right), **[LPtCNtBu]** (bottom left), and **[LPtCNPhen**] (bottom right), as obtained from XRD analysis. Displacement ellipsoids are shown at 50% probability and hydrogen atoms are omitted for clarity. Further illustrations can be found in the Supporting Information (Figures S71–S74).

**Figure** **2** shows the unit cells of **[LPtCNtBu]**, **[LPtCNPhen]**, **[HLHgCl]**, and **[LAuCl]**. Interestingly, a commonality in all unit cells (disregarding the case of **[LPtCNPhen]**) is observed involving an antiparallel arrangement of dimers in the center, where each monomer is related to the other by a center of inversion. These dimers are stabilized by the weak *π*‐*π*‐interactions between the N^C^N‐ligands. As depicted in Figure 2, all unit cells comprise four or more molecules with the exception of **[LAuCl]**, where only two complexes are detected. In the case of **[LPtCNtBu]**, the four complexes are related to each other by a center of inversion, arranged in an alternating antiparallel fashion with the *t*Bu‐groups pointing toward the pyridine unit of the next neighbor and a Pt‐Pt‐Pt angle of 116° (within a unit cell). For **[HLHgCl]**, the two antiparallel‐arranged molecules (at the center of the unit cell) are surrounded by two other units located at the edges, almost perpendicular (82.3°) to those in the center with a Hg‐Hg‐Hg angle of 82°. Such orientations are favorable to minimize the repulsion between the bulky *t*Bu‐moieties. Presumably, for the same reason, the crystal structure of **[LPtCNtBu]** is characterized by the absence of Pt‐Pt interactions, with Pt‐Pt distances of 5.241 and 9.399 Å, respectively. In contrast, for **[LPtCNPhen]**, the consistent metal‐metal distance of 3.47 Å with a Pt‐Pt‐Pt angle of 155° (along a wire‐like structure) indicate $d_{z^{2}}$‐$d_{z^{2}}$ coupling, featuring long Pt‐Pt wires across the *c*‐axis. Furthermore, in single crystals, the molecules of **[LPtCNPhen]** are arranged with a plane‐to‐plane separation of 3.39 Å, where the complexes in each plane are related to the surroundings by a center of inversion. Hence, the molecules are congruent in every second level and slightly shifted by ≈38° between each stage, indicating moderate *π‐π*‐interactions.

### 2.3 Photophysical Characterization

All complexes were characterized by means of UV–vis absorption spectroscopy in conjunction with steady‐state and time‐resolved photoluminescence spectroscopy in liquid DCM solutions at RT (both air‐equilibrated and Ar‐purged), as well as in DCM/MeOH 1:1 frozen glassy matrices at 77 K, and in the crystalline phase by time‐resolved luminescence micro(spectro)scopy with multiphoton excitation.

**Figure 3:**
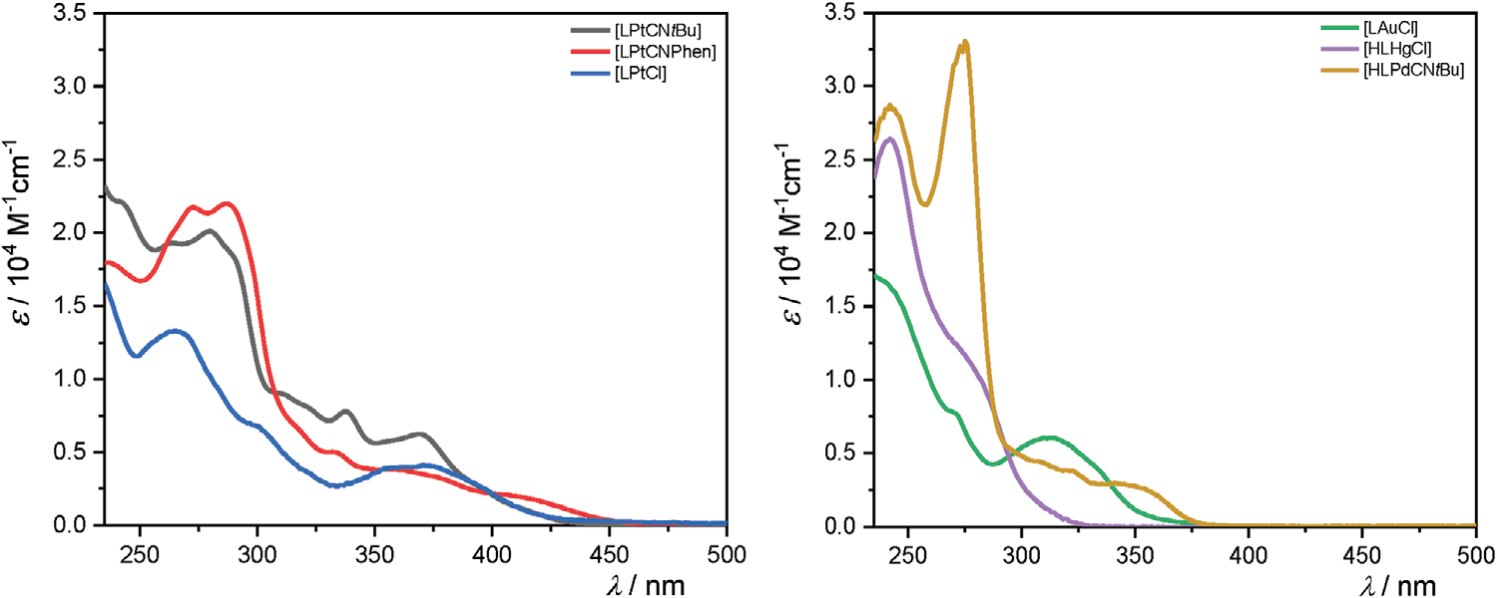
UV–vis absorption spectra (molar absorption coefficients as a function of wavelength) of the obtained complexes. Left: **[** **LPtCN** ***t*** **Bu]**, **[LPtCNPhen]**, and **[LPtCl]** in DCM. Right: **[LAuCl]**, **[HLHgCl]**, and **[HLPdCN** ***t*** **Bu]** in MeOH.

The UV–vis absorption spectra of the Pt(II) and Pd(II) complexes (**Figure** **3**) closely resemble those of related coordination compounds, where the bands below 300 nm can be generally ascribed to spin‐allowed transitions into mostly intra‐ligand singlet states (^1^LC, i.e., *π*‐π* configurations) with minor admixtures of metal‐to‐ligand‐charge‐transfer character (^1^MLCT, i.e., *d*‐π* configurations). The less intense group of bands between 325 and 450 nm can be assigned to transitions into singlet excited states with a predominant MLCT character, where the contribution of the metal‐based orbitals becomes more dominant.^[^ ^4^, ^44^, ^77^, ^102^ ^]^ In the case of the Au(III) and Hg(II) complexes, the spectra resemble those compounds where the low energy absorption bands below 300 nm can be ascribed to spin‐allowed transitions into mostly intra‐ligand singlet states (^1^LC, i.e., *π*‐π* configurations). The less intense absorption band centered at 312 nm in the case of **[LAuCl]** can be assigned to transitions into metal‐perturbed ligand‐centered states, as admixtures of ligand‐centered (LC, i.e., π‐π*) and ligand‐to‐ligand charge‐transfer (LLCT, i.e., *π*‐π*) configurations.^[^ ^103^, ^104^, ^105^, ^106^, ^107^, ^108^ ^]^

**Figure 4:**
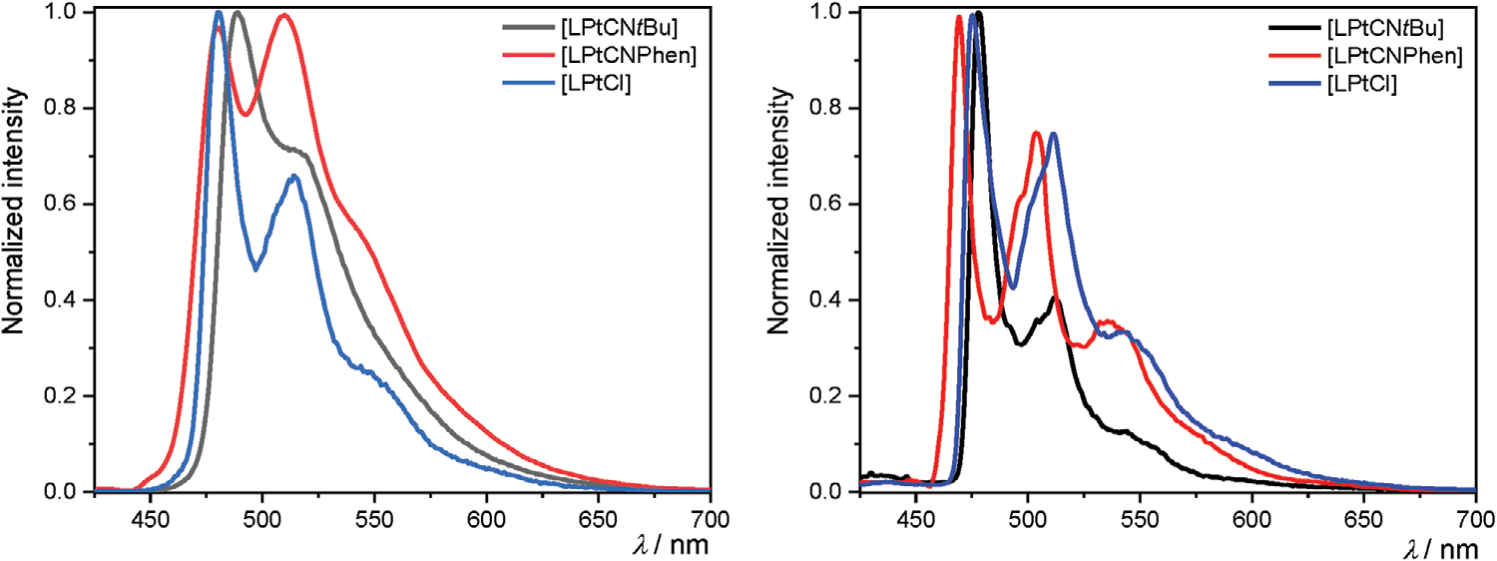
Normalized photoluminescence spectra of the Pt(II) complexes **[LPtCN** ***t*** **Bu]** (black), **[LPtCNPhen]** (red), and **[LPtCl]** (blue) in liquid DCM solutions at RT (left) and in a frozen DCM/MeOH 1:1 glassy matrix at 77 K (right).

To provide a more comprehensive explanation for the emission spectra, they will be herein categorized into two distinct groups. The first group corresponds to the Pt(II) complexes, namely **[LPtCN*t*Bu]**, **[LPtCNPhen]**, and **[LPtCl]**. The emission spectra in liquid DCM at RT, as well as in a glassy matrix at 77 K, are shown in **Figure** **4**. Only a minor shift is observed when varying the ancillary ligand (CN*t*Bu, CNPhen, or Cl), suggesting that both fragments, as well as the coordination environment, exert minimal influence (both in the position of maxima and in the vibrational progression), as previously reported for comparable compounds (e.g., with constitutional isomers of the main ligand such as C^N^N chelates).^[^ ^35^, ^61^ ^]^ Compared to RT, the main emission bands at 77 K appear slightly blue‐shifted (5–10 nm), which can be attributed to a weaker charge‐transfer stabilization and restricted solvent reorientation in the frozen glassy matrix. The obtained vibrational progression profiles (Figure 4) can be assigned to metal‐perturbed ligand‐centered triplet states (^3^MP‐LC states), as a combination of ^3^LC and ^3^MLCT character (ligand‐centered *π*‐π* and metal‐to‐ligand charge‐transfer, *d*‐π*, respectively), based on previous reports for other Pt(II) complexes.^[^ ^4^, ^44^, ^61^, ^77^, ^102^ ^]^ The sharper vibrational progression and the almost non‐blue‐shifted emission along with the longer excited state lifetimes at 77 K (**Table** **1**) pinpoints that the main contribution to the luminescence in glassy matrices arises from excited states with mostly ^3^LC character.

**Table 1:** Photoluminescence quantum yields (*Φ*_L_) and excited state lifetimes (*τ*) of the complexes in different conditions (air‐equilibrated or argon‐purged samples, room temperature, or frozen matrices at 77 K).

Complex	*τ*_air_/ns	*τ*_Ar_/ns	*τ*_77K_/µs	*Φ*_L/air_ ± 0.02	*Φ*_L/Ar_ ± 0.02	*Φ*_L/77K_ ± 0.04
**[HLPdCN*t*Bu]**	*τ*_1_ = 9.43 ± 0.07 (77%) *τ*_2_ = 2.5 ± 0.3 (23%) *τ*_av_amp_ = 7.9 ± 0.1	*τ*_1_ = 10.37 ± 0.08 (79%) *τ*_2_ = 2.1 ± 0.5 (21%) *τ*_av_amp_ = 8.6 ± 0.2	*τ*_1_ = 445 ± 3 (69%) *τ*_2_ = 200 ± 8 (31%) *τ*_av_amp_ = 370 ± 1	0.02	0.04	0.60
**[LPtCl]**	*τ* = 370.6 ± 0.5	*τ*_1_ = 1160 ± 20 (48%) *τ*_2_ = 5760 ± 10 (52%) *τ*_av_amp_ = 3570 ± 10	*τ*_1_ = 14.8 ± 0.4 (37%) *τ*_2_ = 7.9 ± 0.2 (63%) *τ*_av_amp_ = 10.45 ± 0.04	0.04	0.20	0.88
**[LPtCN*t*Bu]**	*τ* = 458.8 ± 0.4	*τ* = 1904 ± 2	*τ*_1_ = 9.4 ± 0.2 (61%) *τ*_2_ = 13.9 ± 0.2 (39%) *τ*_av_amp_ = 11.15 ± 0.02	0.02	0.11	0.94
**[LPtCNPhen]**	*τ*_1_ = 109.5 ± 0.9 (84%) *τ*_2_ = 742 ± 4 (16%) *τ*_av_amp_ = 210 ± 1	*τ* = 14 150 ± 40	*τ* = 14.37 ± 0.02	0.02	0.40	0.98
**[HLHgCl]**	*τ*_1_ = 1.504 ± 0.002 (94%) *τ*_2_ = 5.91 ± 0.03 (6%) *τ*_av_amp_ = 1.764 ± 0.007	*τ*_1_ = 5.74 ± 0.02 (8%) *τ*_2_ = 1.57 ± 0.03 (92%) *τ*_av_amp_ = 1.93 ± 0.02	*τ*_1_ = 10 200 ± 300 (16%) *τ*_2_ = 3420 ± 40 (84%) *τ*_av_amp_ = 4510 ± 20	0.02	0.02	0.65

In all cases, *c* ≈ 10^−5^ m. For the multiexponential photoluminescence decays, *τ_av_amp_* is shown along the fitted components. Raw time‐resolved photoluminescence decays, including the individual fitting components and their relative amplitudes (for biexponential decays), are available in Figures S74–S88, Supporting Information.

**Figure 5:**
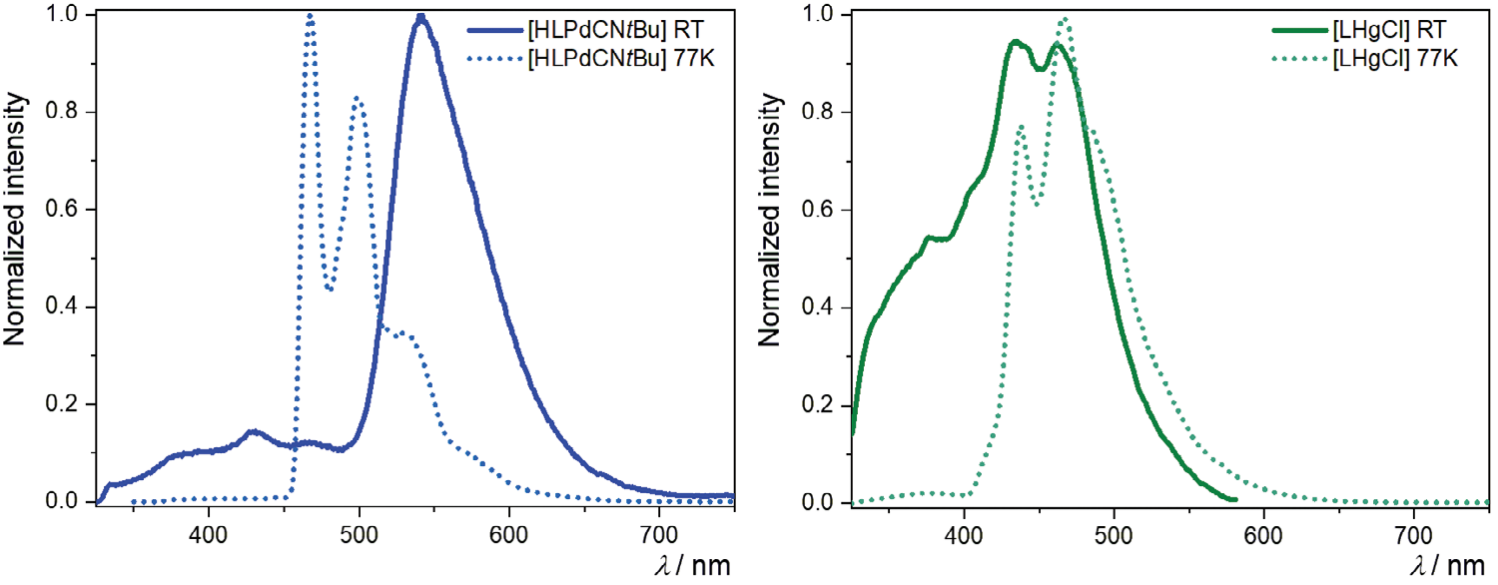
Photoluminescence spectra of the complexes **[HLPdCN** ***t*** **Bu]** (left, blue) and **[LHgCl]** (right, green) in liquid DCM solution at RT (solid lines) and in a DCM/MeOH 1:1 glassy matrix at 77 K (dotted lines).

For the Pd(II) complex **[HLPdCN*t*Bu]**, only a mild emission in the form of unstructured and broad bands was observed in liquid solutions at RT (**Figure** **5**). Due to the thermal accessibility of dissociative excited states, the formal population of antibonding 4$d_{x^{2}-y^{2}}$ orbitals is enabled. Thus, non‐radiative deactivation pathways through conical intersections with the ground state are favored. Consequently, this complex is almost non‐emissive at RT (as evidenced by the low quantum yield, *Φ*_L_, Table 1). However, when this complex is immersed in a glassy matrix at 77 K, due to the thermal inaccessibility of dark states, a vibrationally structured emission spectrum can be seen (Figure 4) with an enhancement of both *Φ*_L_ and *τ*. These results, together with our previous observations regarding luminescent Pd(II) complexes, suggest that the emission at RT stems from weakly coupled aggregates with predominant excimer character rather than from the corresponding monomeric species, considering the broad yet vibrationally unstructured emission profile together with the large red‐shifted emission, if compared with the Pt(II) complexes or with the Pd(II) complex at low temperatures.

In general, no luminescence was observed for **[LAuCl]** in any case, which can most probably be explained by transitions into a non‐emissive/dark LMCT (*π*‐d*) state upon photoexcitation. On the other hand, in the case of the Hg(II) complex **[HLHgCl]** in liquid DCM solution at RT, even though the metal center is sufficient to facilitate an efficient intersystem crossing process leading to an excited triplet state, it does not effectively enhance the radiative rate, resulting in a relative preference for radiationless deactivation processes. This is accompanied by broad unstructured blue‐shifted emission bands (Figure 5), low quantum yields, and short lifetimes. However, when the complex is immersed in a glassy matrix at a low temperature (77 K), the majority of radiationless pathways are suppressed. In this case, the relaxation of the excited state into the ground state can only occur via highly spin‐forbidden radiative pathways, as evidenced by the long excited‐state lifetimes (of the order of ms) with surprisingly high quantum yields (Table 1). When observing the emission spectrum of the complex in a glassy matrix at 77 K (Figure 5), a vibrational progression can be seen, suggesting that the emission arises from an excited LC state.

In addition, the absolute photoluminescence quantum yields (*Φ*_L_) and excited‐state lifetimes (*τ*) (Table 1) were measured and used to estimate the average radiative (*k*_r_) and non‐radiative (*k*_nr_) rate constants (**Table** **2**) according to the following equations and assuming a unitary intersystem crossing efficiency for *S*_1_ → *T*_n_ → *T*_1_ due to the chelation of late transition metal cations:

(1) $$k_{r}=k_{{\mathrm{ISC}}^{\prime }}=\frac{\Phi_{L}}{\tau_{L}}$$

(2) $$k_{\mathrm{nr}}=\frac{1-\Phi_{L}}{\tau_{L}}$$

where *k*_ISC´_ is the intersystem crossing rate constant (*T*_1_ → *S*_0_). For multiexponential decays, amplitude‐weighted average lifetimes (*τ*_av_amp_) were used to estimate the average rate constants.^[^ ^109^ ^]^

**Table 2:** Radiative (*k*_r_) and non‐radiative (*k*_nr_) rate constants of the complexes under different conditions (Ar‐purged samples at RT or frozen glassy matrices at 77 K).

Complex	*k_r_*_(Ar)_/10^4^ s^−1^ ^a)^	*k_nr_*_(Ar)_/10^4^ s^−1^**	*k_r_*_(77 K)_/10^4^ s^−1^**	*k_nr_*_(77 K)_/10^4^ s^−1^**
**[HLPdCN*t*Bu]**	< 233	11 400 ± 400	0.16 ± 0.01	0.11 ± 0.01
**[LPtCl]**	5.6 ± 0.6	22.4 ± 0.6	8.4 ± 0.2	1.1 ± 0.2
**[LPtCN*t*Bu]**	6 ± 1	44 ± 1	8.4 ± 0.2	0.5 ± 0.2
**[LPtCNPhen]**	2.8 ± 0.1	4.2 ± 0.1	6.7 ± 0.1	0.3 ± 0.1
**[HLHgCl]**	< 1036	52 000 ± 1000	0.014 ± 0.001	0.008 ± 0.001

^a)^ Estimated by using amplitude‐weighted average lifetimes *τ*_av_amp_. The uncertainties were estimated by using total differentials (see Supporting Information for further details).

Long‐lived excited states in liquid solution may undergo deactivation via diffusion‐controlled quenching mediated by ^3^O_2_, as observed for numerous transition metal complexes including those of Pt(II), Pd(II), and Re(I), among others.^[^ ^29^, ^44^, ^61^, ^81^ ^]^ For the Pt(II) complexes, purging the solutions with Ar results in a significant increase in both quantum yields and lifetimes, as listed in Table 1. Specifically, the data presented in Table 1 reveals a substantial increase ranging from 5‐ to 20‐fold, depending on the monodentate ligand. At 77 K, due to the suppression of rotovibrational degrees of freedom in frozen glassy matrices, an extensive increment in the lifetimes is observed, together with an almost unitary *Φ*_L_ for the complexes (Table 1). As shown in Table 2, while at RT there are some differences in *k*_r_ and *k*_nr_ depending on the monodentate ligand (and thus in the ligand field splitting), both *k*_r_ and *k*_nr_ are practically identical at 77 K for all the Pt(II) complexes, due to the rigidity of the system and virtual absence of solvent interaction.

In the case of the Pd(II) complexes and owing to a significantly lower ligand‐field splitting, the metal‐centered states represent dissociative excited states that become thermally accessible at RT. This enables a facile population of the antibonding 4$d_{x^{2}-y^{2}}$ orbitals. This effect ultimately favors a non‐radiative deactivation through conical intersections with the ground state, rendering these complexes practically non‐emissive in liquid solutions at RT. This is evidenced by short lifetimes, low *Φ*_L_, and higher *k*_nr_, irrespective of the presence or absence of ^3^O_2_. However, in a glassy matrix at 77 K, the thermal population of antibonding *d* ^*^ orbitals becomes inaccessible, resulting in longer lifetimes with higher quantum yields. This is evident along with a significant reduction in *k*_nr_, as shown in Tables 1 and 2, consistent with previous reports on Pd(II) complexes.^[^ ^81^ ^]^ Finally, in the case of the Hg(II) complex at RT, the sample is practically non‐emissive (see Table 2), as *k*_nr_ surpasses *k*_r_ (even in the absence of ^3^O_2_). However, at 77 K, the relaxation of the excited states into the ground state can only occur via highly spin‐forbidden radiative pathways with an *Φ*_L_ reaching 65% and *τ* in the ms range (Tables 1 and 2).

### 2.4 Time‐Resolved Multiphoton Micro(Spectro)scopy

In addition, time‐resolved multiphoton micro(spectro)scopy was used to obtain the photoluminescence maps (decay rates), as well as the emission spectra employing either single‐photon or two‐photon excitation of two Pt(II)‐based crystals, namely **[LPtCPhen]** and **[LPtCN*t*Bu]**.

**Figure 6:**
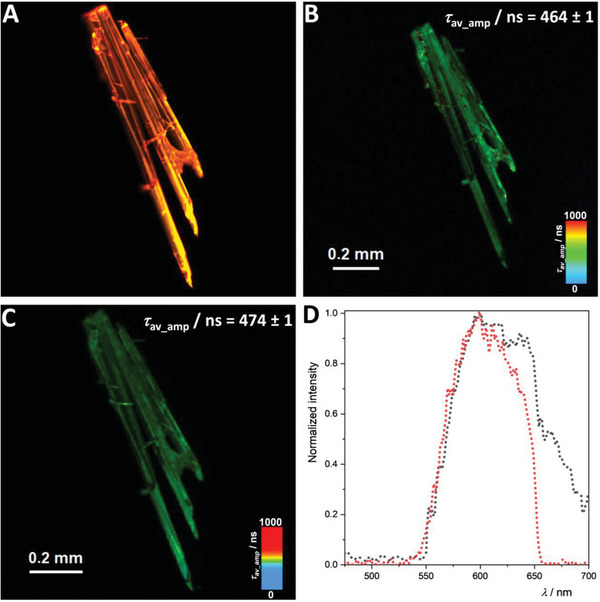
Time‐resolved photoluminescence micro(spectro)scopy imaging of **[LPtCNPhen]** crystals. A) Photoluminescence micrograph. B) Photoluminescence lifetime map of the crystalline phases of the complex using SPE (*λ*_ex_ = 377 nm, low‐pass cut‐off filter 514 LP). C) Photoluminescence lifetime map of the crystalline phases of the complex using TPE (*λ*_ex_ = 750 nm, low‐pass cut‐off filter 514 LP). D) Photoluminescence spectra measured with SPE (black dotted lines) and TPE (red dotted lines). Raw time‐resolved photoluminescence decays, including the individual fitting components and their relative amplitudes (for biexponential decays), are available in the Supporting Information (Figures S92 and S93).

**Figure 7:**
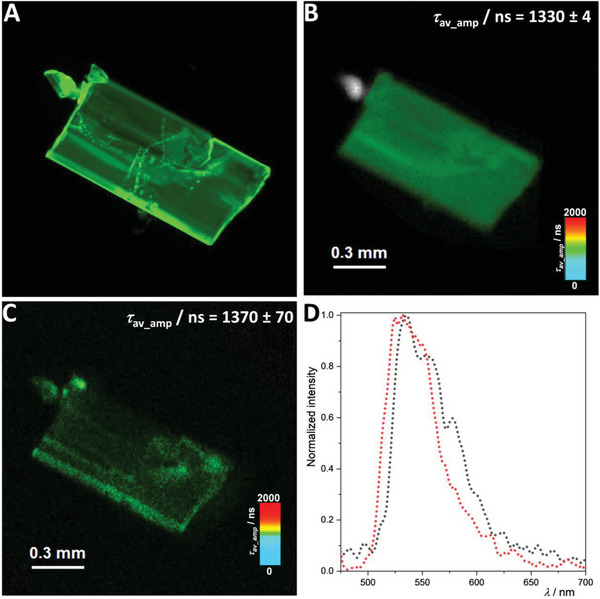
Time‐resolved photoluminescence micro(spectro)scopy imaging of **[LPtCN** ***t*** **Bu]** crystals. A) Photoluminescence micrograph. B) Photoluminescence lifetime map of the crystalline phase of the complex using SPE (*λ*_ex_ = 377 nm, low‐pass cut‐off filter 514 LP). C) Photoluminescence map of the crystalline phases of the complex using TPE (*λ*_ex_ = 750 nm, low‐pass cut‐off filter 514 LP). D) Photoluminescence spectra measured with SPE (black dotted lines) and TPE (red dotted lines). Raw time‐resolved photoluminescence decays including the individual fitting components and their relative amplitudes (for biexponential decays) are available in the Supporting Information (Figure S90 and S91).

From the structural analysis presented in the previous sections, a Pt‐Pt distance of 3.46 Å was obtained for **[LPtCNPhen]**. Thereby, Pt‐Pt interactions are allowed. As depicted in **Figure** **6**, distinct properties corresponding to aggregates (such as an orange luminescence, a broad emission band centered at around 600 nm, and shorter lifetimes) are obtained. Conversely, for **[LPtCN*t*Bu]**, where the Pt‐Pt distance exceeds the sum of van der Waals radii, the formation of aggregates is prevented, leading to the characteristic green emission and longer lifetimes of monomers, as shown in **Figure** **7**. Consistent results were observed whether SPE or TPE were used.

## 3 Conclusion

In this work, a convenient synthetic pathway toward a novel dianionic ligand providing a tridentate N^C^N‐type coordination pattern is demonstrated as a versatile platform for multiple phosphorescent metal complexes. The judicious choice of the central atom together with diverse ancillary ligands resulted in six novel coordination compounds encompassing Pd(II), Pt(II), Hg(II), and Au(III) centers. In general, all Pt(II)‐based compounds showed intense photoluminescence with only minor variations upon change of the co‐ligand, suggesting only a minimal influence from the ancillary moiety, due to the inherently strong ligand field exerted by the main luminophoric chelator. In addition, even though the Hg(II)‐based compound presented a weak emission in liquid solutions at RT, it showed enhanced photoluminescence efficiency upon cooling, with particularly drastic increases in both *Φ*_L_ and *τ*. To the best of our knowledge, this is the first time that such a highly emissive Hg(II) complex is described, with a notably prolonged excited state lifetime.

In summary, the novel luminophoric ligand provides a convenient toolbox for the realization of phosphorescent emitters based on *d* ^8^‐configured metal cations. Moreover, our findings also demonstrate that the herein‐reported design pattern enables the realization of phosphorescent coordination compounds implementing a *d* ^10^‐configured species as well. These outcomes hold significant interest, particularly in light of potential applications across diverse fields including photocatalysis, photodynamic therapy, oxygen sensing, and as substitutes for Ir(III)‐based emitters in optoelectronic devices. Thus, these new coordination‐chemical concepts could pave the road toward cost‐efficient metal complexes utilizing more abundant and hence economically advantageous materials.^[^ ^90^, ^91^ ^]^

## Conflict of Interest

The authors declare no conflict of interest.

## Supporting information

Supporting Information

## Data Availability

The data that support the findings of this study are available in the supplementary material of this article.
