# Supplementary material for: One Dianionic Luminophore with Three Coordination Modes Binding Four Different Metals: Toward Unexpectedly Phosphorescent Transition Metal Complexes
Source: Adv Sci (Weinh). 2023 Dec 31;11(11):2306801. doi: 10.1002/advs.202306801 (PMC10953592; doi:10.1002/advs.202306801)
Supplement: Supplementary file 1 — Supporting Information [file ADVS-11-2306801-s001.pdf]

ADVANCED SCIENCE
Open Access

Supporting Information

for *Adv. Sci.*, DOI 10.1002/advs.202306801

One Dianionic Luminophore with Three Coordination Modes Binding Four Different Metals: Toward Unexpectedly Phosphorescent Transition Metal Complexes

*Thomas M. Kirse, Iván Maisuls, Leander Spierling, Alexander Hepp, Jutta Kösters and Cristian A. Strassert**

Supporting Information

One Dianionic Luminophore with Three Coordination Modes Binding Four Different Metals: Towards Unexpectedly Phosphorescent Transition Metal Complexes

Thomas M. Kirse[a][b]¥, Iván Maisuls[a][b]¥, Alexander Hepp[a], Jutta Kösters [a], and Cristian A. Strassert [a][b]*

[a] Institut für Anorganische und Analytische Chemie, Universität Münster, Corrensstr. 28/30, 48149, Münster, Germany

[b] CiMiC, SoN and CeNTech, Universität Münster, Heisenbergstr. 11, 48149, Münster, Germany

eMail: ca.s@uni-muenster.de

¥These authors contributed equally to this work

Table of content

Materials and methods	S2
Synthesis and characterization	S5
NMR spectroscopy and mass spectrometry	S13
X-ray diffractometry on single crystals	S46
Photophysical studies	S52
References	S61

Materials and methods

Commercially available reagents were used without further purification. Silica gel 60 (0.063 – 0.200 mm) for column chromatography was purchased from Merck (mentioned as silica) standardized for column chromatography adsorption analysis acc. to Brockmann from Merck were used for column chromatography, if not otherwise stated.

NMR spectra and exact mass

NMR-spectra were obtained at the Institut für Anorganische und Analytische Chemie (Univ. Münster), using a Bruker AVANCE NEO/ Bruker AVANCE I/ Bruker AVANCE III (400 MHz) or a Bruker AVANCE NEO (500 MHz). All measurements were performed at 300 K unless mentioned otherwise. The ^{1}H-NMR and ^{13}C-NMR chemical shifts (δ) of the signals are given in parts per million and are referenced to the residual signal of the deuterated solvent. The signal multiplicities are abbreviated as follows: s, singlet; d, doublet; t, triplet; q, quartet; m, multiplet. ^{1}H NMR chemical shifts are given relative to TMS and are referenced to the solvent signal. Spectra of other nuclides like ^{13}C, ^{15}N, ^{19}F and ^{31}P are referenced according to the proton resonance of TMS as the primary reference for the unified chemical shift scale (IUPAC recommendation 2001).[1] All coupling constants (J) are given in Hertz (Hz).
Exact mass (EM) determination by mass spectrometry (MS) was carried out at the Organisch-Chemisches Institut (Univ. Münster) using a LTQ Orbitap LTQ XL (Thermo-Fisher Scientific, Bremen) with electrospray injection (ESI).

Photophysical measurements

Absorption spectra were measured with a Shimadzu UV-3600 I plus UV-VIS-NIR spectrophotometer.

Photoluminescence quantum yields were measured with a Hamamatsu Photonics absolute PL quantum yield measurement system (C9920-02) equipped with a L9799-01 CW Xe light source (150 W), a monochromator, a C7473 photonic multi-channel analyzer, an integrating sphere and employing U6039-05 software (Hamamatsu Photonics, Ltd., Shizuoka, Japan).

Steady-state excitation and emission spectra were recorded on a FluoTime 300 spectrometer from PicoQuant equipped with: a 300 W ozone-free Xe lamp (250-900 nm), a 10 W Xe flash-lamp (250-900 nm, pulse width *ca.* 1 µs) with repetition rates of 0.1 – 300 Hz, a double-grating

excitation monocromator (Czerny-Turner type, grating with 1200 lines/mm, blaze wavelength: 300 nm), diode lasers (pulse width < 80 ps) operated by a computer-controlled laser driver PDL-828 “Sepia II” (repetition rate up to 80 MHz, burst mode for slow and weak decays), two double-grating emission monochromators (Czerny-Turner, selectable gratings blazed at 500 nm with 2.7 nm/mm dispersion and 1200 lines/mm, or blazed at 1200 nm with 5.4 nm/mm dispersion and 600 lines/mm) with adjustable slit width between 25 μm and 7 mm, Glan-Thompson polarizers for excitation (after the Xe-lamps) and emission (after the sample). Different sample holders (Peltier-cooled mounting unit ranging from -15 to 110 °C or an adjustable front-face sample holder), along with two detectors (namely a PMA Hybrid-07 from PicoQuant with transit time spread FWHM < 50 ps, 200 – 850 nm, or a H10330C-45-C3 NIR detector with transit time spread FWHM 0.4 ns, 950-1700 nm from Hamamatsu) were used. Steady-state spectra and photoluminescence lifetimes were recorded in TCSPC mode by a PicoHarp 300 (minimum base resolution 4 ps) or in MCS mode by a TimeHarp 260 (where up to several ms can be traced). Emission and excitation spectra were corrected for source intensity (lamp and grating) by standard correction curves. For samples with lifetimes in the ns order, an instrument response function calibration (IRF) was performed using a diluted Ludox® dispersion. Lifetime analysis was performed using the commercial EasyTau 2 software (PicoQuant). The quality of the fit was assessed by minimizing the reduced chi squared function (χ^2) and visual inspection of the weighted residuals and their autocorrelation.
All solvents used were of spectrometric grade (Uvasol®, Merck).

Time-resolved multiphoton micro(spectro)scopy

Time-resolved multiphoton micro(spectro)scopy was carried out on a fluorescence microscope (IX 73 from Olympus) with a complete confocal system and a laser combining unit (LCU), an inverted microscope body and a multichannel detection unit (MicroTime 200, PicoQuant) equipped with diode lasers (providing adjustable output power and repetition rates up to 80 MHz inside a compact fibre couple unit with wavelengths between 375 and 900 nm). For beam diagnostics, a charge-couple device (CCD) camera and a photodiode were available in the main optical unit (MOU) of the microscope. The MOU is equipped with two detectors, namely a hybrid photomultiplier-based single photon counting module (PMA Hybrid 40, PicoQuant) and a SPAD-based photon counting module (SPCM-AQR-14, Perkin-Elmer). Different band-pass (BP) and low-pass (LP) filters were placed before these detectors on demand to acquire lifetime maps. Data acquisition is based on the unique time-tagged time-resolved (TTTR) measurement

mode, where simultaneous data acquisition on two channels is possible. Data were processed and analysed with the SymphoTime 64 (PicoQuant) software. In order to couple the MicroTime 200 and the FluoTime 300 instruments, a fiber coupler was employed. In this way, the spectrometer can be used to record either steady-state or time-resolved luminescence spectra and decays from a sample mounted on the microscope. Luminescence micrographs were acquired using the same microscope mentioned above, equipped with a X-CiteQ Lamp module (Excelitas Technologies) as the excitation source and a UI-5580SE (IDS) digital camera. Different band pass (BP) and low pass (LP) cubes were using accordingly.

Additionally, a two-photon (Spectra-Physics Mai Tai®) Ti:Sapphire (Ti:Sa oscillator) laser with a pulse with <100 fs and tuning range of 690 – 1040 nm, coupled to the MOU can be used as the excitation source. In order to reduce the repetition rate, the Ti:Sapphire laser was connected to a double pulse picker (A.P.E.®).

X-ray diffraction measurements on single crystals

X-ray diffractometry experiments on single crystals were carried out at the Institut für Anorganische und Analytische Chemie (Univ. Münster). The measurements were performed on a Bruker D8 Venture single crystal X-ray diffractometer equipped with a PHOTON III CMOS detector (Mo as the X-ray target material, k_{α} = 0.71073 Å). Diffraction frames were recorded with the APEX4 software package (version 2021.4.0, Bruker AXS Inc., Madison, Wisconsin). Data integration and absorption corrections were achieved with SAINT (version 8.40, Bruker AXS Inc., Madison, Wisconsin) and SADABS (version 2016/2, Bruker AXS Inc., Madison, Wisconsin). The plots of molecular structures in the crystals and of the unit cells were created with Mercury (Cambridge Crystallographic Data Center (CCDC), Cambridge, England).

Synthesis and characterization

1.1 Synthesis of methyl 3-(pyridin-2-yl)benzoate [1]

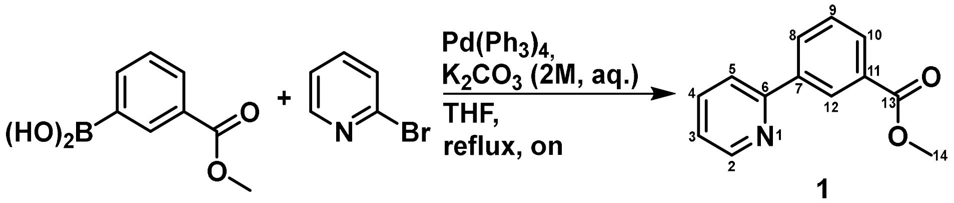

1

In an oven-dried SCHLENK-tube, 2-bromopyridine (2.7 mL, 27.8 mmol, 1.0 eq.) and (3-(methoxycarbonyl)-phenyl)boronic acid (5.00 g, 27.8 mmol, 1.0 eq.) were dissolved in THF (210 mL) and tetrakis(triphenylphosphane)palladium (3.21 g, 2.78 mmol, 0.1 eq) was added. The solution was deoxygenated with argon for 10 min and K_2CO_3 (2M, 42 mL) was added. The reaction mixture was further deoxygenated with argon for 5 min. After refluxing overnight, the solution was cooled to RT, distilled H_2O (70 mL) was added, and the two phases were separated. The aqueous phase was extracted with EtOAc (3 x 40 mL). The combined organic phases were dried over Na_2SO_4 and the solvent was removed under reduced pressure. The crude product was purified by column chromatography (CyH/EtOAc, 9/1 - 7/3). **1** was obtained as colourless oil.

Yield: 4.74 g, 22.2 mmol, 80%.

^{1}H-NMR (400 MHz, acetone-d_6): δ (ppm) = 8.79 (t, $^4J_{HH}$ = 2 Hz, H1, H12), 8.71 (d, $^3J_{HH}$ = 4.8 Hz, 1H, H2), 8.36 (d, $^3J_{HH}$ = 7.9 Hz, 1H, H8), 8.06 (d, $^3J_{HH}$ = 7.9 Hz, 1H, H10), 8.00 (d, $^3J_{HH}$=8.0 Hz, 1H, H5), 7.90 (t, $^3J_{HH}$ = 7.9 Hz, 1H, H4), 7.63 (t, $^3J_{HH}$ = 7.9 Hz, 1H, H9), 7.37 (dd, $^3J_{HH}$ = 7.6, 4.8 Hz, 1H, H3), 3.93 (s, 3H, H14).

^{13}C-NMR (101 MHz, acetone-d_6): δ (ppm) = 167.2 (C13), 156.5 (C6), 150.7 (C2), 140.6 (C7), 138.0 (C4), 131.8 (C8), 131.8 (C11), 130.5 (C10), 129.9 (C9), 128.4 (C12), 123.7 (C3), 121.1 (C5), 52.5 (C14).

^{15}N-NMR (41 MHz, acetone-d_6): δ (ppm) = 310 (N1).

EM-MS-ESI (MeOH, $C_{13}H_{11}NO_2$, *m/z*): calcd. for $[C_{13}H_{11}NO_2+H]^+$ = 214.08626, found $[\mathbf{1}+H]^+$ = 214.08647.

1.2 Synthesis of 3-(pyridin-2-yl)benzohydrazide [2]

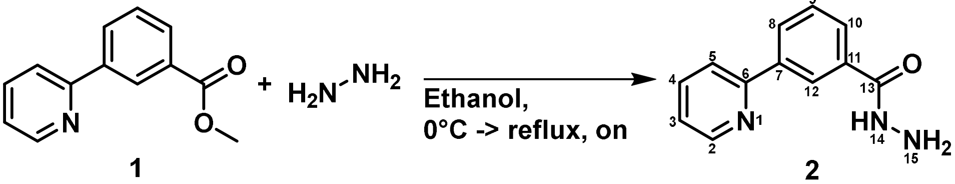

1 2

1 (3.80 g, 17.8 mmol, 1.0 eq.) was dissolved in EtOH (15 mL), the solution was cooled to 0°C and hydrazine hydrate (50%, 11.2 mL, 178 mmol, 10 eq.) was added. After stirring for 2 h at

0°C, the ice bath was removed and the solution was refluxed overnight. The next day, the solution was cooled to RT and the solvent was reduced to a minimum amount under reduced pressure. The product was precipitated by addition of Et_2O, separated and further washed with Et_2O, to obtain **2** as white powder.

Yield: 3.76 g, 17.6 mmol, 99%.

^{1}H-NMR (400 MHz, DMSO-d_6): δ (ppm) = 9.90 (s, 1H, H14), 8.69 (d, $^3J_{HH}$ = 4.8 Hz, 1H, H2), 8.53 (t, $^3J_{HH}$ = 1.8 Hz, 1H, H12), 8.22 (d, $^3J_{HH}$ = 7.9 Hz, 1H, H8), 8.03 (d, $^3J_{HH}$ = 8.0 Hz, 1H, H5), 7.93 (t, $^3J_{HH}$ = 7.6 Hz, 1H, H4), 7.88 (d, $^3J_{HH}$ = 7.88 Hz, 1H, H10), 7.57 (t, $^3J_{HH}$ = 7.8 Hz, 1H, H9), 7.39 (dd, $^3J_{HH}$ = 7.6, 4.8 Hz, 1H, H3), 2.50 (br. s, 2H, H15).

^{13}C NMR (101 MHz, DMSO-d_6): δ (ppm) = 165.6 (C13), 155.3 (C6), 149.5 (C2), 138.7 (C7), 137.3 (C4), 133.8 (C11), 128.9 (C8), 128.8 (C9), 127.5 (C10), 125.1 (C12), 122.8 (C3), 120.4 (C5).

^{15}N-NMR (41 MHz, DMSO-d_6): δ (ppm) = 309 (N1), 129 (N14).

EM-MS-ESI (MeOH, $C_{12}H_{11}N_3O$, *m/z*): calcd. for $[C_{12}H_{11}N_3O+H]^+$ = 214.09749, found $[\mathbf{2}+H]^+$ = 214.09750.

1.3 Synthesis of 2-(3-(3-(*tert*-butyl)-1*H*-1,2,4-triazol-5-yl)phenyl)pyridine [H_2L]

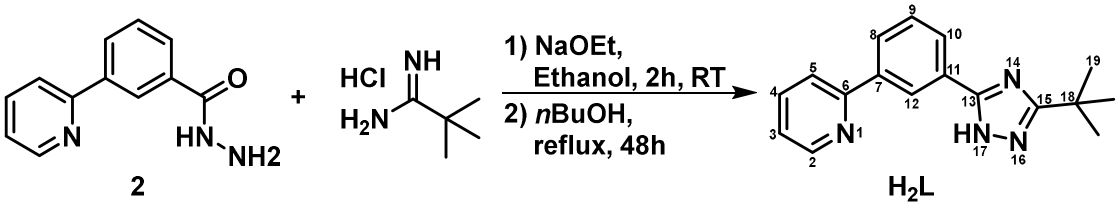

To a solution of pivalamidine hydrochloride (2.58 g, 18.9 mmol, 1.3 eq.) in a minimum of dry EtOH, sodium ethoxide (1.29 g, 18.9 mmol, 1.3 eq.) was added under argon atmosphere. After stirring for two hours at RT, the white precipitate was filtered off, washed with dry EtOH and the solvent of the filtrate was removed under reduced pressure. The residue was dissolved in *n*-BuOH (~20 mL) and **2** (3.09 g, 14.5 mmol, 1.0 eq.) was added under argon atmosphere. After stirring for 48 h at 130°C, the solution was cooled to RT and the solvent was removed under reduced pressure. The crude product was purified by column chromatography (CyH/EtOAc; 9/1-6/4)). **H_2L** was obtained as white powder.

Yield: 2.62 g, 9.4 mmol, 65%.

^{1}H-NMR (400 MHz, acetone-d_6): δ (ppm) = 12.86 (br. s, 1H, H17), 8.90(s, 1H, H12), 8.71 (dd, $^3J_{HH}$ = 4.8Hz, $^4J_{HH}$ = 1.8 Hz, 1H, H2) 8.17 (dd, $^3J_{HH}$ = 7.5 Hz, 1H, H10), 8.13 (dd, $^3J_{HH}$ = 7.5 Hz, 1H, H8), 7.97 (dd, $^3J_{HH}$ = 8.0 Hz, 1H, H5), 7.89 (td, $^3J_{HH}$ = 7.8Hz, $^4J_{HH}$ = 1.8 Hz, 1H, H4), 7.57(t, $^3J_{HH}$ = 7.5 Hz, 1H, H9), 1.46(s, 9H, H19).

^{13}C NMR (101 MHz, acetone-d_6): δ (ppm) = 166.2 (C15), 162.1 (C13), 157.5 (C6), 150.6 (C2), 140.5 (C7), 137.8 (C4), 129.7 (C9), 127.7 (C8), 127.5 (C10), 125.3 (C12), 123.4 (C3), 121.0 (C5), 33.0 (C18), 29.6 (C19).

^{15}N-NMR (41 MHz, acetone-d_6): δ (ppm) = 311 (N1).

EM-MS-ESI (MeOH, $C_{17}H_{18}N_4$, *m/z*): calcd. for $[C_{17}H_{18}N_4+H]^+$ = 279.16042, found [$\mathbf{H_2L}$+H]$^+$ = 279.16005.

2 Synthesis of [LPtCl]

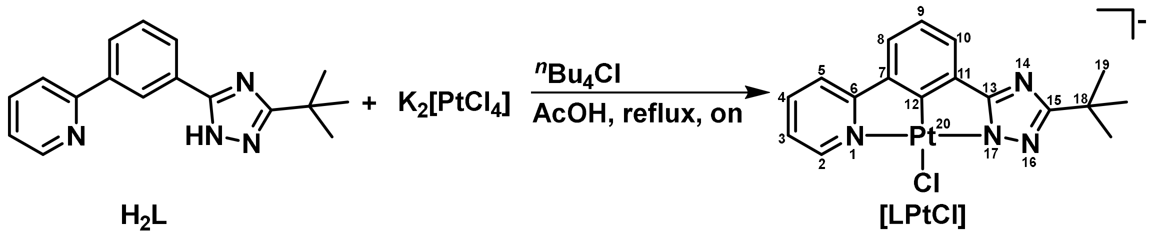

H2L (100.0 mg, 0.36 mmol, 1.0 eq.) and $K_2[PtCl_4]$ (149.4 mg, 0.36 mmol, 1.0 eq.) together with tetrabutylammonium chloride ($^nBu_4Cl$, 100.1 mg,0.36 mmol, 1 eq) were dissolved in acetic acid (20 mL), the solution was subsequently deoxygenated with argon for 15 min and refluxed overnight. The next day, the resulting precipitate was collected, washed with H_2O and Et_2O to obtain **[LPtCl]** (109.7 mg, 0.22 mmol, 60%) as yellow solid.

^{1}H-NMR (400 MHz, DMF-d_7): δ(ppm) = 9.46 (d, sat., $^3J_{PtH}$ = 42 Hz, $^3J_{HH}$ = 5.9 Hz, 1H, H2), 8.25 (td, $^3J_{HH}$ = 7.7 Hz, $^4J_{HH}$ = 1.6 Hz, 1H, H4), 8.15 (d, $^3J_{HH}$ = 7.8 Hz, 1H, H5), 7.78 (d, $^3J_{HH}$ = 7.8 Hz, 1H, H8), 7.58 (m, overlap., 2H, H3/10), 7.30 (t, $^3J_{HH}$ = 7.7 Hz, 1H, H3), 1.47 (s, 9H, H19).

^{13}C-NMR (101 MHz, DMF-d_7): δ (ppm) = 167.3 (C6), 165.1 (br., C13), 161.9 (C15, br.), 161.7 (C12), 151.2 (C2), 142.1 (C7), 140.5 (C4), 129.6 (br., C11), 124.5 (C8), 124.3 (C3), 123.8 (C9), 123.4 (C3), 121.0 (C5), 33.2 (C18), 29.0 (C19).

^{195}Pt-NMR (86 MHz, DMF-d_7): δ (ppm) = -3593 (s, Pt20).

EM-MS-ESI (MeOH, $C_{17}H_{16}N_4ClPt$, *m/z*): calcd. for $[C_{17}H_{16}N_4ClPt]^-$ = 506.07180, found [**[LPtCl]**]$^-$ = 506.07266.

3 Synthesis of [LPtCNPhen]

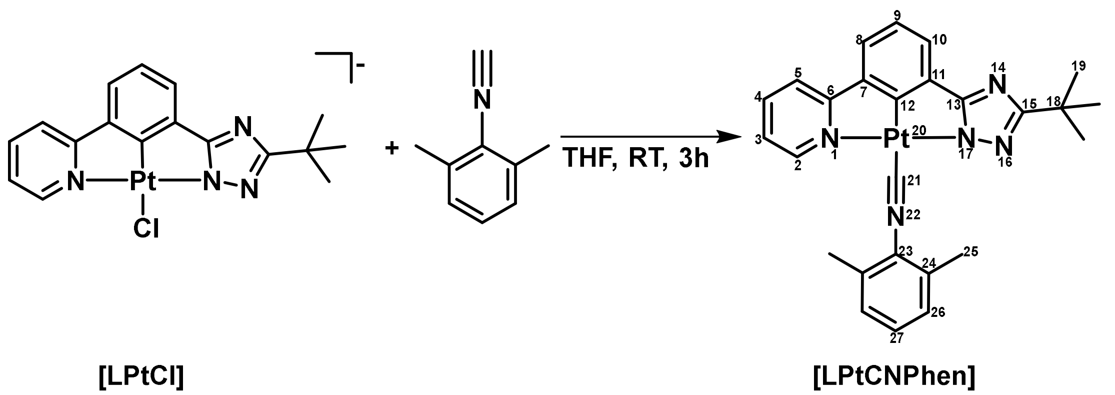

[LPtCl] (196.6 mg, 0.36 mmol, 1.0 eq.) was suspended in THF (40 mL), the suspension was deoxygenated with argon for 15 min and 2,6-dimethyl-phenylisonitrile (**CNPhen**, 47 mg, 0.36 mmol, 1.0 eq.) was added. After stirring for three hours at room temperature, the solid was

separated and the crude product was purified by column chromatography (DCM/MeOH; 99/1-93/7). **[LPtCNPhen]** (152.3 mg, 0.25 mmol, 70%) was obtained as orange-red solid.

^{1}H-NMR (500 MHz, $CDCl_3$/methanol-d_4): δ (ppm) = 8.45 (d, $^{3}J_{PtH}$ = 35 Hz, $^{3}J_{HH}$ = 5.8 Hz, 1H, H2) 7.76 (t, $^{3}J_{HH}$ = 7.9 Hz, 1H, H4), 7.46 (d, $^{3}J_{HH}$ = 7.8 Hz, 1H, H5), 7.34 (d, $^{3}J_{HH}$ = 7.4 Hz, 1H, H10), 7.15 (t, $^{3}J_{HH}$ = 7.6 Hz, 1H, H27), 7.10 (d, $^{3}J_{HH}$ = 7.7 Hz, 1H, H8), 7.01 (d, overlap, 2H, H26), 7.00 (t, overlap. 1H, H9), 6.91 (dd, $^{3}J_{HH}$ = 7.6 Hz, $^{3}J_{HH}$ = 5.8 Hz, 1H, H3), 2.43 (s, 6H, H25), 1.30 (s, 9H, H19).

^{13}C-NMR (126 MHz, $CDCl_3$/methanol-d_4): δ (ppm) = 171.1 (C15), 170.6 (C13), 168.9 (C12), 167.9 (C21), 153.7 (C2), 143.1 (C11), 139.1 (C4), 135.8 (C7), 135.6 (C24), 129.5 (C27), 127.9 (C26), 126.1 (C23), 126.1 (C23), 126.0 (C9), 123.8 (C3), 122.1 (C10), 120.9 (C8), 120.1 (C5), 32.8 (C18), 29.5 (C19), 18.6 (C25).

^{195}Pt-NMR (107 MHz, $CDCl_3$/methanol-d_4): δ (ppm) = -3789 (Pt20).

EM-MS-ESI (MeOH, $C_{26}H_{25}N_5Pt$, *m/z*): calcd. for $[C_{26}H_{25}N_5Pt+H]^+$ = 603.18325, found. [**[LPtCNPhen]**+H]$^+$ = 603.18210.

4 Synthesis of [LPtCN*t*Bu]

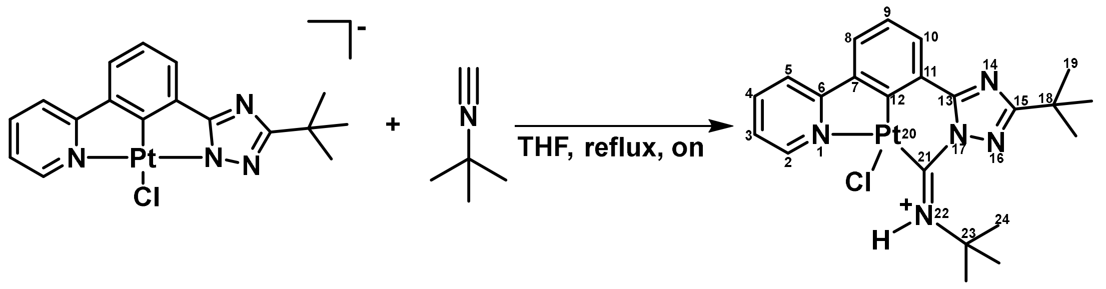

[LPtCl] (196.6 mg, 0.36 mmol, 1.0 eq.) was suspended in THF (40 mL), the suspension was deoxygenated with argon, *tert*-butyl isonitrile (0.04 mL, 0.36 mmol, 1.0 eq.) was added and the reaction mixture was stirred at 80°C for overnight. After cooling to RT, the solvent was removed under reduced pressure and the crude product was purified by column chromatography (CyH/EtOAc; 8:2–6:4). **[LPtCN*t*Bu]** (138.3 mg, 0.23 mmol, 65%) was obtained as yellow solid.

^{1}H NMR (400 MHz, DCM-d_2): δ (ppm) = 10.70 (s, br., 1H, H22), 9.57 (d, $^{3}J_{HH}$ = 5.5 Hz, $^{3}J_{HPt}$ = 11 Hz, 1H, H2), 8.24 (dd, $^{3}J_{HH}$ = 7.8 Hz, $^{4}J_{HH}$ = 1.4 Hz, 1H, H10), 7.93 (td, $^{3}J_{HH}$ = 7.8 Hz, $^{4}J_{HH}$ = 1.6 Hz, 1H, H4), 7.82 (d, $^{3}J_{HH}$ = 7.8 Hz, 1H, H5), 7.70 (d, $^{3}J_{HH}$ = 7.8 Hz, 1H, H8) , 7.34 (m, overlap., 1H, H3), 7.31 (m, overlap., 1H, H9), 1.68 (s, 9H, H24), 1.45 (s, 9H, H19).

^{13}C NMR (101 MHz, DCM-d_2): δ (ppm) = 173.7 (C15), 169.2 (sat., $^{1}J_{CPt}$ = 1433 Hz, C21), 164.9 (C13), 164.7 (C6), 151.5 (sat, $^{2}J_{CPt}$ = 17 Hz, C2,), 145.8 (C7), 144.9 (C11), 140.2 (C4), 129.7 (sat., $^{3}J_{HPt}$ = 51 Hz C10), 128.3 (C12), 126.4 (sat., $^{3}J_{CPt}$ = 44 Hz, C8), 124.6 (C9), 123.5

(sat., $^3J_{CPt}$ = 19 Hz ,C3), 119.2 (sat., $^3J_{CPt}$ = 29 Hz C5), 63.0 (sat., $^2J_{HPt}$ = 36 Hz, C23), 33.6 (C18), 30.1 (C24), 29.2 (C19).

^{15}N-NMR (41 MHz, DCM-*d*$_2$): δ (ppm) = 237 (N1), 200 (N22).

^{195}Pt NMR (86 MHz, DCM-*d*$_2$): δ (ppm) = -3695 (Pt20).

EM-MS-ESI (MeOH, $C_{22}H_{26}N_5PtCl$, *m/z*): calcd. for [**[LPtCN*t*Bu]**+Na]$^+$ = 613.14183, found. [**[LPtCN*t*Bu]**+Na]$^+$ = 613.14229.

6 Synthesis of [HLPdCN*t*Bu]

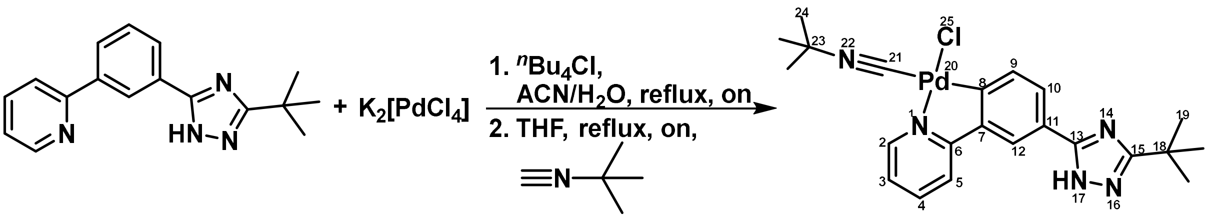

H$_2$L (100.0 mg, 0.36 mmol, 1.0 eq.) and $K_2[PdCl_4]$ (117.2 mg, 0.36 mmol, 1.0 eq.) together with tetrabutylammonium chloride (^{n}Bu$_4$Cl, 100.1 mg,0.36 mmol, 1 eq) were dissolved in a mixture of ACN/H_2O (1:1, 20 mL), the solution was subsequently deoxygenated with argon for 15 min and refluxed overnight. The next day the solvent was removed under reduced pressure, the obtained yellowish solid was suspended in THF (40 mL), the solution was purged with argon for 15 min and *tert*-butyl isonitrile (0.04 mL, 0.36 mmol, 1.0 eq.) was added. After refluxing for overnight, the solvent was removed under reduced pressure and the crude product was purified by column chromatography (CyH/EtOAc; 8:2 – 2:8). **[HLPdCN*t*Bu]** (63.3 mg, 0.13 mmol, 35%) was obtained as yellow solid.

^{1}H-NMR (500 MHz, acetone-*d*$_6$): δ (ppm) = 12.79 (s, br., 1H, H17), 9.36 (d, $^3J_{HH}$ = 5.7 Hz, 1H, H2), 8.36 (d, $^3J_{HH}$ = 1.8 Hz, 1H, H12), 8.12 (t, $^3J_{HH}$ = 7.3 Hz, 1H, H3), 8.07 (d, $^3J_{HH}$ = 7.8 Hz, 1H, H5), 7.84 (dd, $^3J_{HH}$ = 7.9 Hz, $^4J_{HH}$ = 1.8 Hz, 1H, H10) 7.49 (d, $^3J_{HH}$ = 7.9 Hz, 1H, H9), 7.47 (t, $^3J_{HH}$ = 6.5 Hz, 1H, H4), 1.72 (s, 9H, H24), 1.45 (s, 9H, H19).

^{13}C-NMR (126 MHz, acetone-*d*$_6$): δ (ppm) = 166.0 (C6), 156.3 (C13), 150.6 (C2), 147.0 (C7), 141.4 (C3), 138.7 (C9), 131.8 (pseudo t, $^1J_{NC}$ = 19.0 Hz, C21), 128.7 (C10), 123.7 (C4), 122.4 (C12), 120.0 (C5), 59.8 (C23), 33.0 (C18), 30.1 (C24), 29.6 (C19).

^{15}N-NMR (51 MHz, acetone-*d*$_6$): δ (ppm) = 193 (N22).

EM-MS-ESI (MeOH, $C_{22}H_{26}ClN_5Pd$, *m/z*): calcd. for [$C_{22}H_{26}ClN_5Pd$-Cl]$^+$ = 468.12219, found. [**[HLPdCN*t*Bu]**-Cl]$^+$ = 468.12261.

6 Synthesis of [HLHgCl]

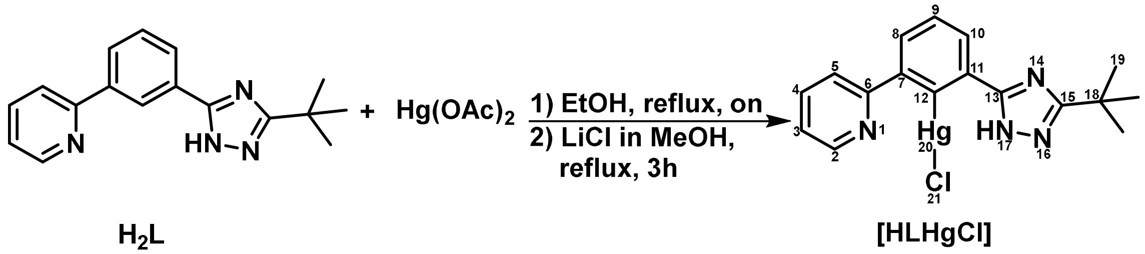

H_2L (150 mg, 0.54 mmol, 1 eq.) and $Hg(OAc)_2$ (172.7 mg, 0.54 mmol, 1 eq.) were dissolved in EtOH (15 mL, dry), the solution was refluxed for overnight. The next day, the solution was cooled to RT and LiCl (50.25 mg, 1.19 mmol, 2.2 eq.) dissolved in a minimum amount of MeOH was added. After refluxing for 3 h, the solution was cooled to RT and H_2O was added until no further precipitate was obtained. The resulting precipitate was collected, washed with H_2O and Et_2O to obtain **[HLHgCl]** (194.1 mg, 0.38 mmol, 70%) as white powder.

^{1}H NMR (400 MHz, DCM-d_2): δ (ppm) = 10.68 (s, br., 1H, H17), 8.68 (d, $^{3}J_{HH}$ = 4.8 Hz, 1H, H2), 8.25 (d, $^{3}J_{HH}$ = 7.0 Hz, 1H, H10), 8.00 (d, $^{3}J_{HH}$ = 7.7 Hz, 1H, H8), 7.91 (d, $^{3}J_{HH}$ = 8.1 Hz, 1H, H5), 7.84 (td, $^{3}J_{HH}$ = 7.6 Hz, $^{4}J_{HH}$ = 1.8 Hz, 1H, H4), 7.49 (t, $^{3}J_{HH}$ = 7.7 Hz, 1H, H9), 7.37 (ddd, $^{3}J_{HH}$ = 7.5 Hz, $^{3}J_{HH}$ = 4.9 Hz, $^{4}J_{HH}$ = 1.1 Hz, 1H, H3), 1.47 (s, 9H, H19).

^{13}C NMR (101 MHz, DCM-d_2): δ (ppm) = 166.8 (C15), 163.5 (C13), 158.1 (C6), 149.2 (C2), 148.4 (C12), 143.9 (C7), 138.0 (C4), 137.0 (C11), 129.3 (C9), 128.8 (C8), 127.7 (C10), 123.5 (C3), 122.0 (C5), 32.8 (C18), 29.3 (C19).

^{15}N-NMR (41 MHz, DCM-d_2): δ (ppm) = 300 (N1).

EM-MS-ESI (MeOH, $C_{17}H_{17}N_4ClHg$, m/z): calcd. for $[C_{17}H_{17}N_4ClHg+H]^+$ = 515.09128, found. [**[HLHgCl]**+H]$^+$ = 515.08944.

6 Synthesis of [LAuCl]

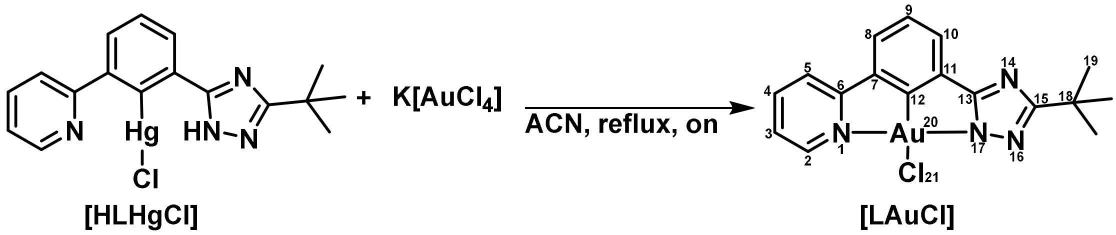

$K[AuCl_4]$ (53.3 mg, 0.14 mmol, 1.0 eq.) was added to a solution of **[HLHgCl]** (72.2 mg, 0.14 mmol, 1.0 eq.) in ACN (10 mL, dry). After refluxing overnight, the suspension was cooled to RT. The resulting precipitate was separated and washed with Et_2O. **[LAuCl]** was obtained as slightly yellow solid (49.9 mg,0.098 mmol, 70%).

^{1}H NMR (400 MHz, DMSO-d_6): δ (ppm) = 9.15 (d, br., 1H, H2), 8.48 (td, $^{3}J_{HH}$ = 7.7 Hz, $^{4}J_{HH}$ = 1.5 Hz, 1H, H4), 8.42 (d, $^{3}J_{HH}$ = 7.7 Hz, 1H, H5), 7.85 (ddd, $^{3}J_{HH}$ = 7.6, 5.6 Hz, $^{4}J_{HH}$ = 1.5 Hz, 1H, H3), 7.85 (overlap. 1H, H8), 7.66 (d, $^{3}J_{HH}$ = 7.7 Hz, 1H, H10), 7.59 (t, $^{3}J_{HH}$ = 7.7 Hz, 1H, H9), 1.40 (s, 9H, H19).

^{13}C NMR (101 MHz, DMSO-d_6): δ (ppm) = 166.7 (C12), 166.5 (C15), 165.9 (C13), 164.4 (C6), 149.8 (C2), 144.4 (C4), 140.1 (C7), 130.3 (C9), 127.8 (C11), 126.2 (C3), 125.3 (C10), 125.2 (C8), 123.1 (C5), 32.9 (C18), 29.2 (C19).

EM-MS-ESI (MeOH, $C_{17}H_{16}N_4AuCl$, *m/z*): calcd. for $[C_{17}H_{16}N_4AuCl+H]^+$ = 509.08018, found. [**[LAuCl]**+H]$^+$ = 825.08276.

NMR spectroscopy and mass spectrometry

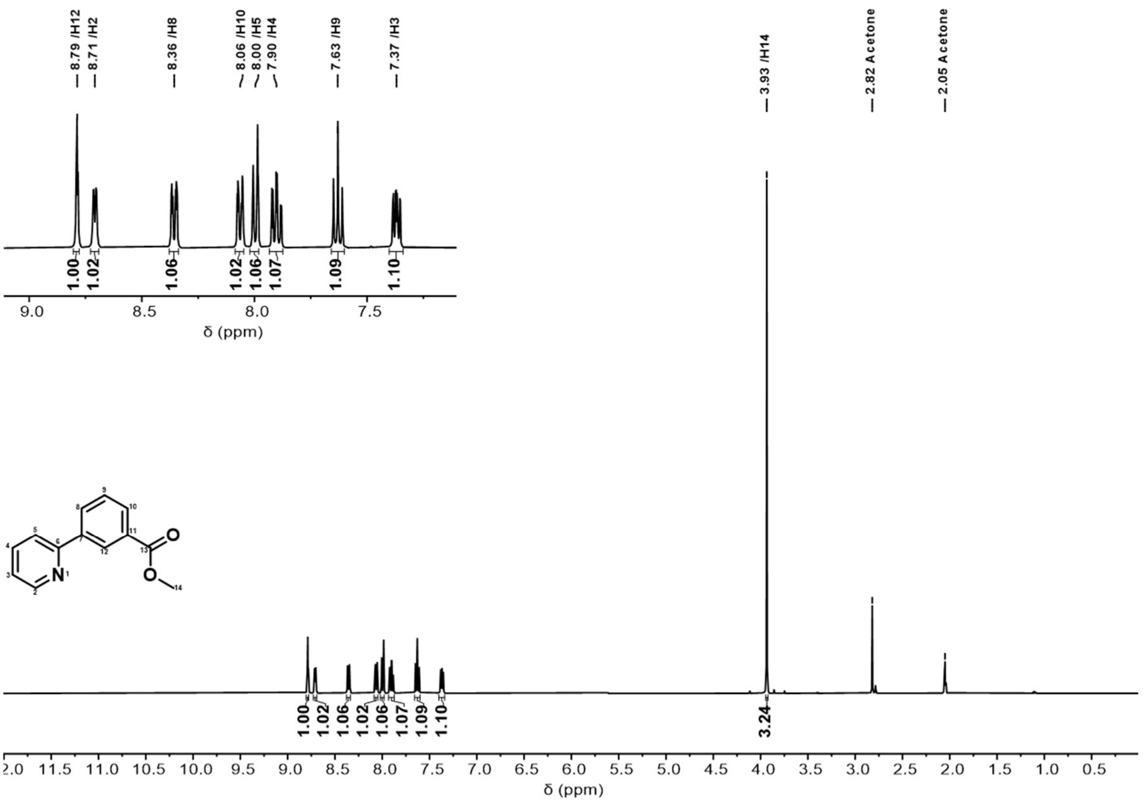

Figure S1. ^{1}H-NMR spectrum (400 MHz, acetone-d_6) of **1**.

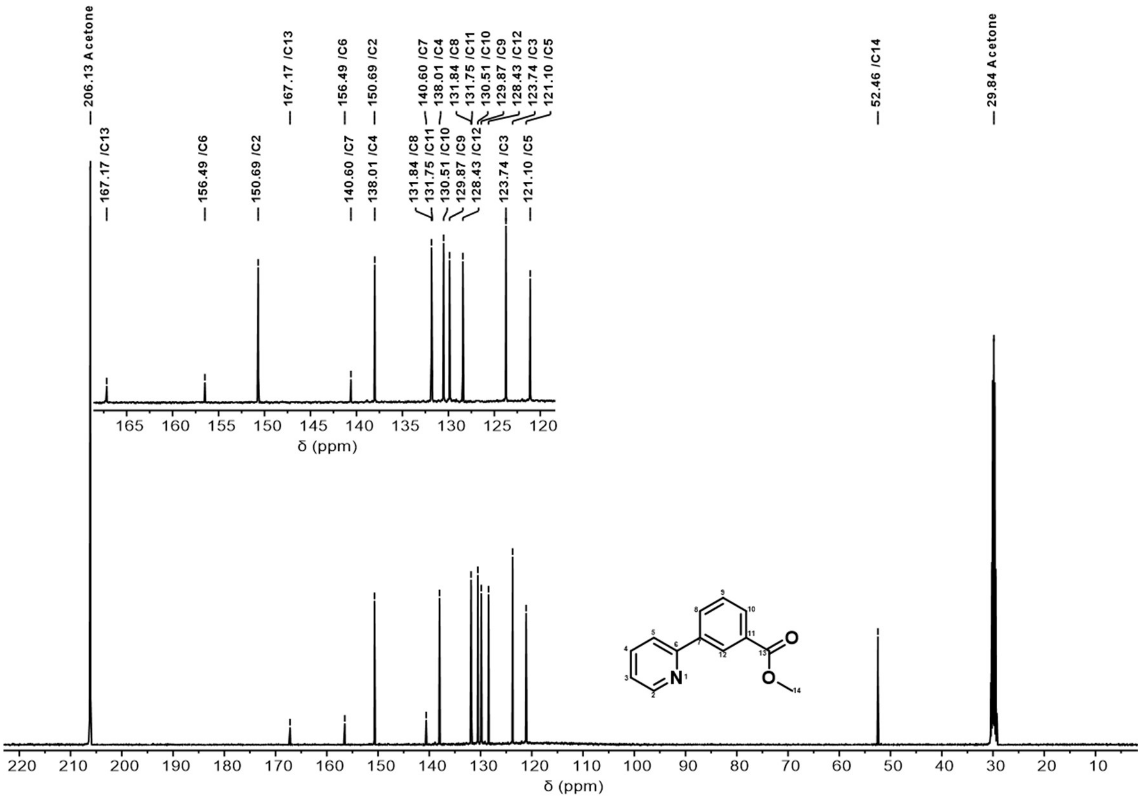

Figure S2. ^{13}C-NMR spectrum (101 MHz, acetone-d_6) of **1**.

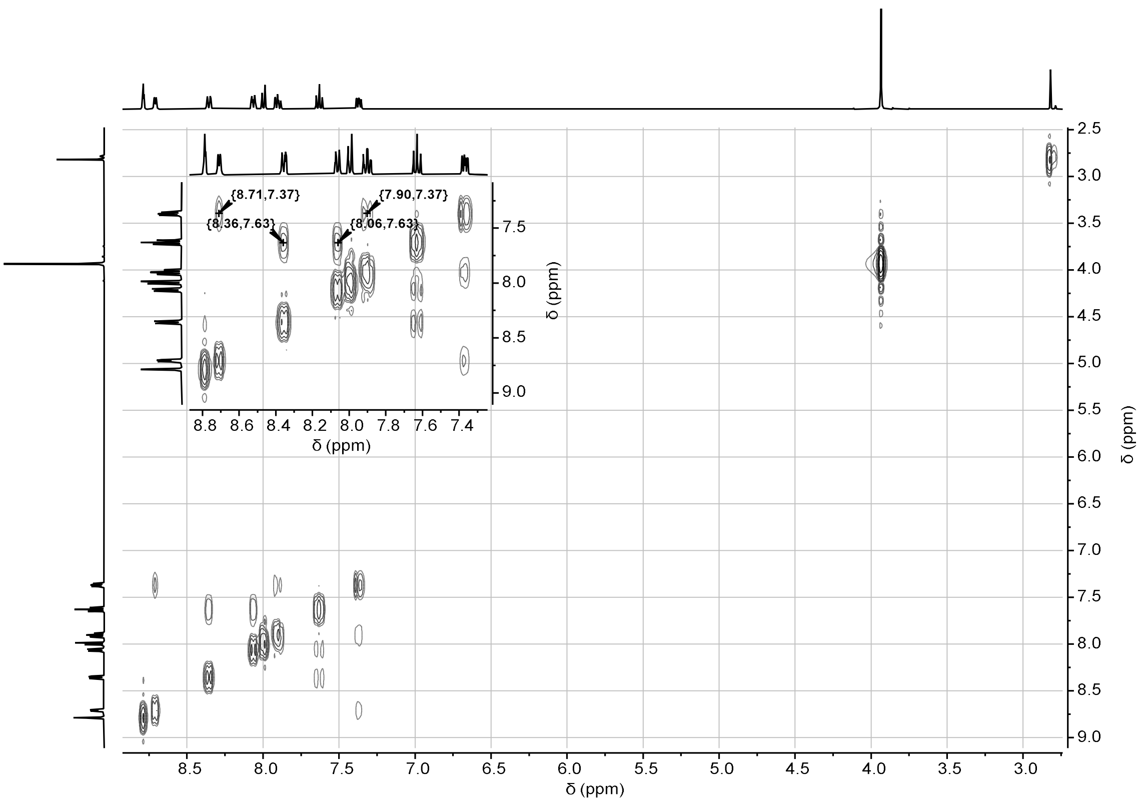

Figure S3. $^{1}H,^{1}H$-COSY-NMR spectrum (400 MHz, acetone-d_6) of **1**.

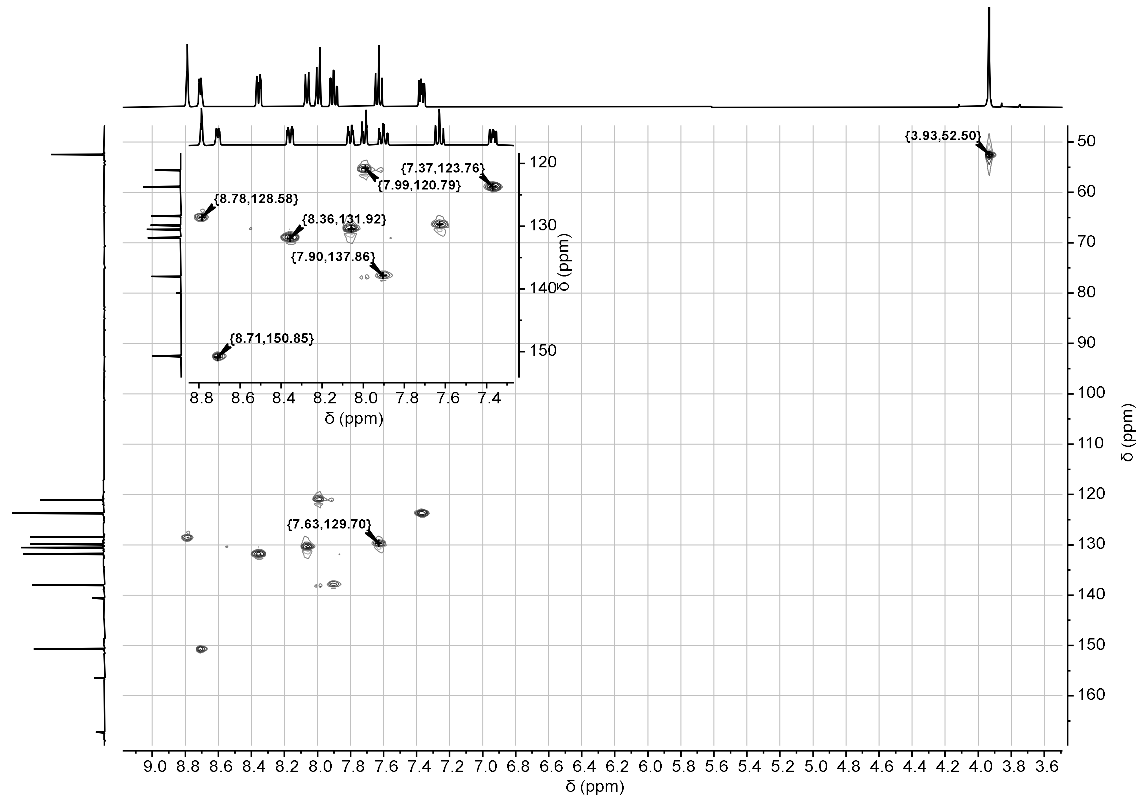

Figure S4. $^{1}H,^{13}C$-HSQC-NMR spectrum (400 MHz, 101 MHz, acetone-d_6) of **1**.

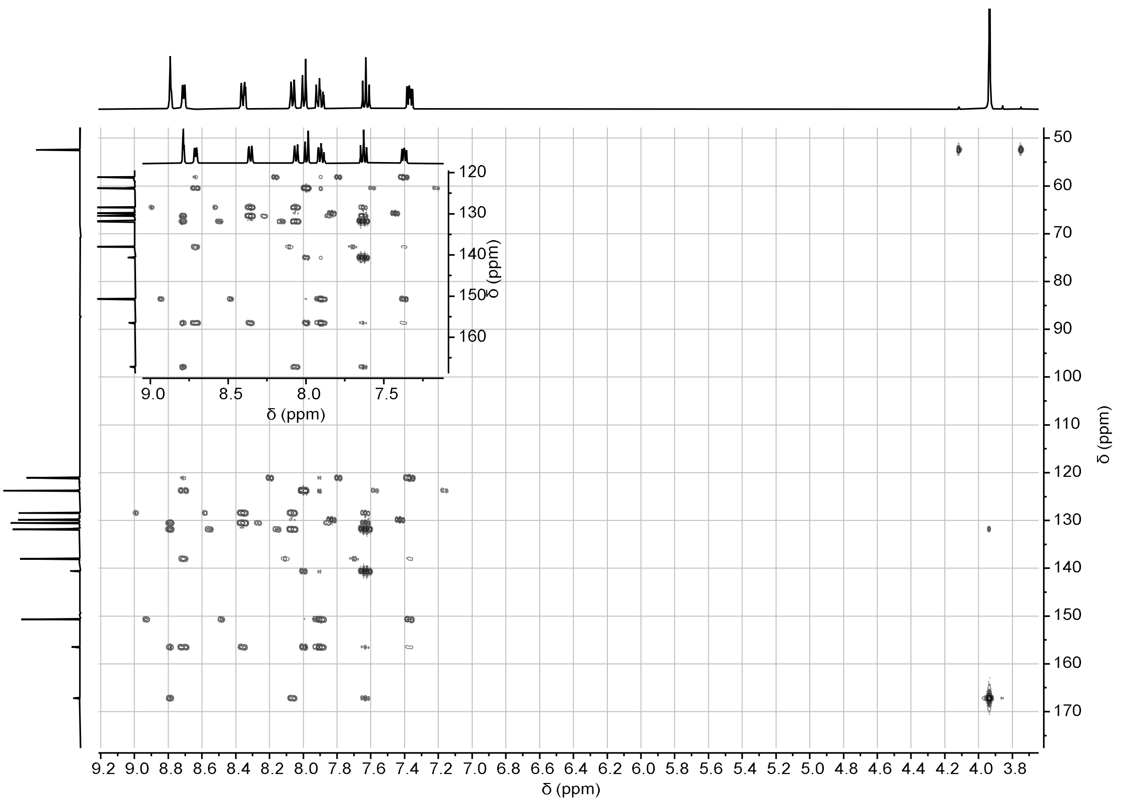

Figure S5. $^{1}H,^{13}C$-HMBC-NMR spectrum (400 MHz, 101 MHz*,* acetone-d_6) of **1**.

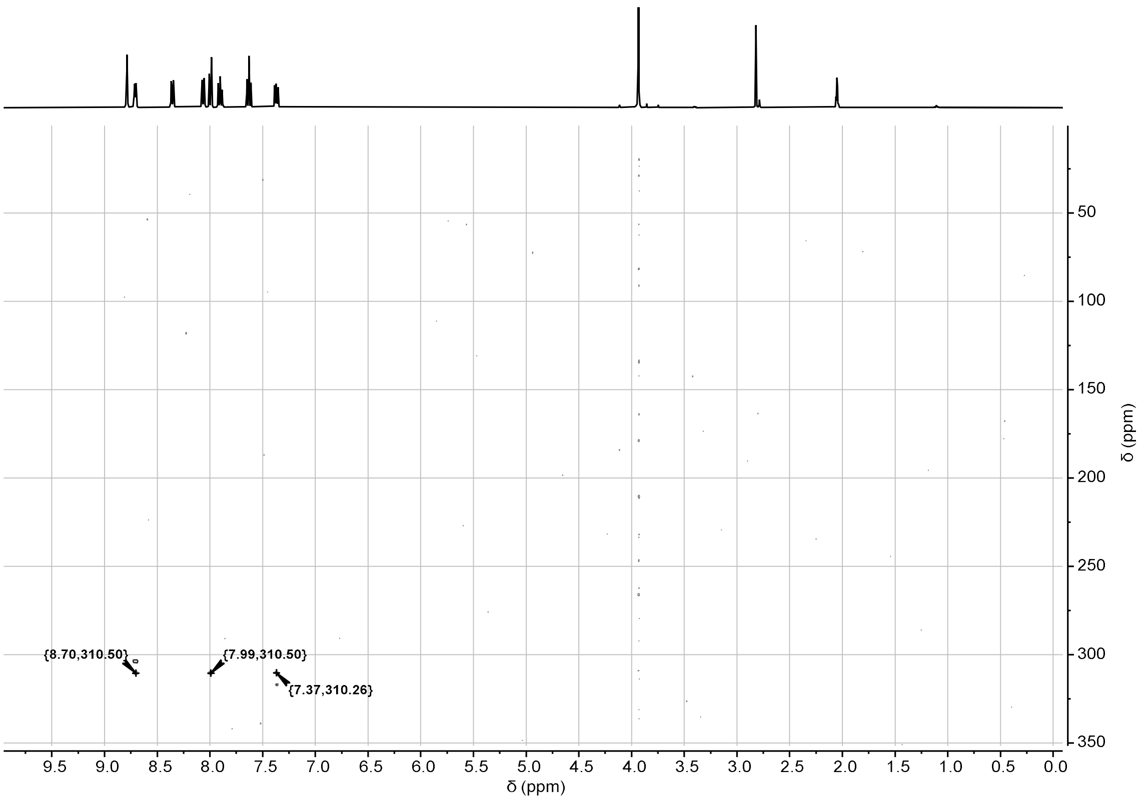

Figure S6. $^{1}H,^{15}N$-HMBC-NMR spectrum (400 MHz, 41 MHz*,* acetone-d_6) of **1**.

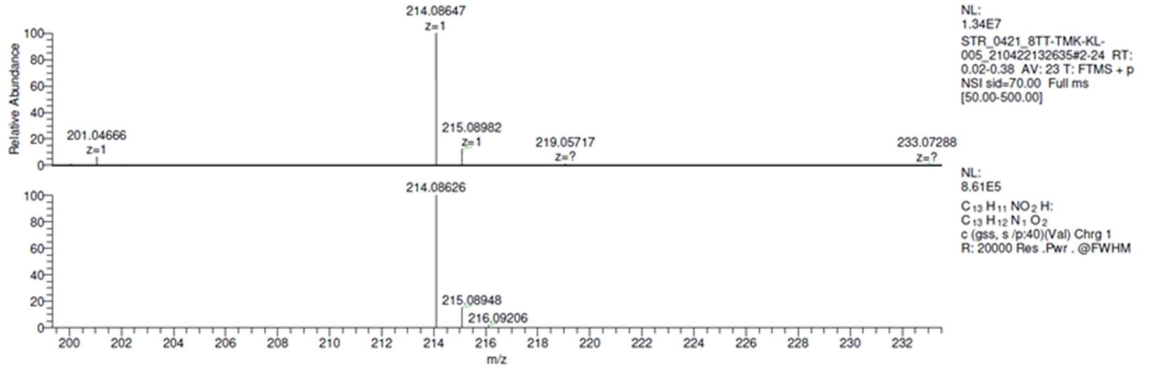

Figure S7. Mass spectrum of **1** (top). Additional simulation of the [**1**+H]$^{+}$ adduct (bottom).

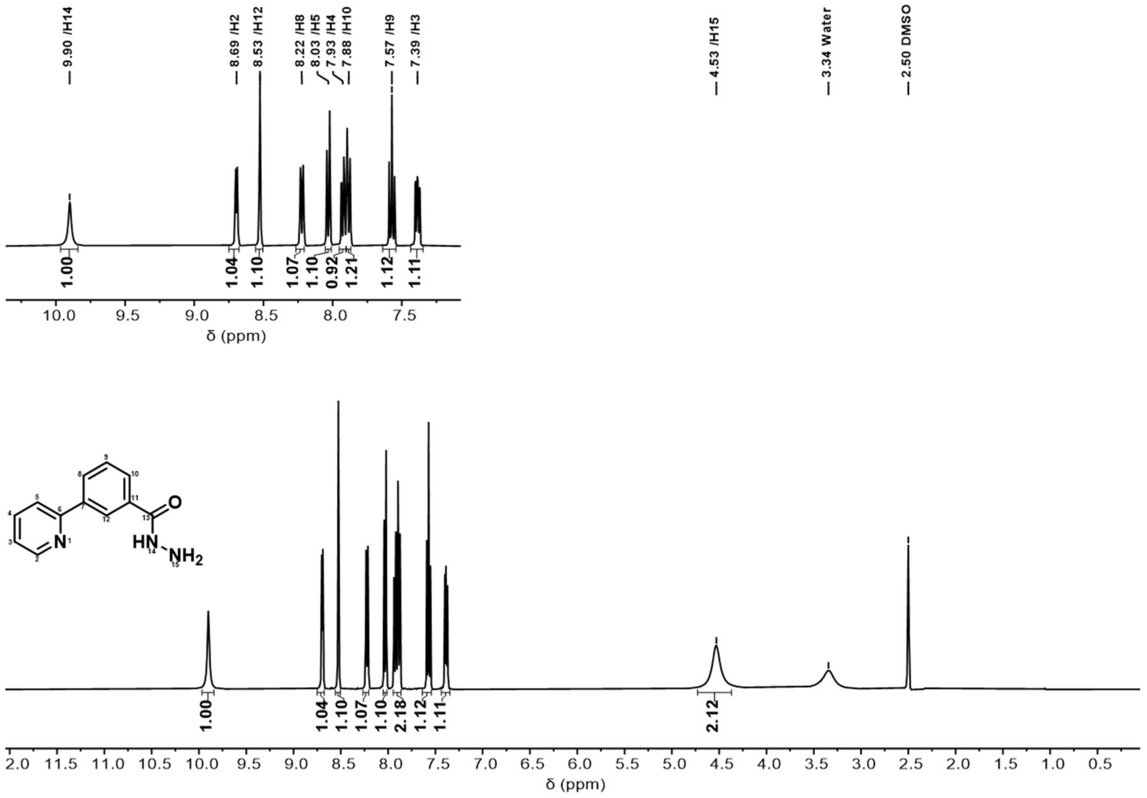

Figure S8. ^{1}H-NMR spectrum (400 MHz, DMSO-d_6) of **2**.

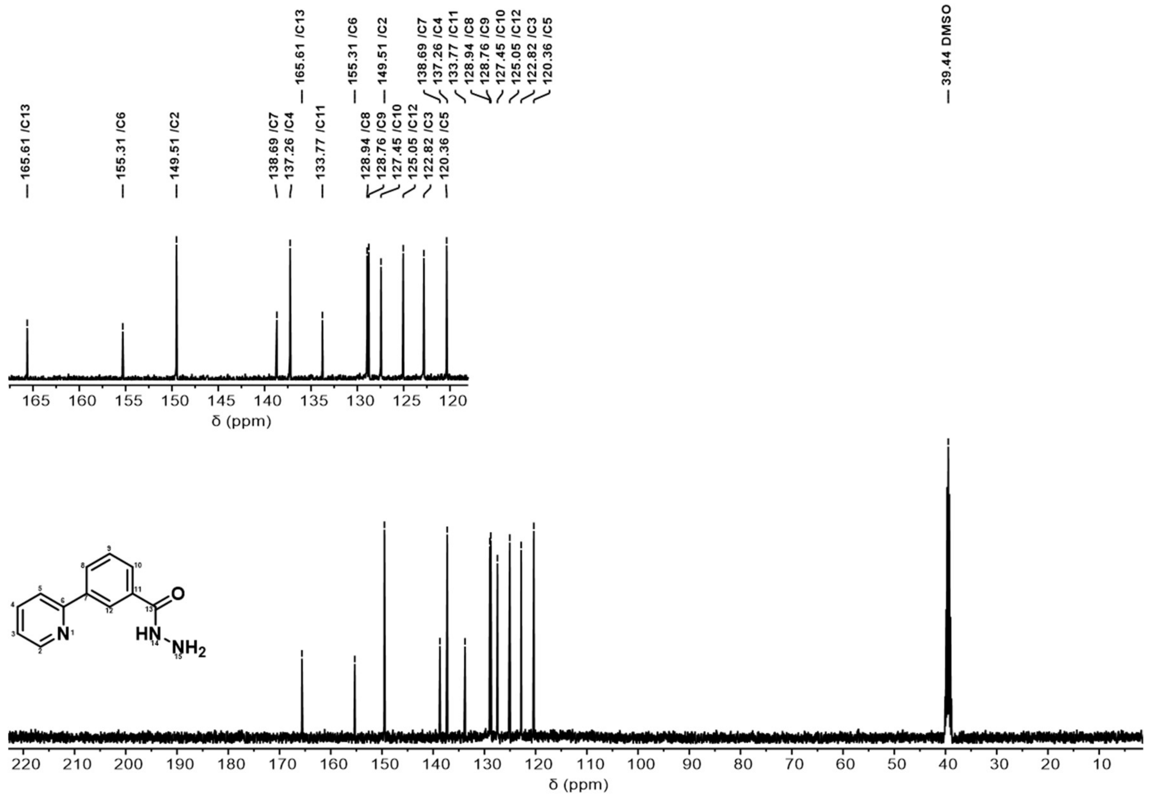

Figure S9. ^{13}C-NMR spectrum (101 MHz, DMSO-d_6) of **2**.

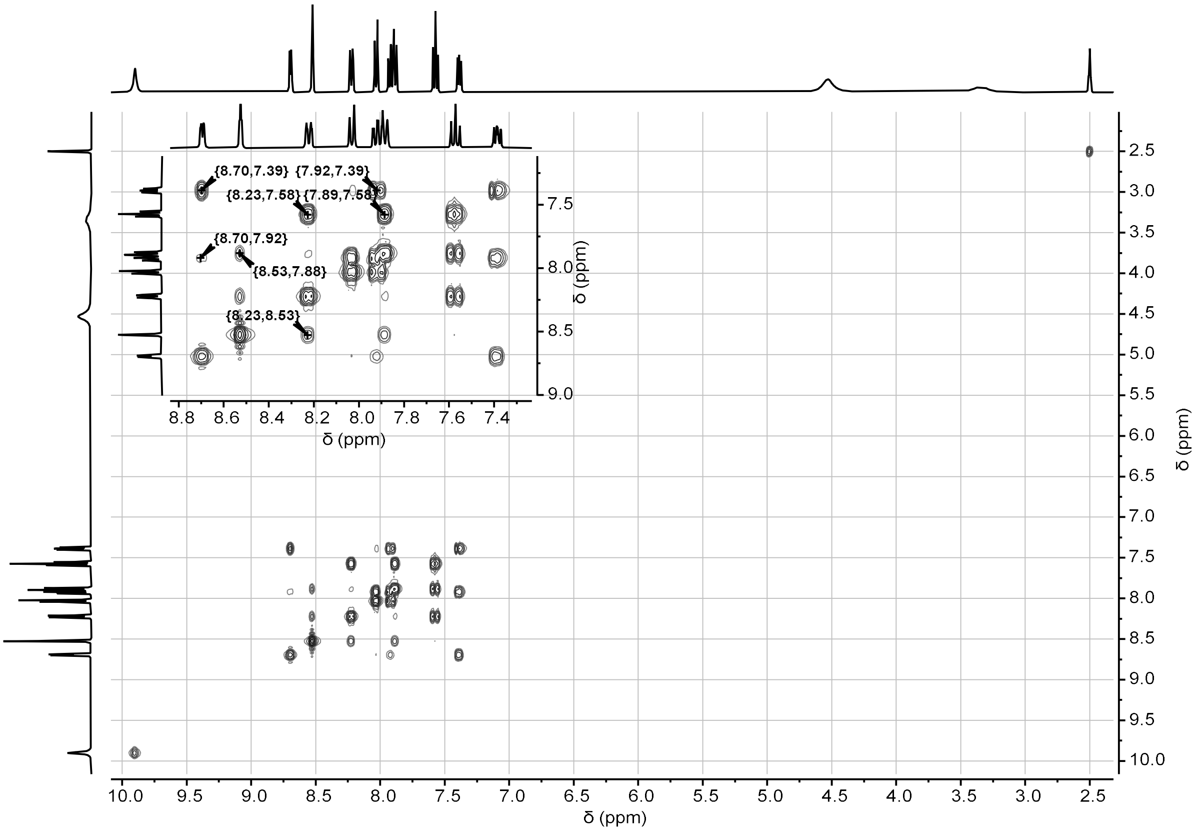

Figure S10. ^{1}H,^{1}H-COSY-NMR spectrum (400 MHz, DMSO-d_6) of **2**.

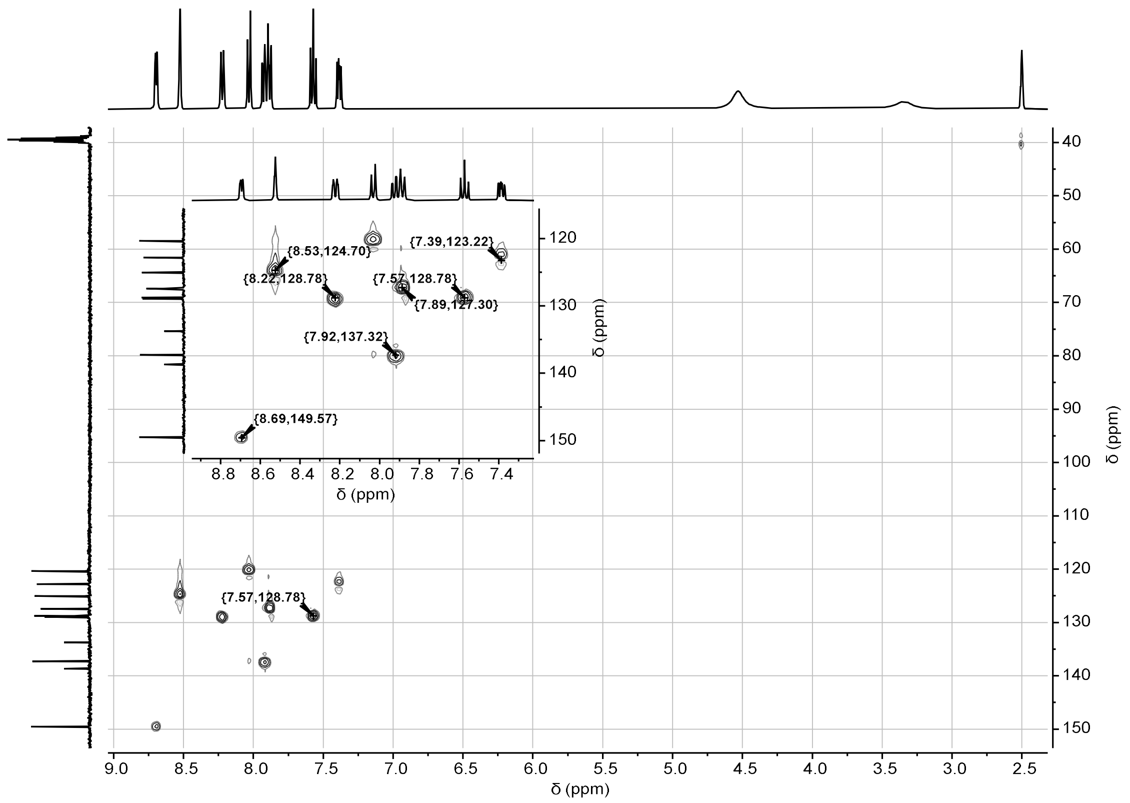

Figure S11. ^{1}H,^{13}C-HSQC-NMR spectrum (400 MHz, 101 MHz, DMSO-d_6) of **2**.

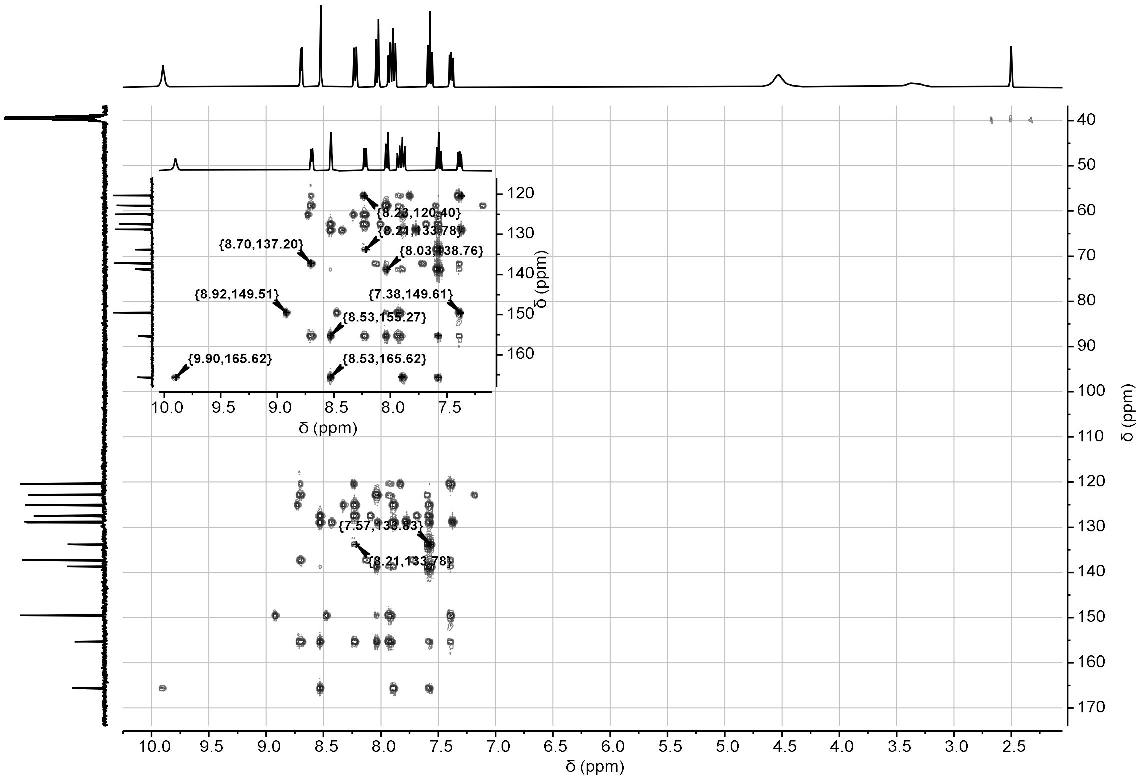

Figure S12. ^{1}H,^{13}C-HMBC-NMR spectrum (400 MHz, 101 MHz, DMSO-d_6) of **2**.

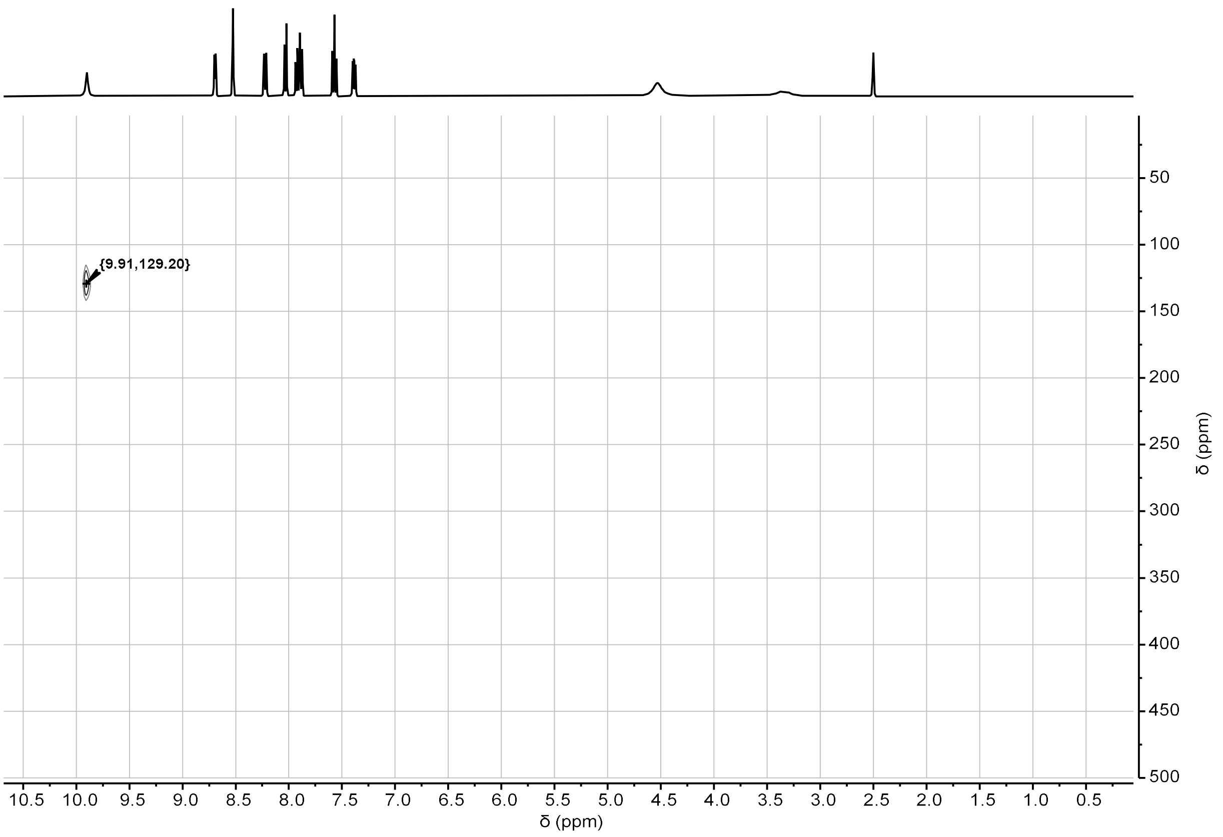

Figure S13. $^{1}H,^{15}N$-HSQC-NMR spectrum (400 MHz, 41 MHz, DMSO-d_6) of **2**.

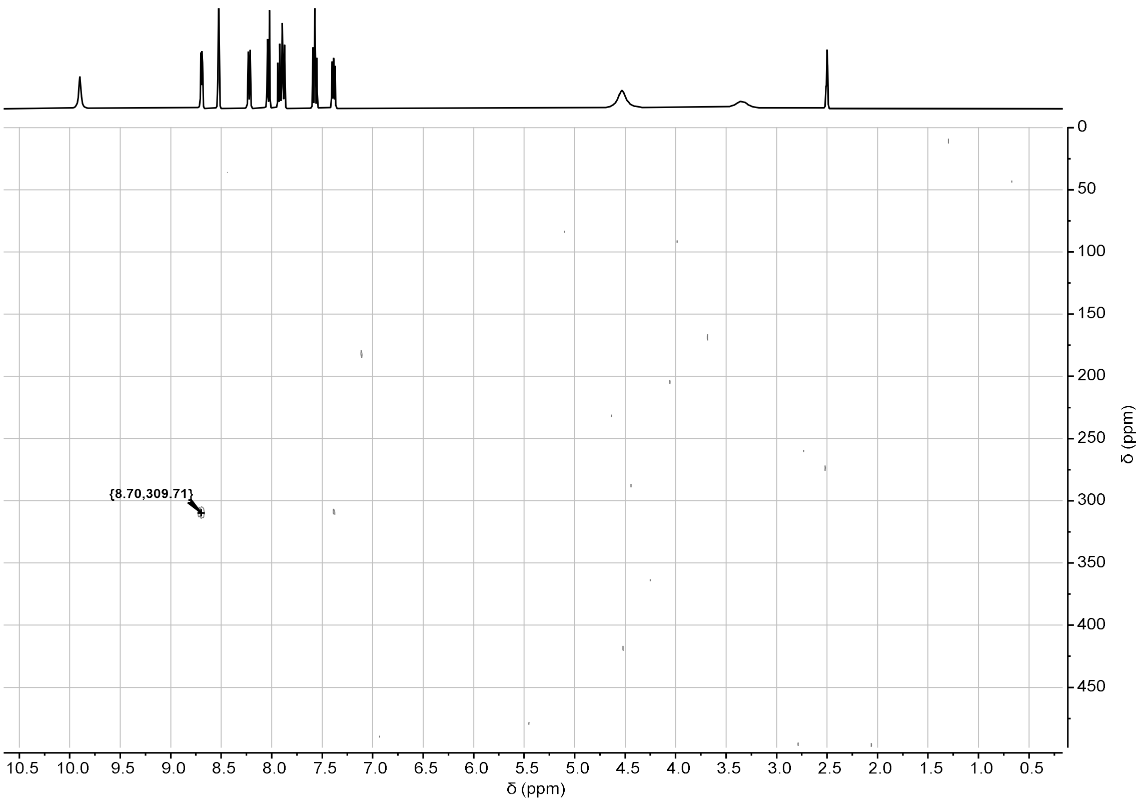

Figure S14. $^{1}H,^{15}N$-HMBC-NMR spectrum (400 MHz, 41 MHz, DMSO-d_6) of **2**.

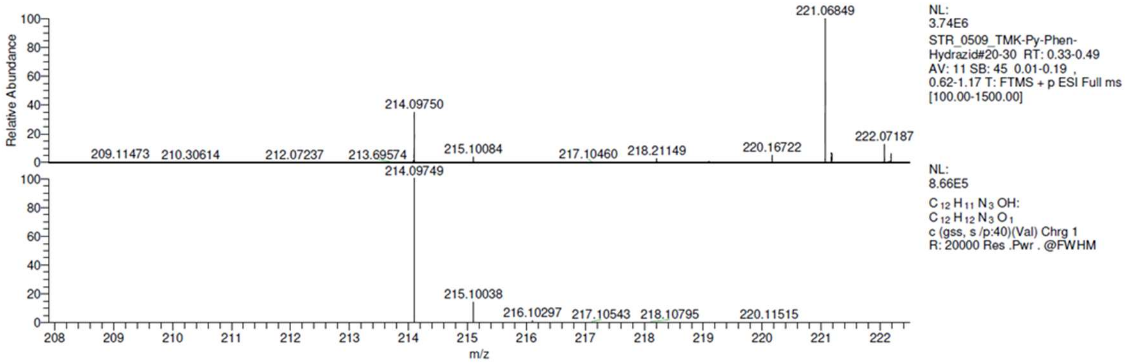

Figure S15. Mass spectrum **2** (top). Additional simulation of the [**2**+H]$^+$ adduct (bottom).

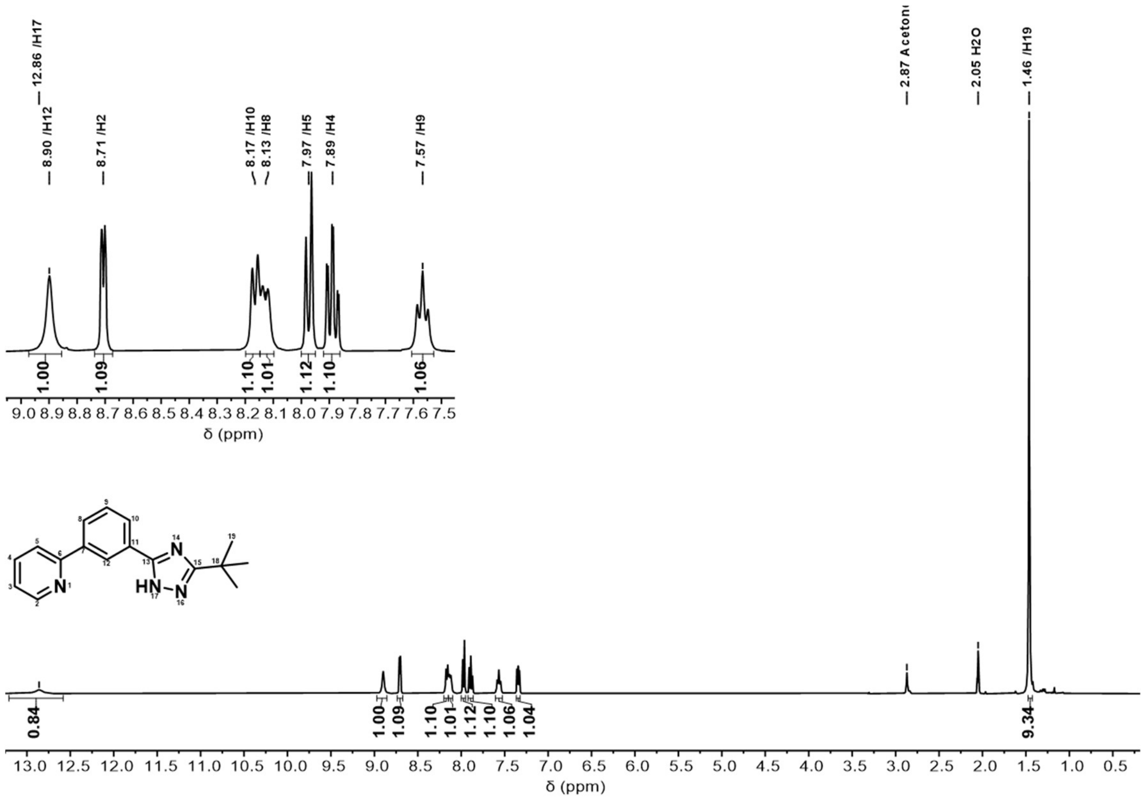

Figure S16. ^{1}H-NMR spectrum (400 MHz, acetone-d_6) of $\mathbf{H_2L}$.

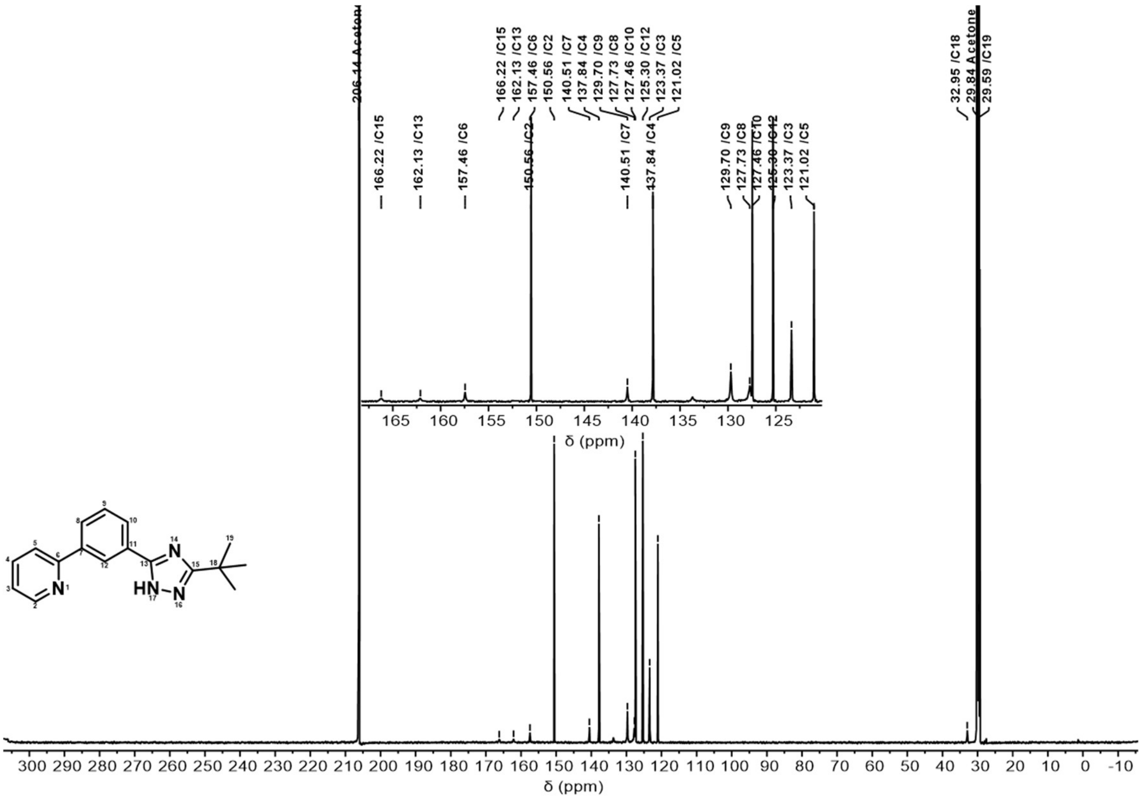

Figure S17. ^{13}C-NMR spectrum (101 MHz, acetone-d_6) of **H_2L**.

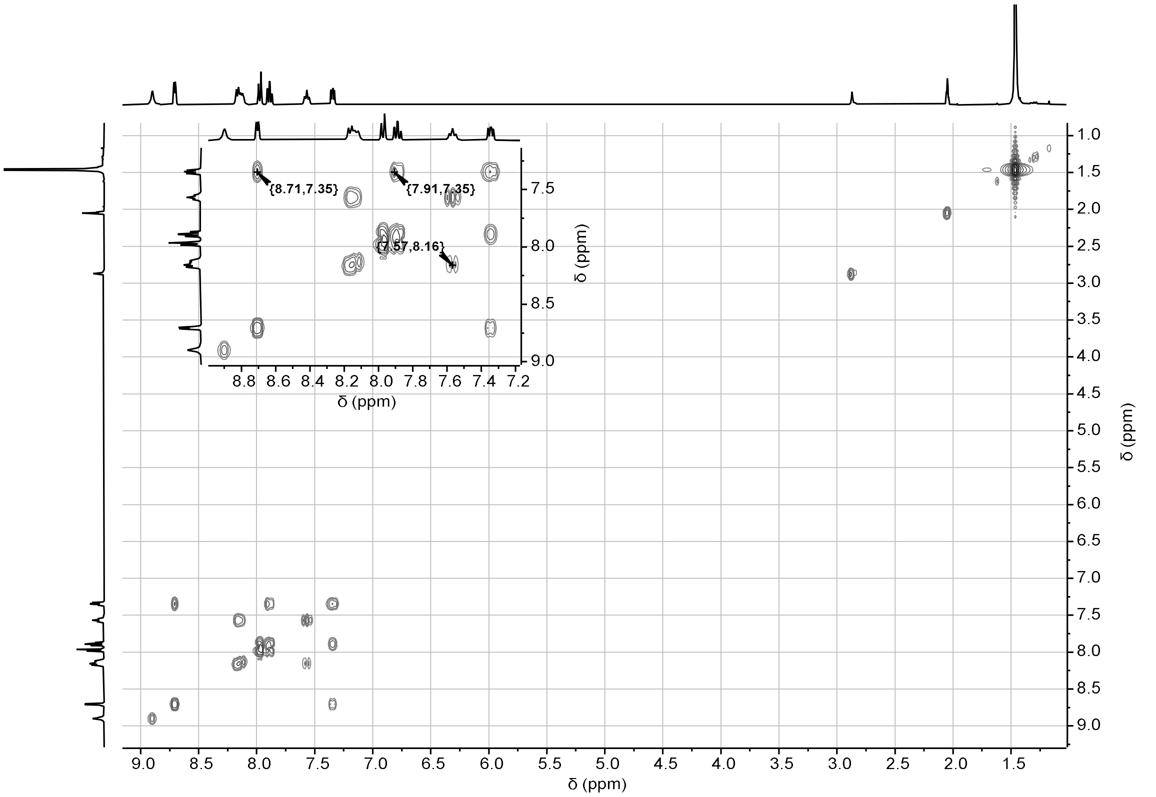

Figure S18. ^{1}H,^{1}H-COSY-NMR spectrum (400 MHz, acetone-d_6) of **H_2L**.

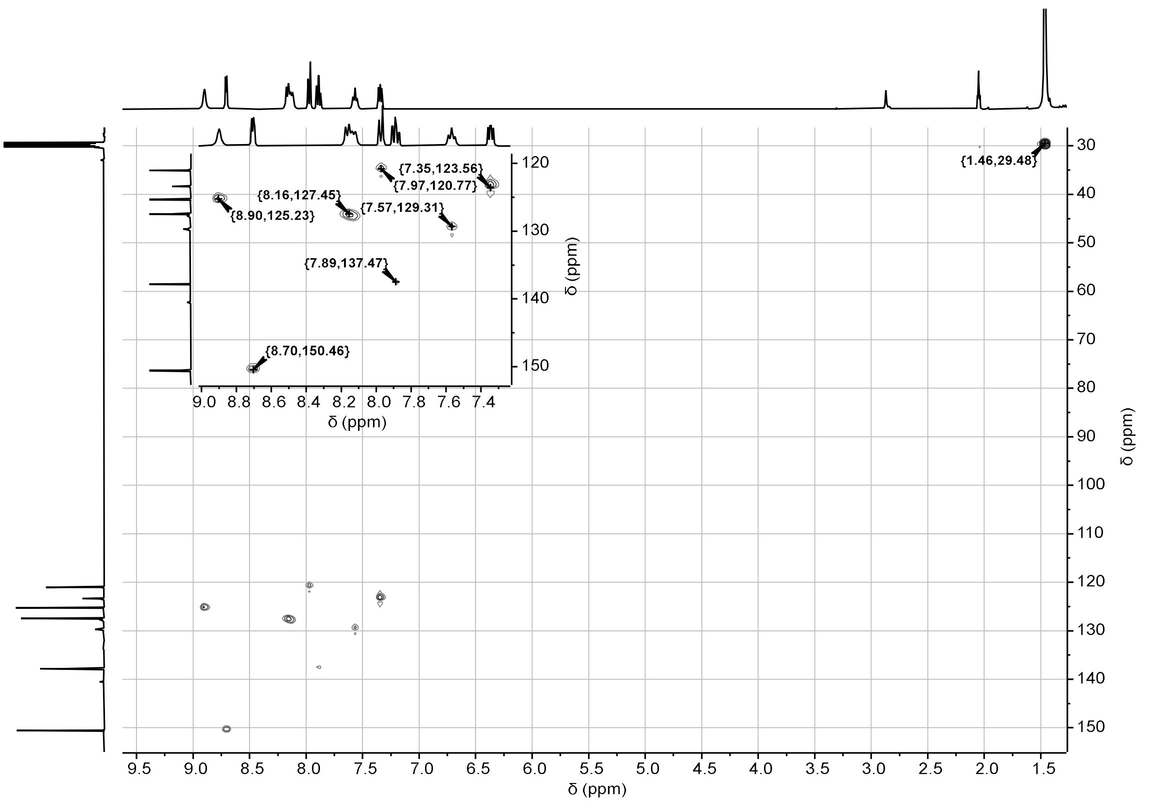

Figure S19. ^{1}H,^{13}C-HSQC-NMR spectrum (400 MHz, 101 MHz, acetone-d_6) of **H_2L**.

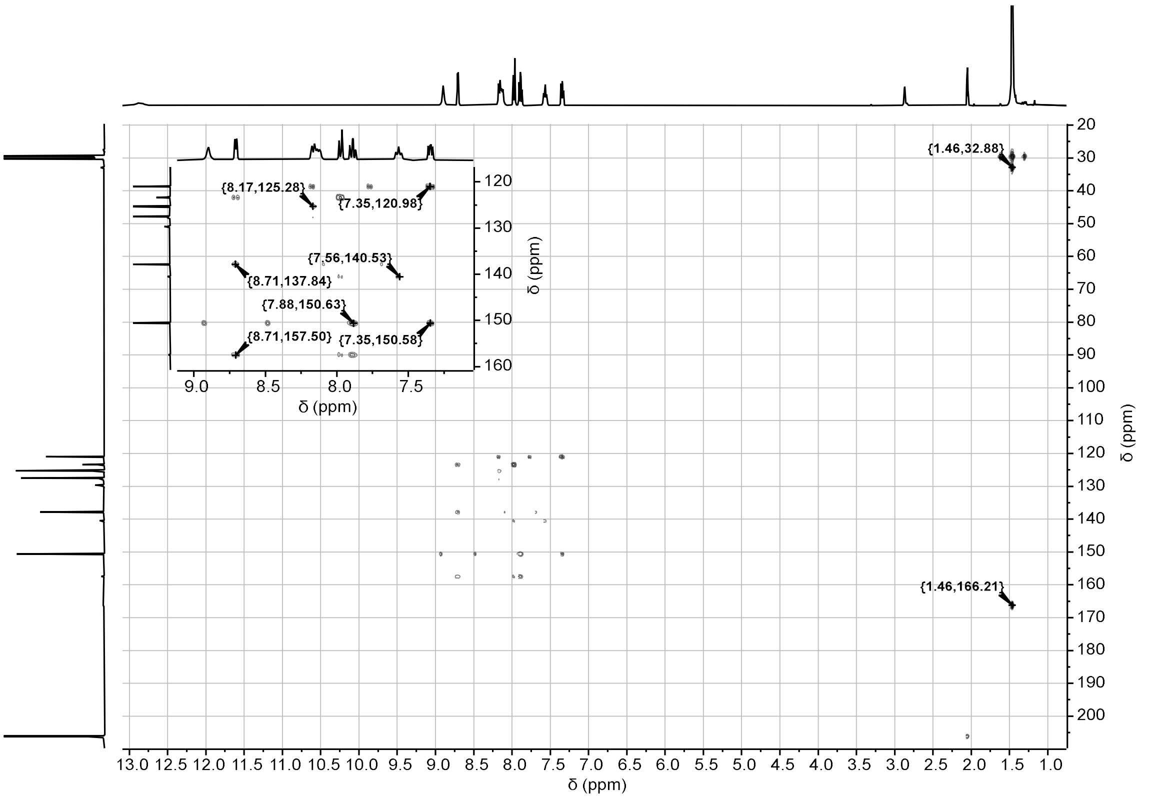

Figure S20. ^{1}H,^{13}C-HMBC-NMR spectrum (400 MHz, 101 MHz, acetone-d_6) of **H_2L**.

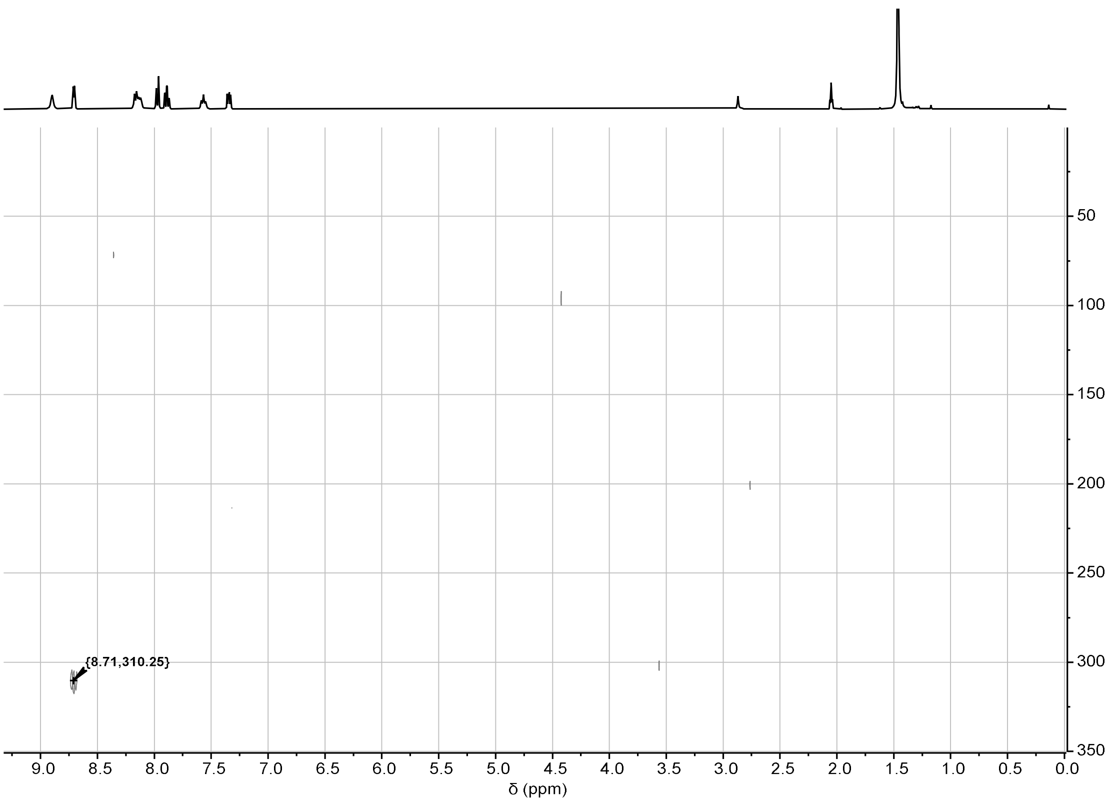

Figure S21. $^{1}H,^{15}N$-HMBC-NMR spectrum (400 MHz, 41 MHz*,* acetone-d_6) of $\mathbf{H_2L}$.

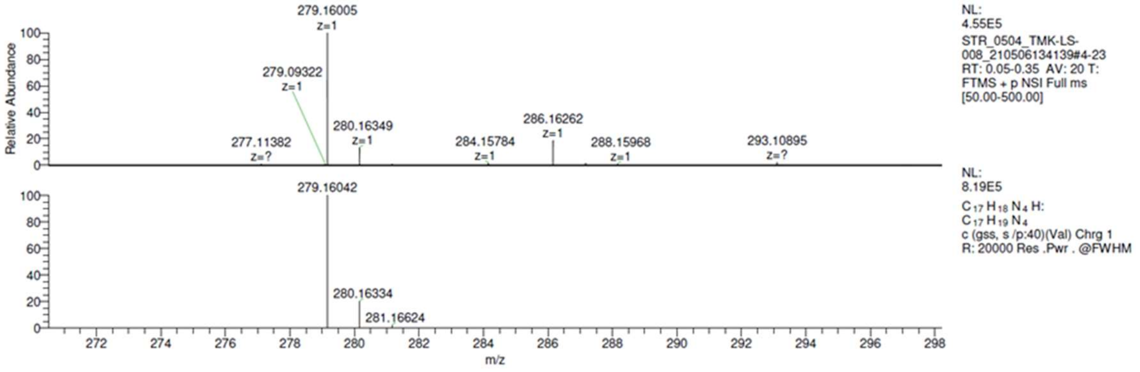

Figure S22. Mass spectrum of $\mathbf{H_2L}$ (top). Additional simulation of the $[\mathbf{H_2L}+H]^+$ adduct (bottom).

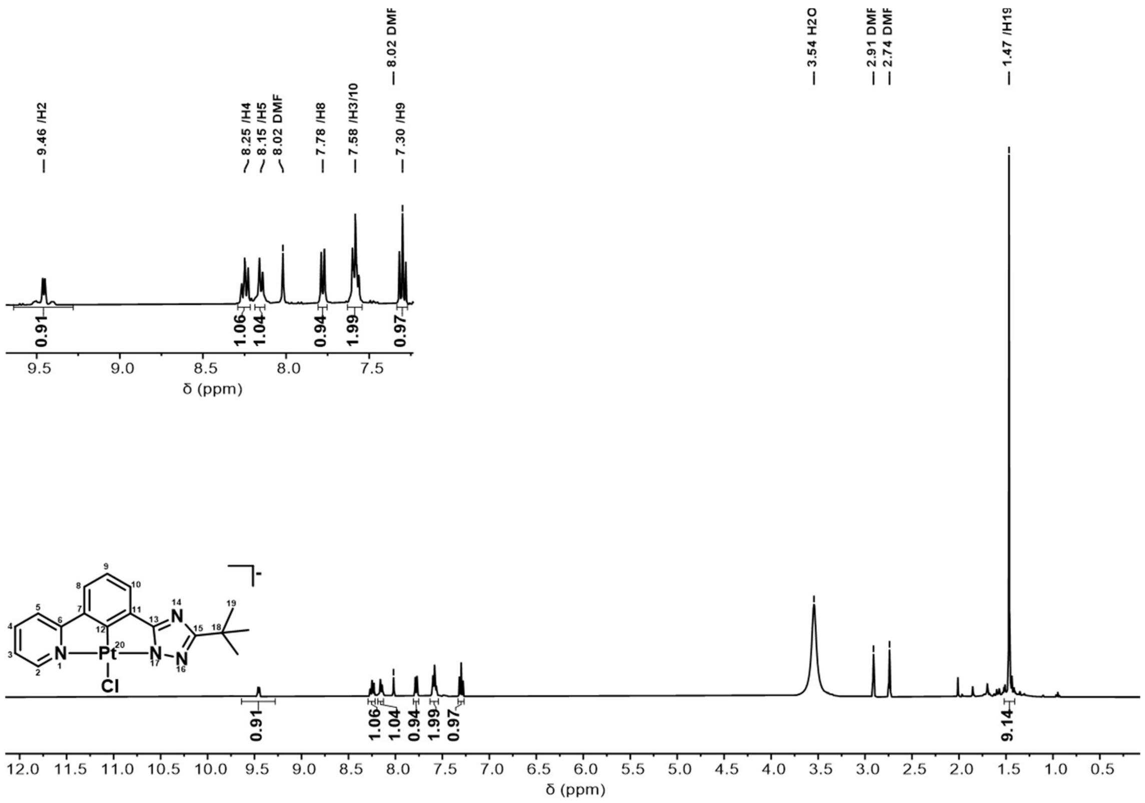

Figure S23. ^{1}H-NMR spectrum (400 MHz, DMF-d_7) of **[LPtCl]**.

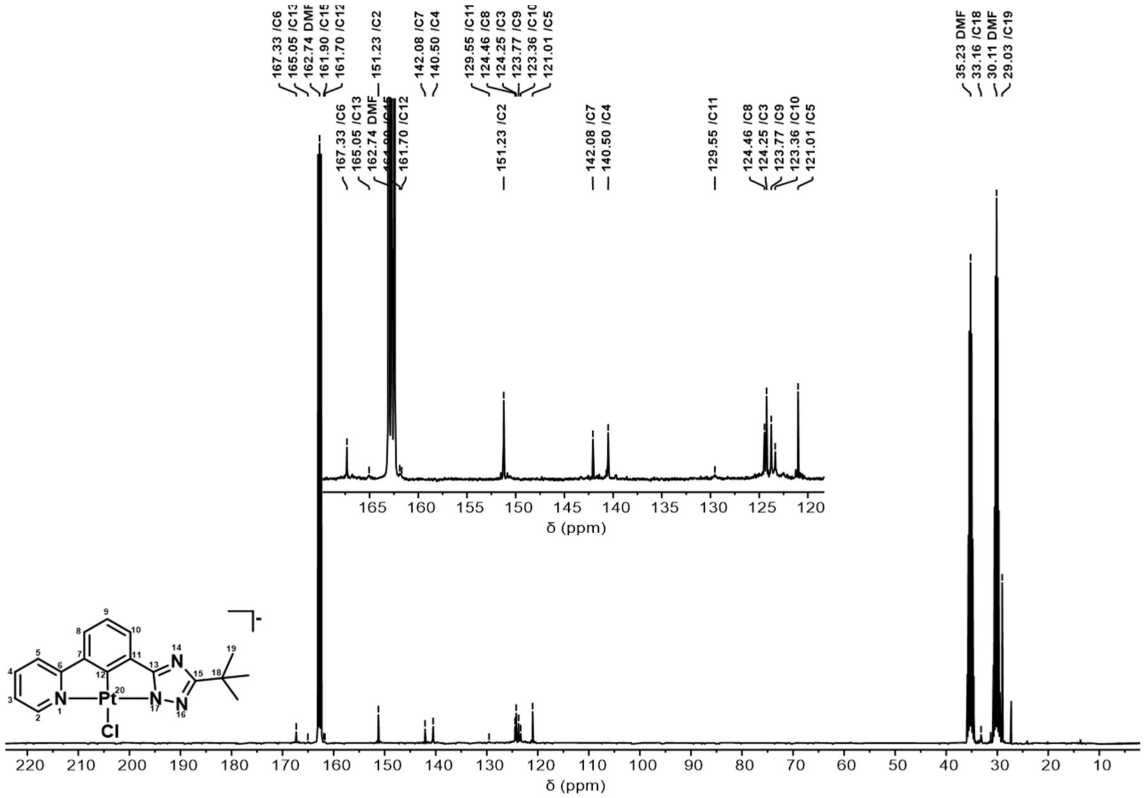

Figure S24. ^{13}C-NMR spectrum (101 MHz, DMF-d_7) of **[LPtCl]**.

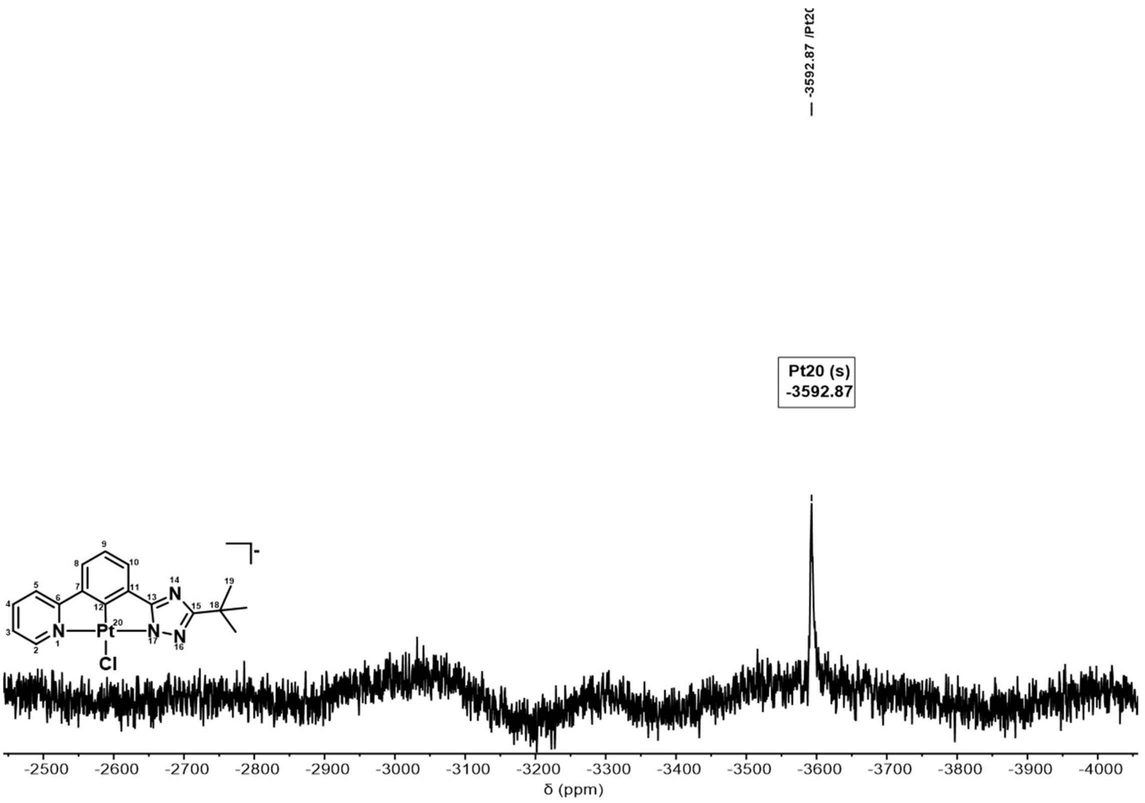

Figure S25. ^{195}Pt-NMR spectrum (86 MHz, DMF-d_7) of **[LPtCl]**.

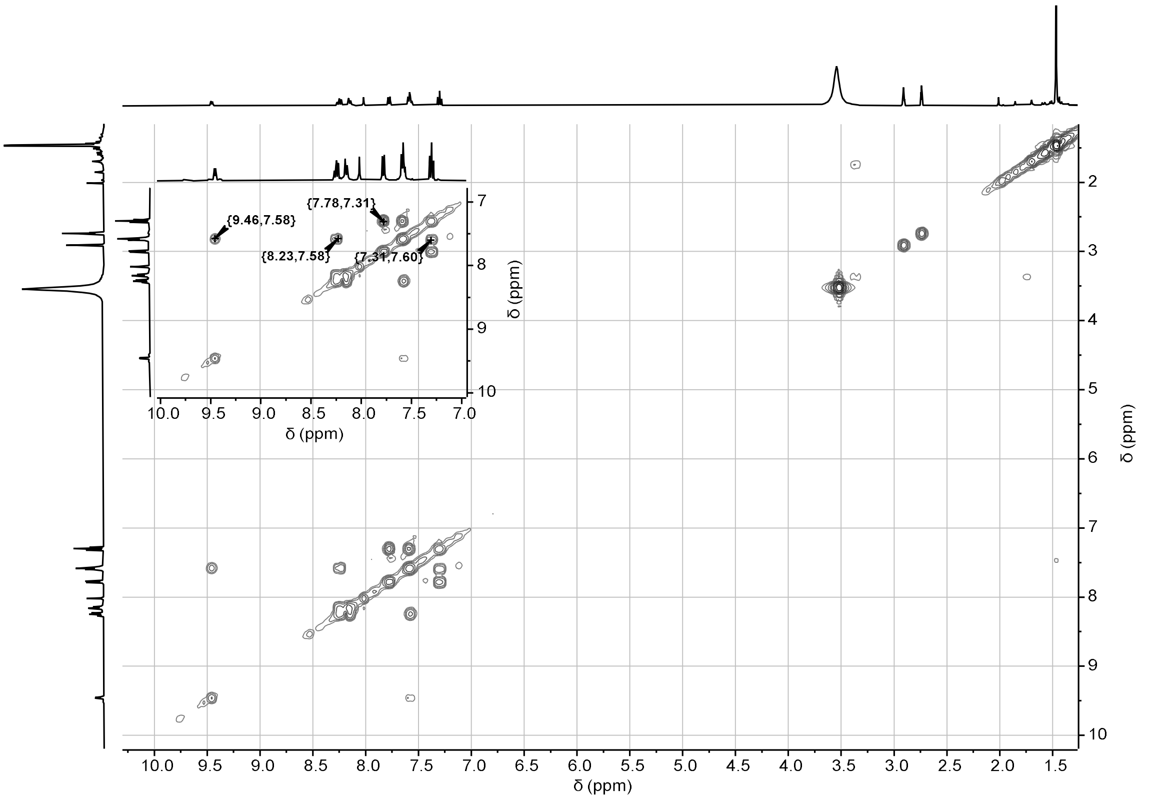

Figure S26. ^{1}H,^{1}H-COSY-NMR spectrum (400 MHz, DMF-d_7) of **[LPtCl]**.

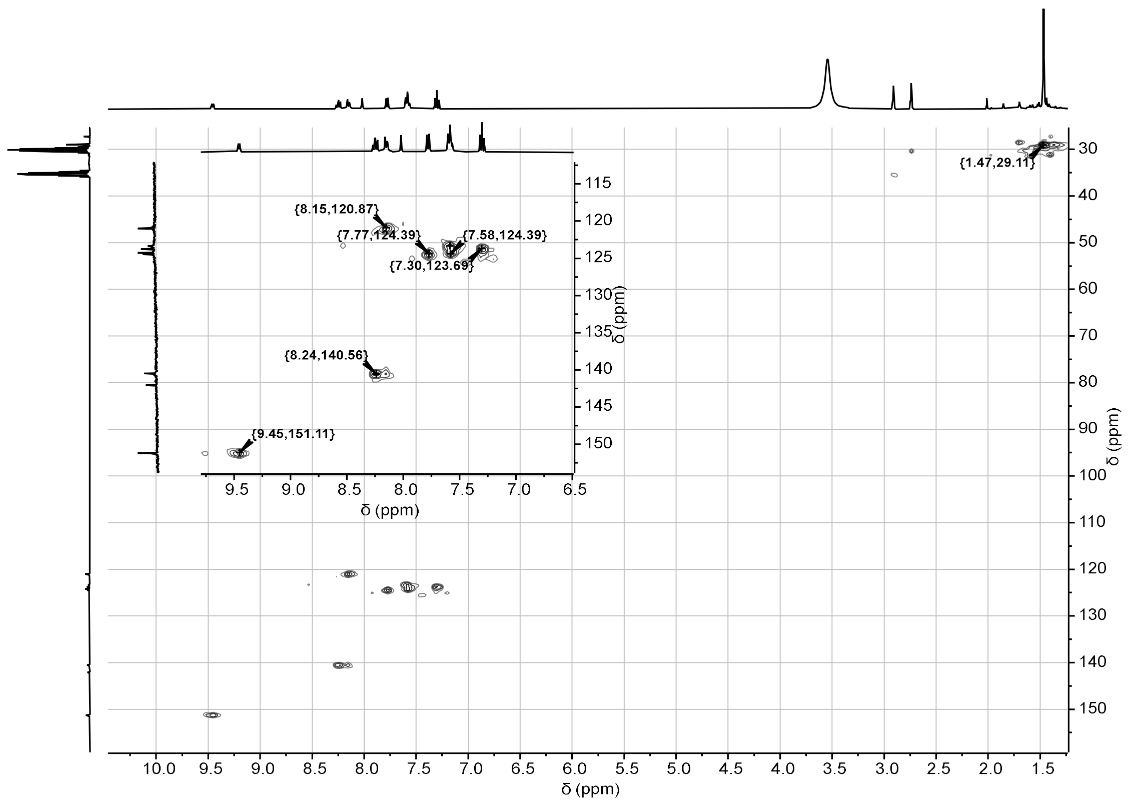

Figure S27. $^{1}H,^{13}C$-HSQC-NMR spectrum (400 MHz, 101 MHz, DMF-d_7) of **[LPtCl]**.

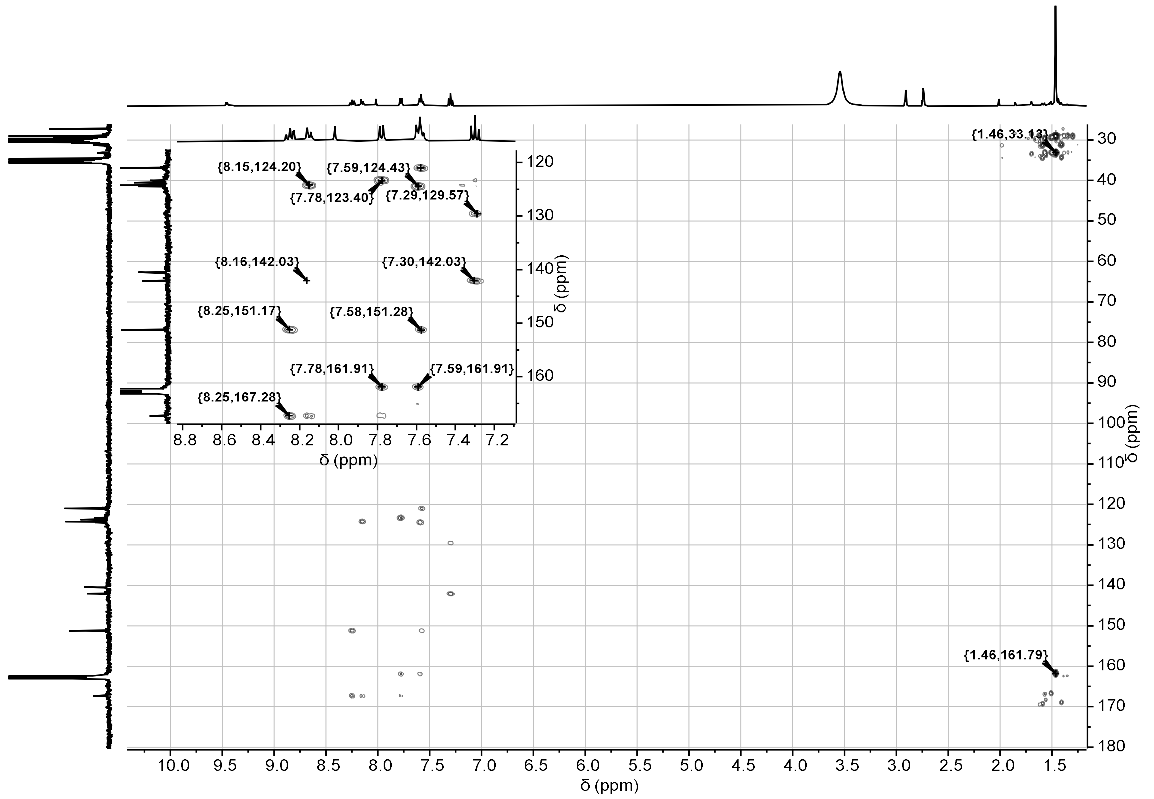

Figure S28. $^{1}H,^{13}C$-HMBC-NMR spectrum (400 MHz, 101 MHz, DMF-d_7) of **[LPtCl]**.

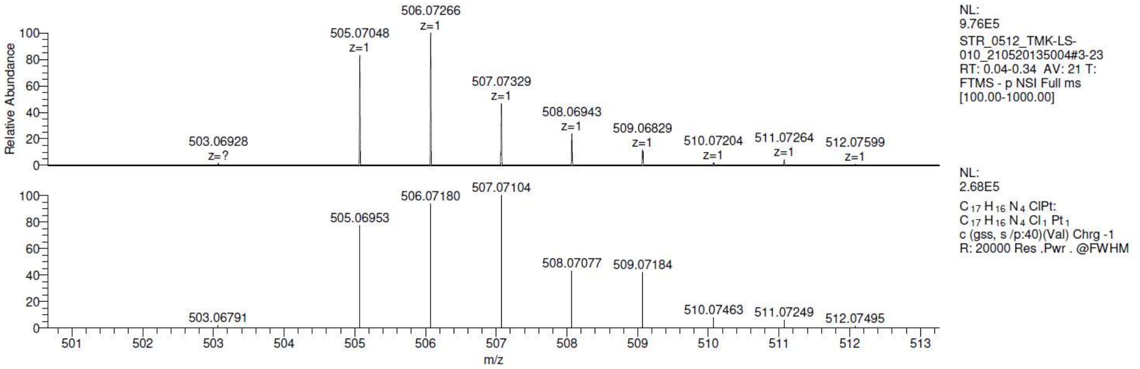

Figure S29. Mass spectrum of **[LPtCl]** (top). Additional simulation of the [**[LPtCl]**+H]$^+$ adduct (bottom).

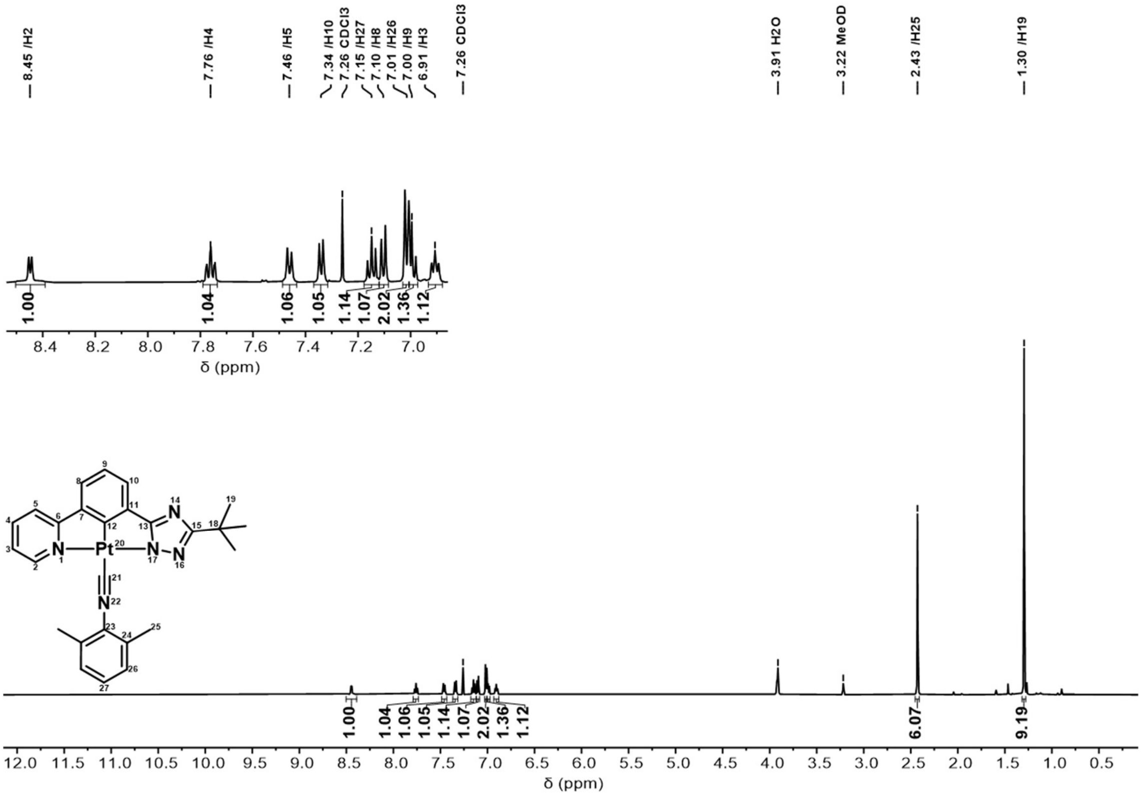

Figure S30. ^{1}H-NMR spectrum (500 MHz, $CDCl_3$/methanol-d_4) of **[LPtCNPhen]**.

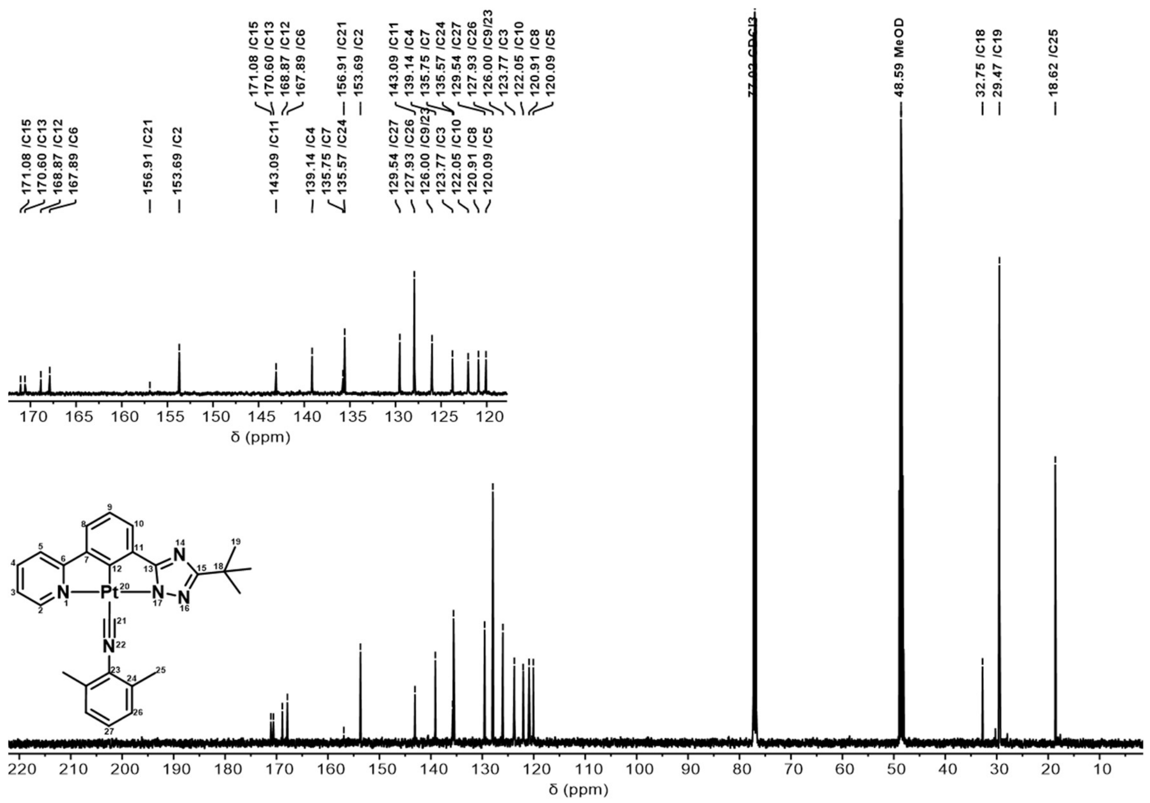

Figure S31. ^{13}C-NMR spectrum (126 MHz, $CDCl_3$/methanol-d_4) of **[LPtCNPhen]**.

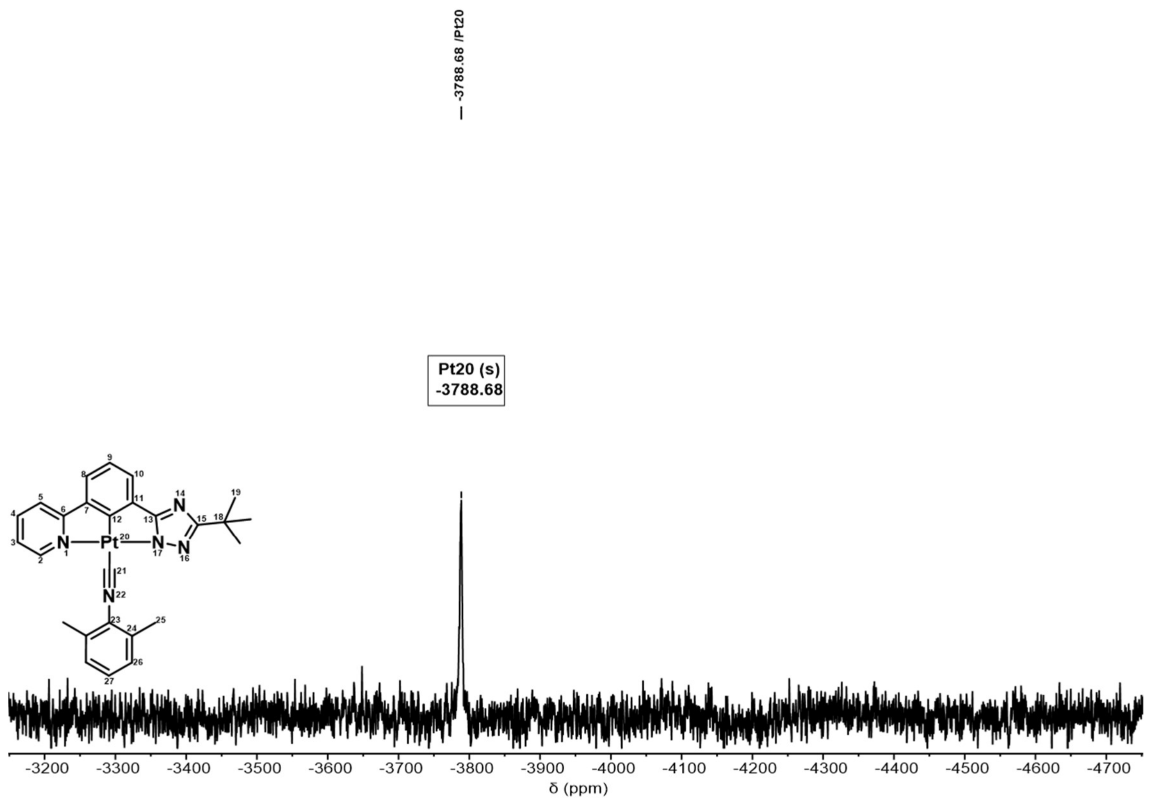

Figure S32. ^{195}Pt-NMR spectrum (107 MHz, $CDCl_3$/methanol-d_4) of **[LPtCNPhen]**.

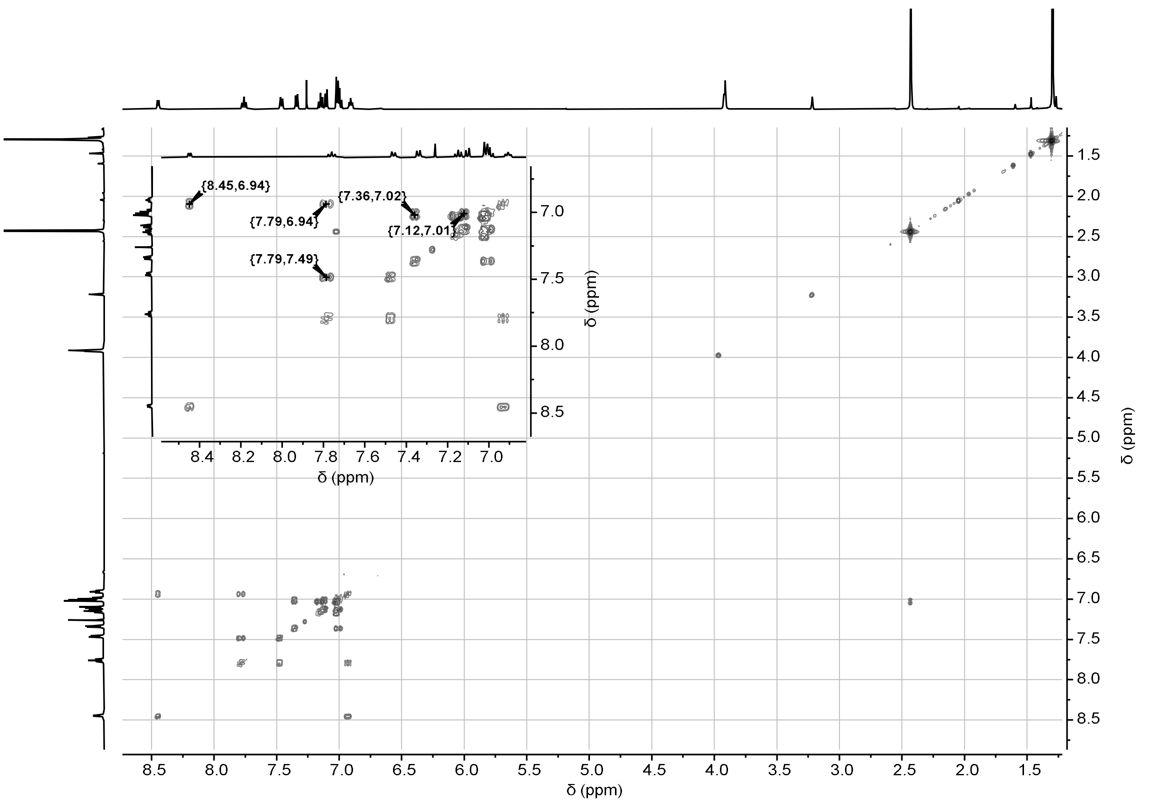

Figure S33. ^{1}H,^{1}H-COSY-NMR spectrum (500 MHz, $CDCl_3$/methanol-d_4) of **[LPtCNPhen]**.

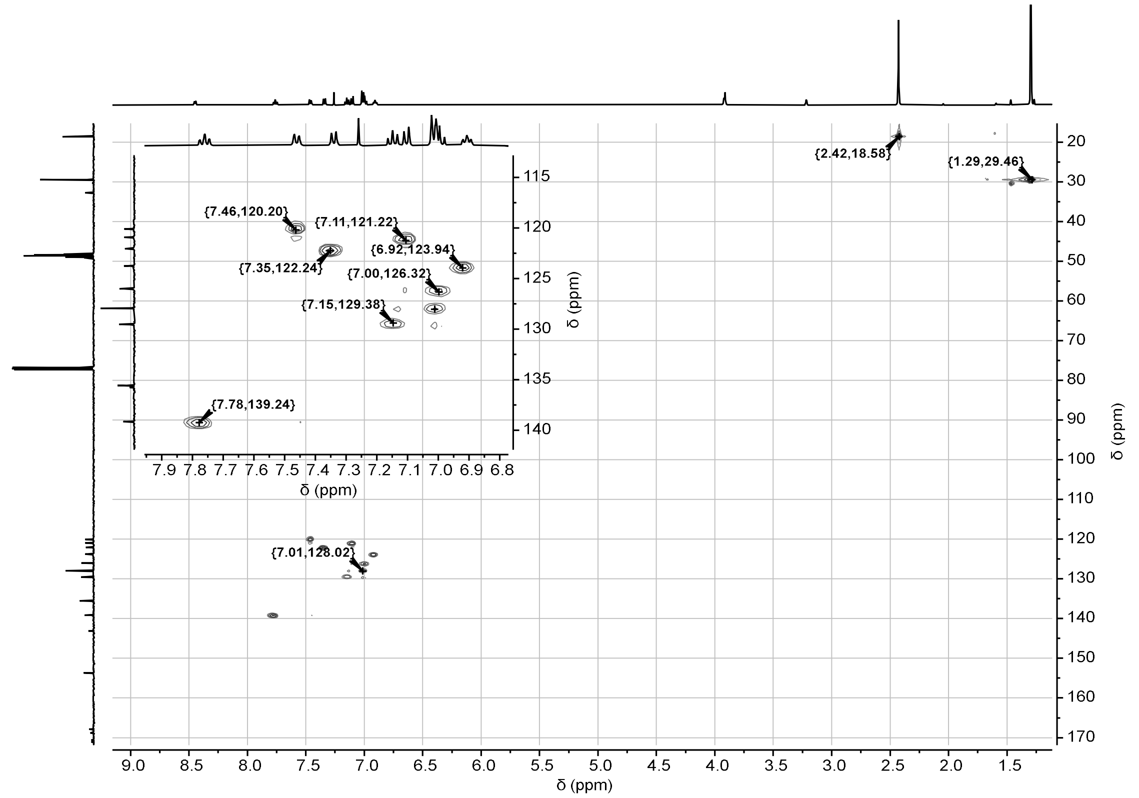

Figure S34. ^{1}H,^{13}C-HSQC-NMR spectrum (500 MHz, 126 MHz, $CDCl_3$/methanol-d_4) of **[LPtCNPhen]**.

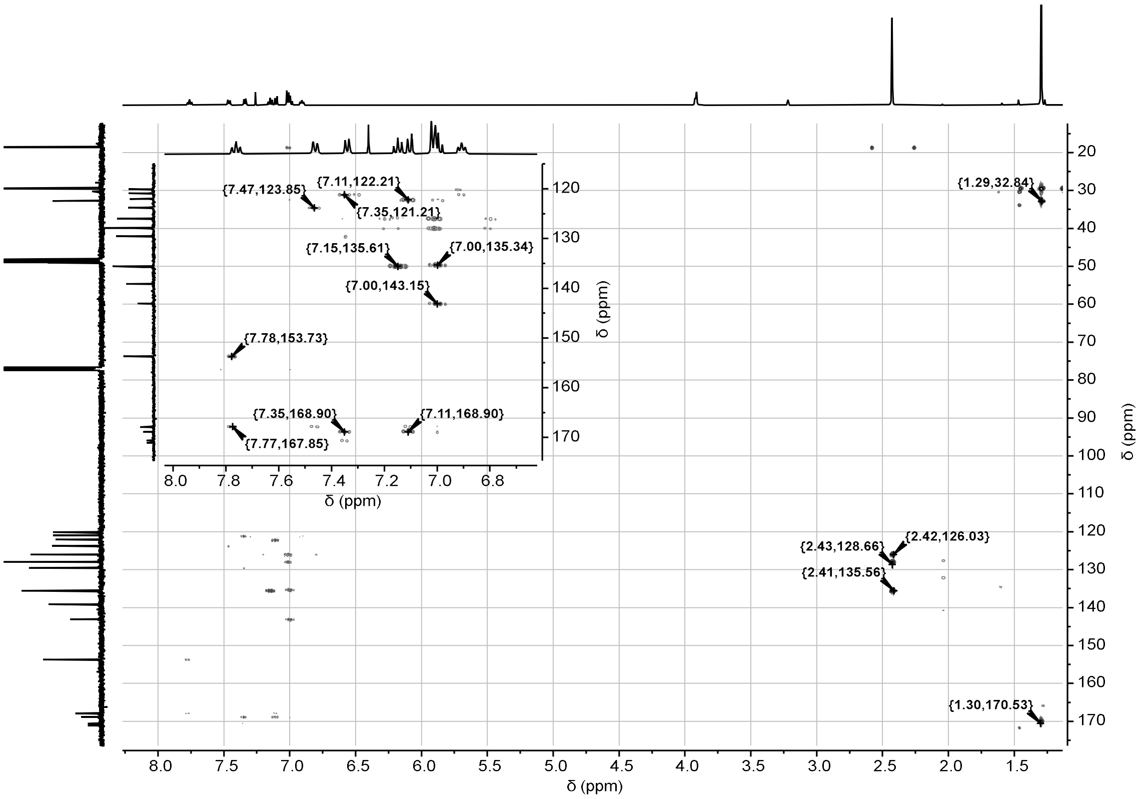

Figure S35. $^{1}H,^{13}C$-HMBC-NMR spectrum (500 MHz, 126 MHz, $CDCl_3$/methanol-d_4) of **[LPtCNPhen]**.

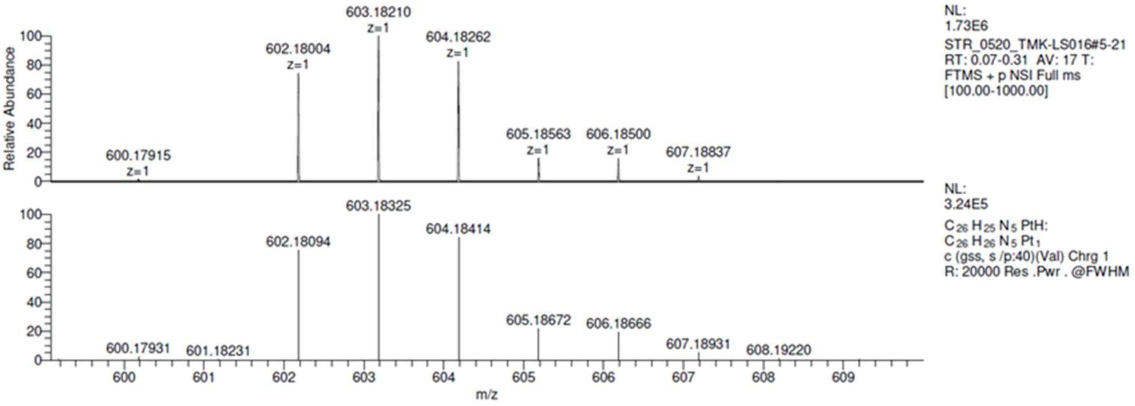

Figure S36. Mass spectrum of **[LPtCNPhen]** (top). Additional simulation of the [**[LPtCNPhen]**-H]$^{+}$ adduct (bottom).

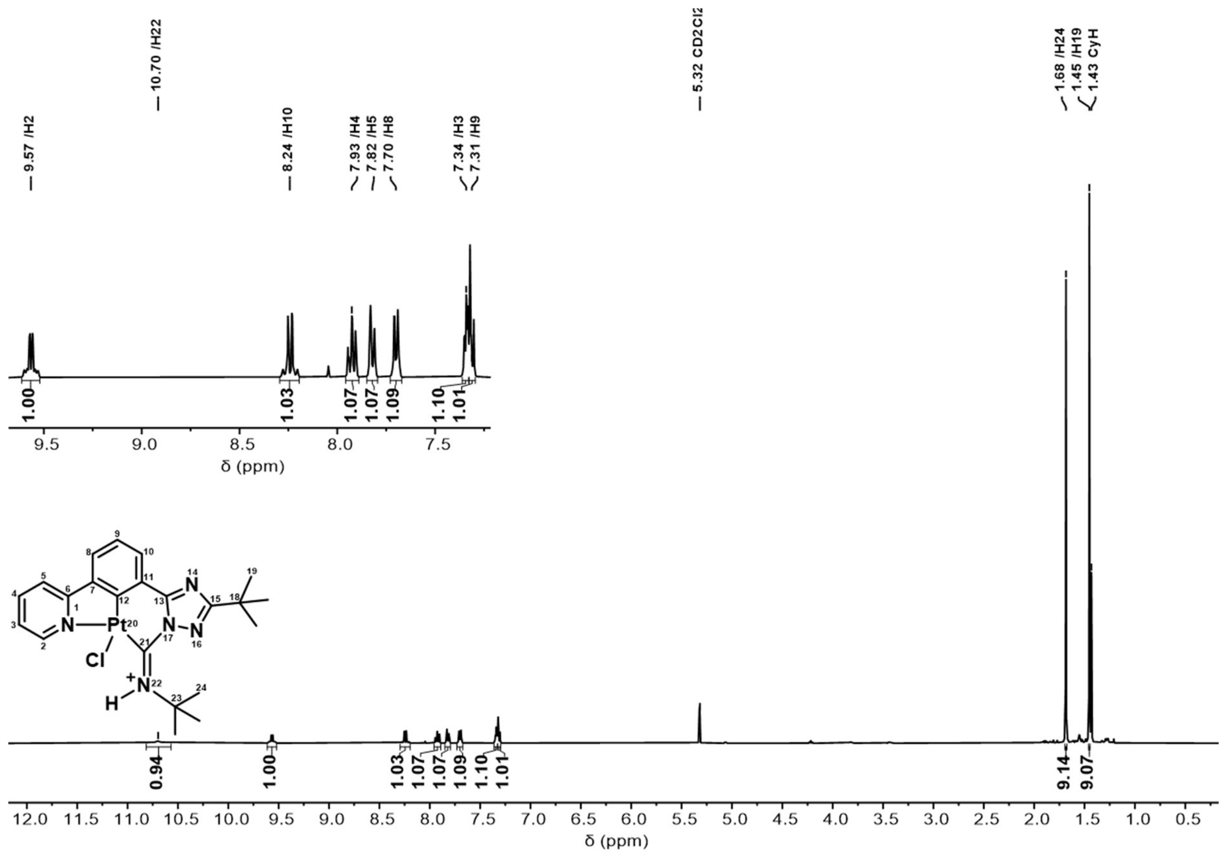

Figure S37. ^{1}H-NMR spectrum (400 MHz, CD_2Cl_2) of **[LPtCN*t*Bu]**.

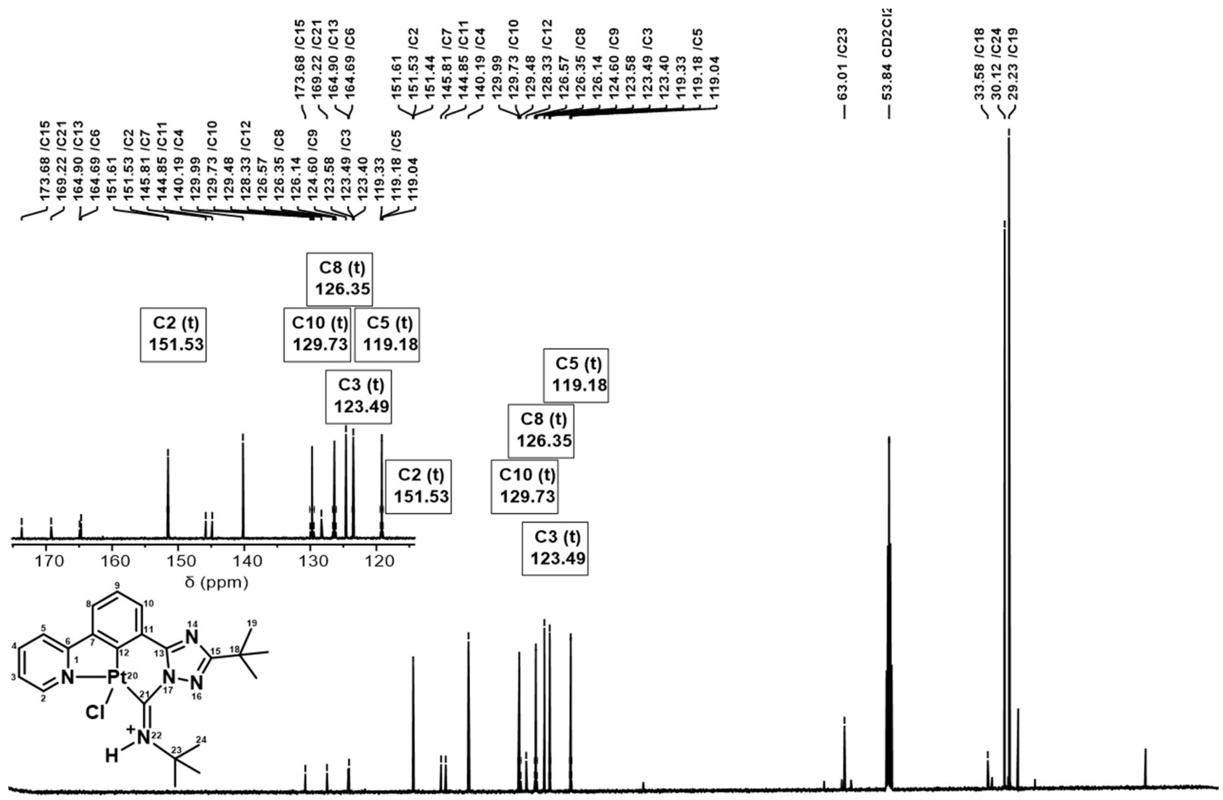

Figure S38. ^{13}C-NMR spectrum (101 MHz, CD_2Cl_2) of **[LPtCN*t*Bu]**.

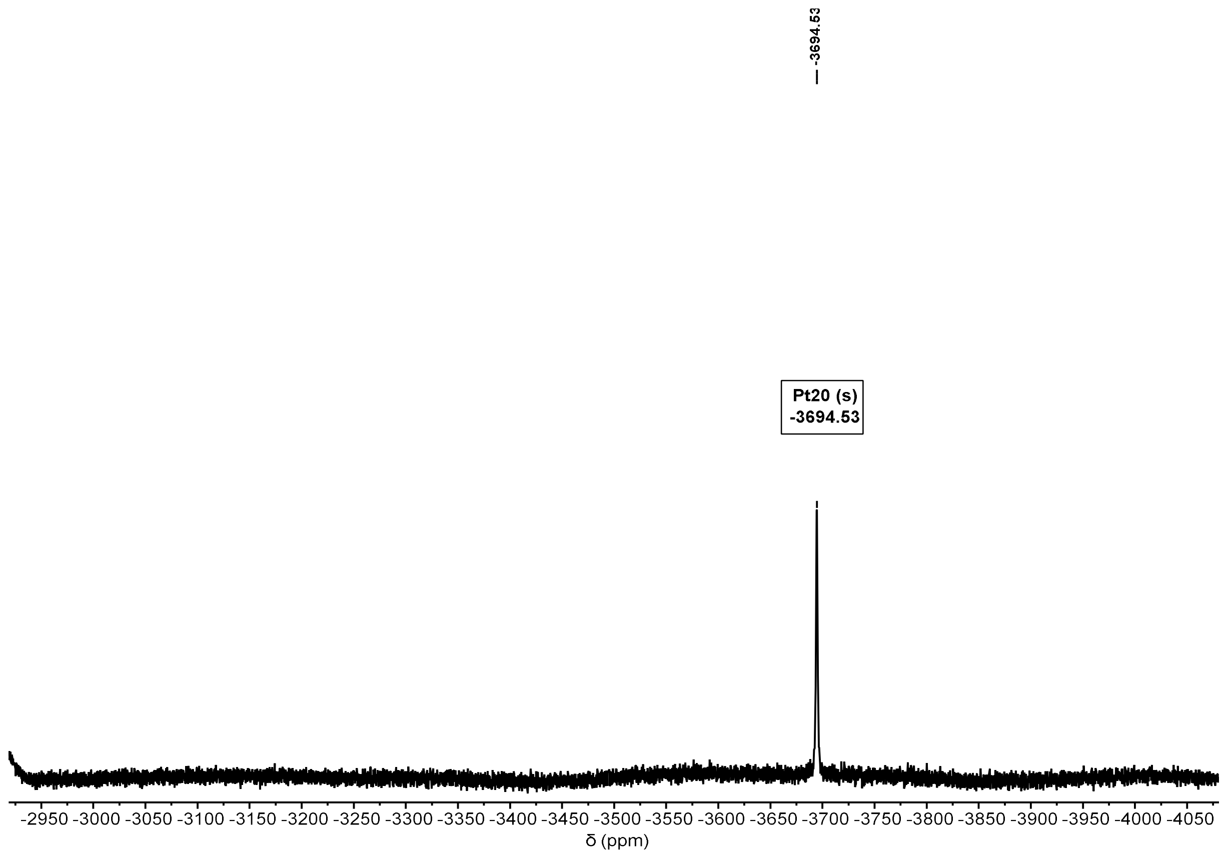

Figure S39. ^{195}Pt-NMR spectrum (86 MHz, CD_2Cl_2) of **[LPtCN*t*Bu]**.

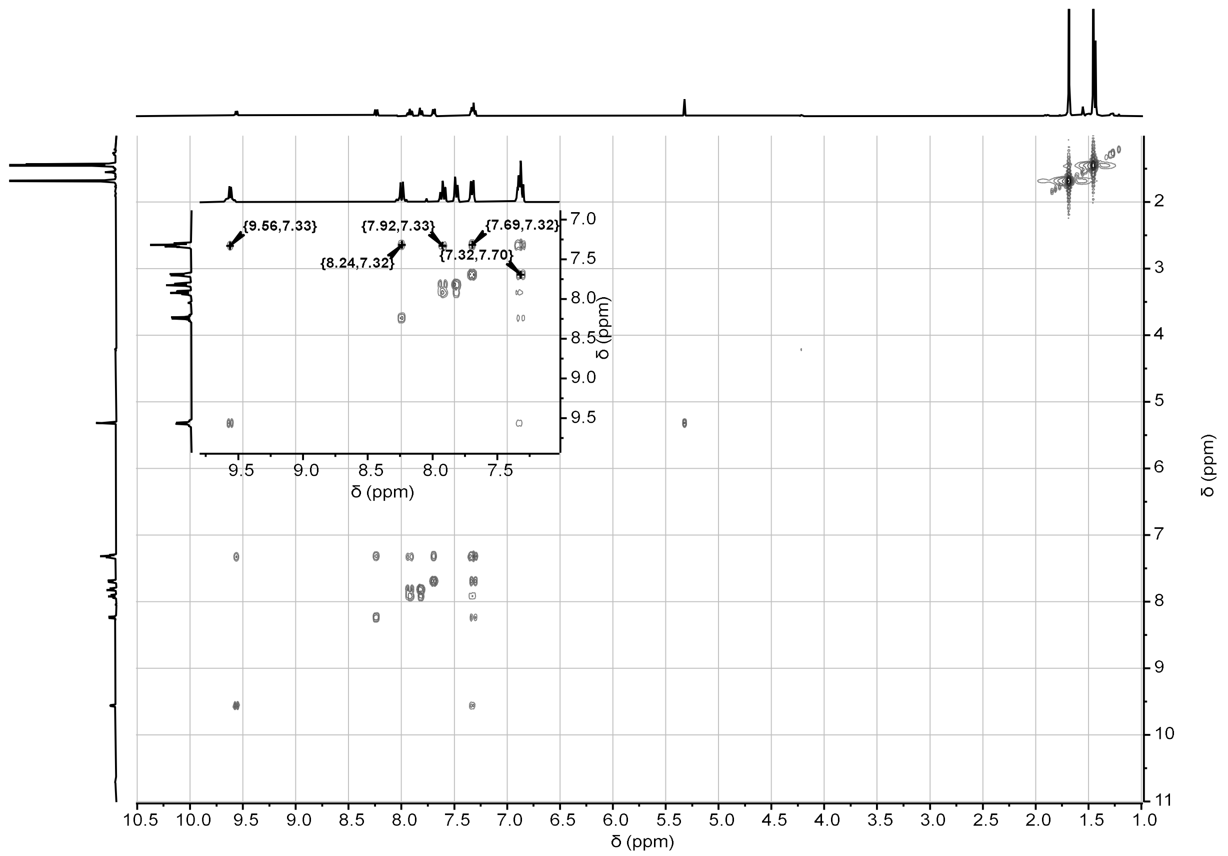

Figure S40. ^{1}H,^{1}H-COSY-NMR spectrum (400 MHz, CD_2Cl_2) of **[LPtCN*t*Bu]**.

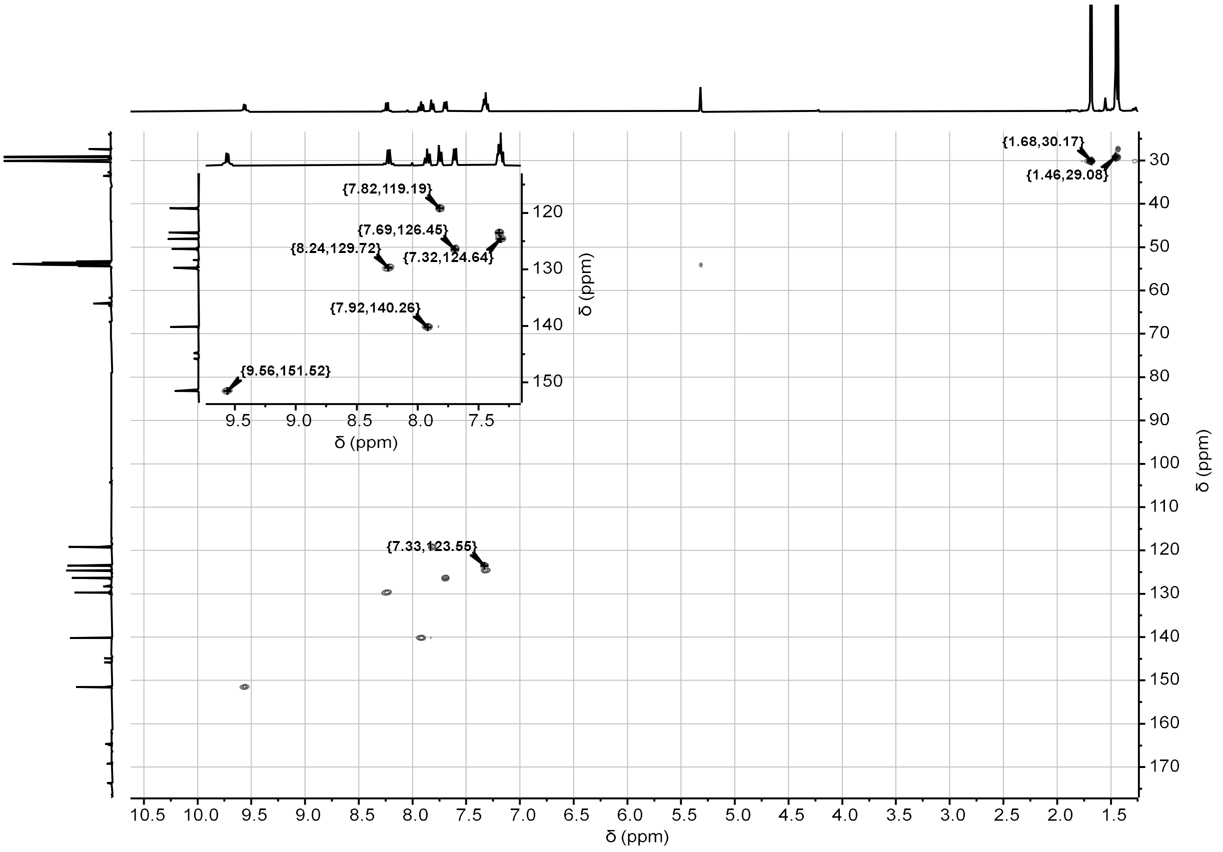

Figure S41. $^{1}H,^{13}C$-HSQC-NMR spectrum (400 MHz, 101 MHz, CD_2Cl_2) of **[LPtCN*t*Bu]**.

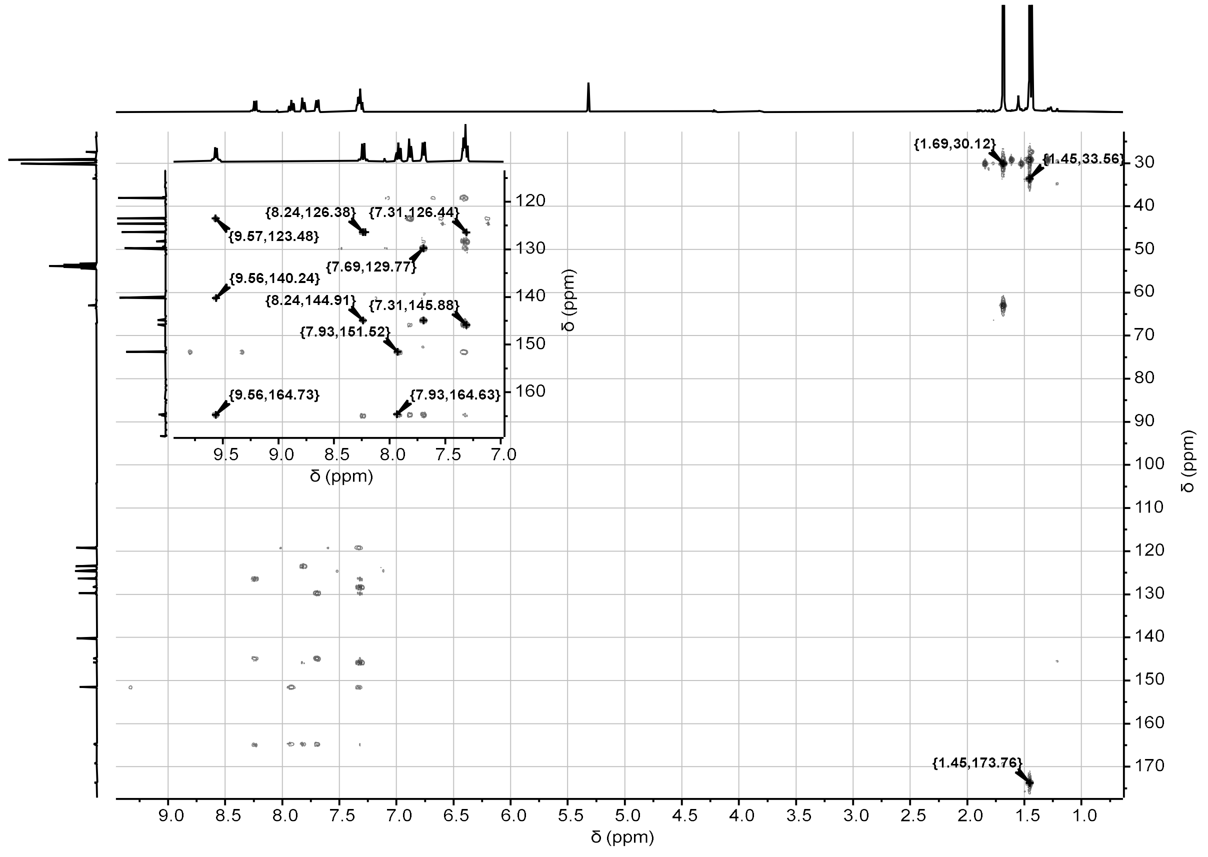

Figure S42. $^{1}H,^{13}C$-HMBC-NMR spectrum (400 MHz, 101 MHz, CD_2Cl_2) of **[LPtCN*t*Bu]**.

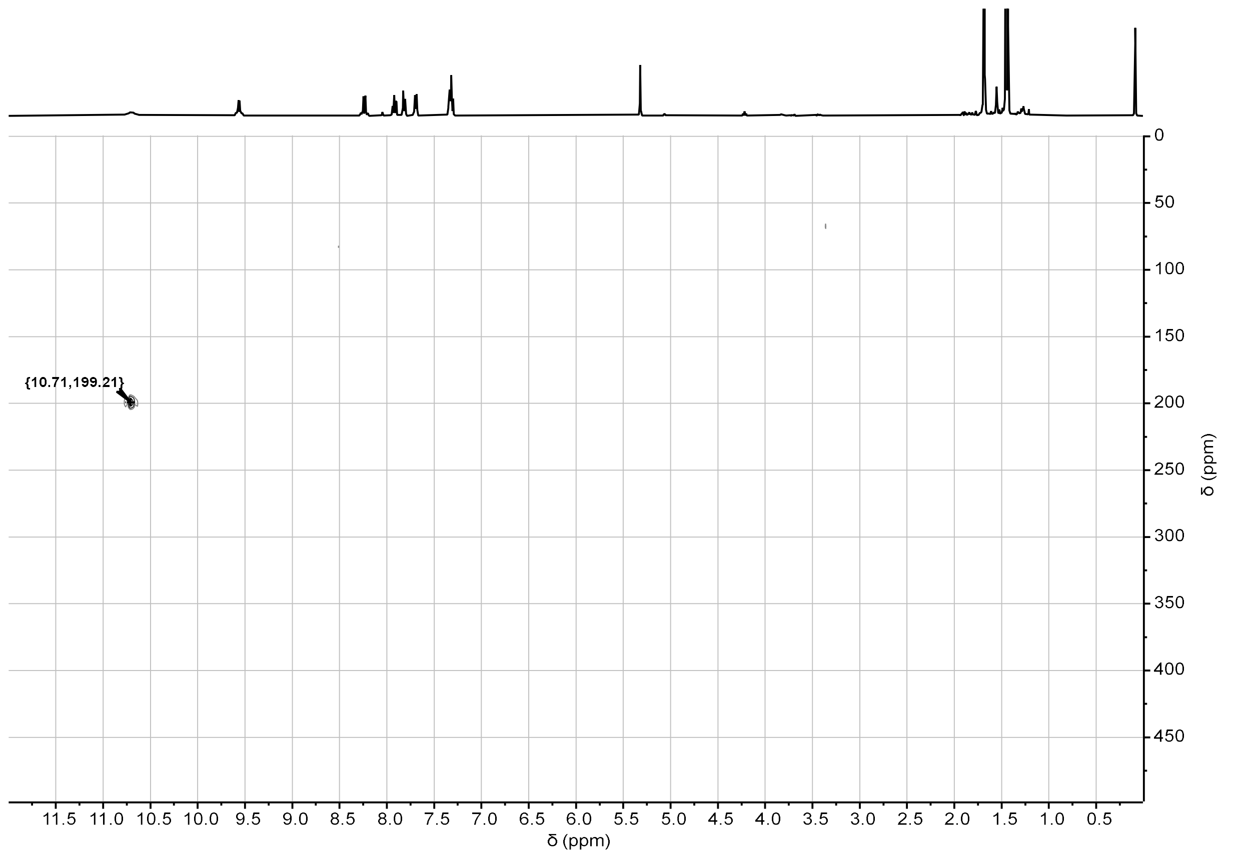

Figure S43. $^{1}H,^{15}N$-HSQC-NMR spectrum (400 MHz, 41 MHz, CD_2Cl_2) of **[LPtCN*t*Bu]**.

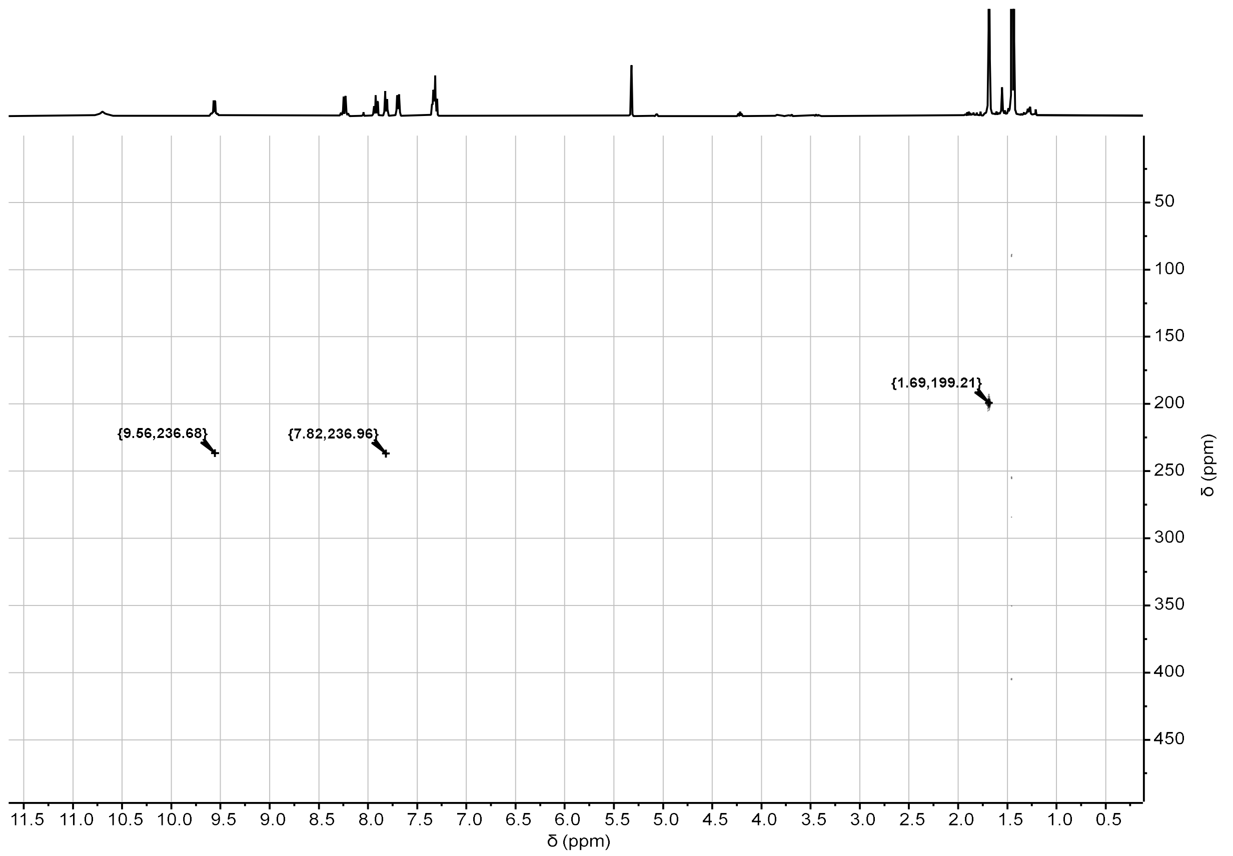

Figure S44. $^{1}H,^{15}N$-HMBC-NMR spectrum (400 MHz, 41 MHz, CD_2Cl_2) of **[LPtCN*t*Bu]**.

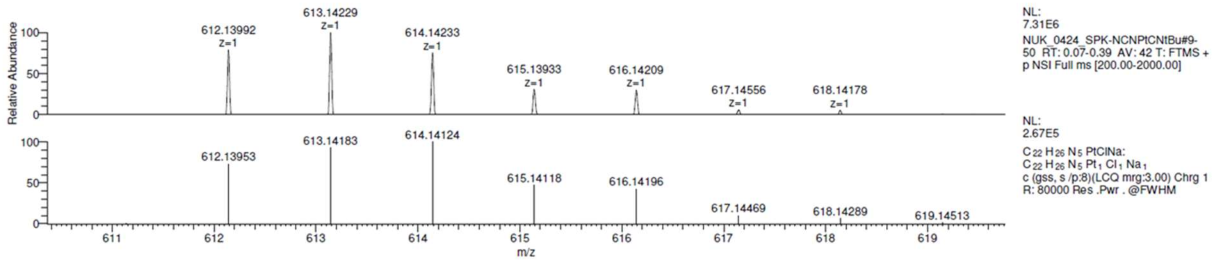

Figure S45. Mass spectrum of **[LPtCN*t*Bu]** (top). Additional simulation of the [**[LPtCN*t*Bu]**+Na]$^+$ adduct (bottom).

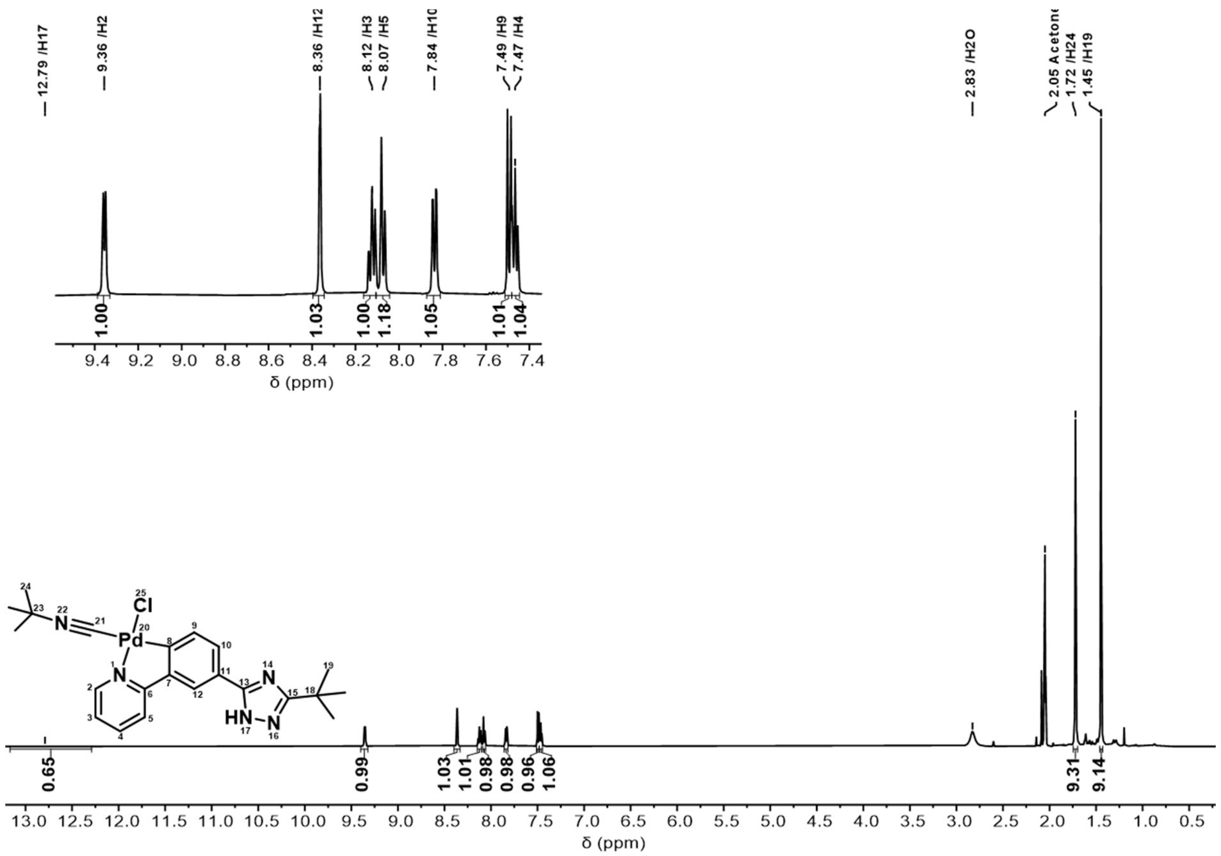

Figure S46. ^{1}H-NMR spectrum (500 MHz, acetone-d_6) of **[HLPdCN*t*Bu]**.

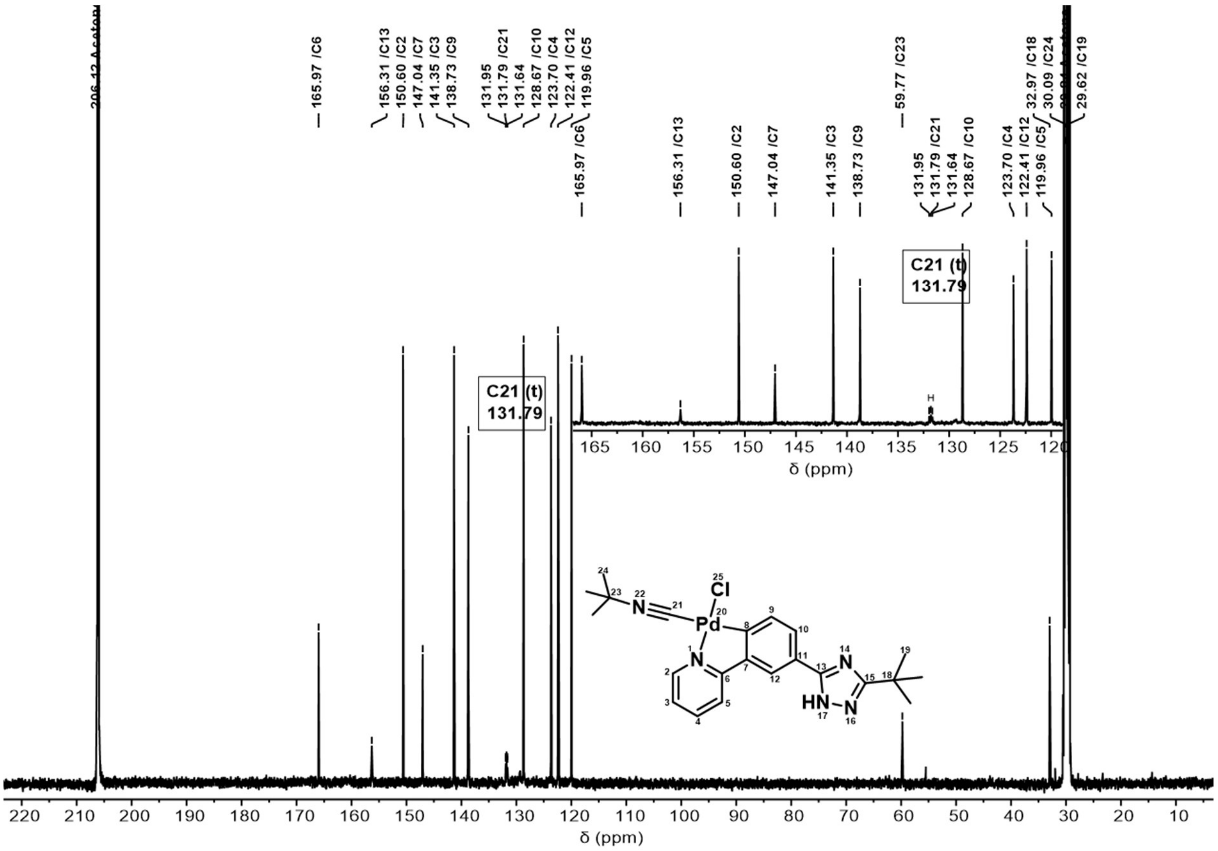

Figure S47. ^{13}C-NMR spectrum (126 MHz, acetone-d_6) of **[HLPdCN*t*Bu]**.

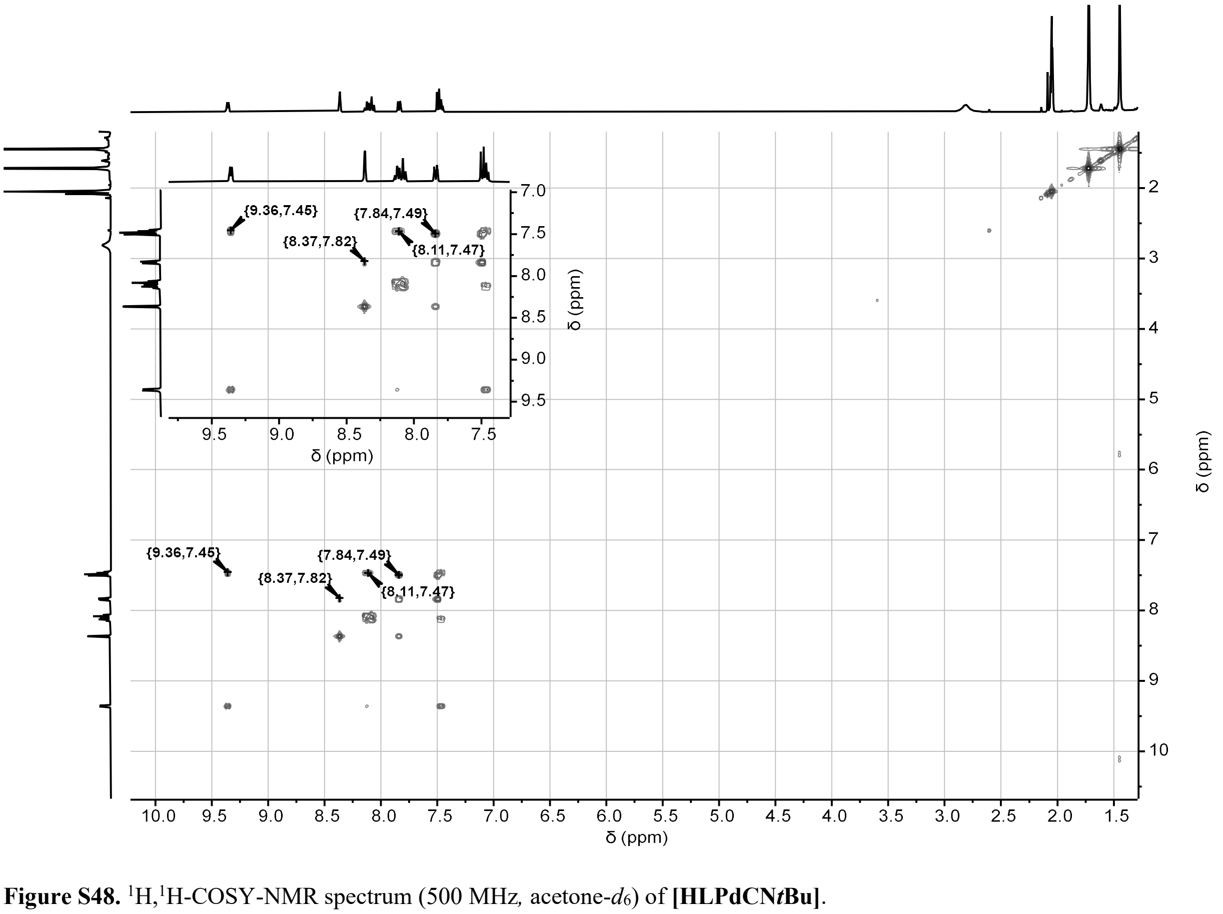

Figure S48. ^{1}H,^{1}H-COSY-NMR spectrum (500 MHz, acetone-d_6) of **[HLPdCN*t*Bu]**.

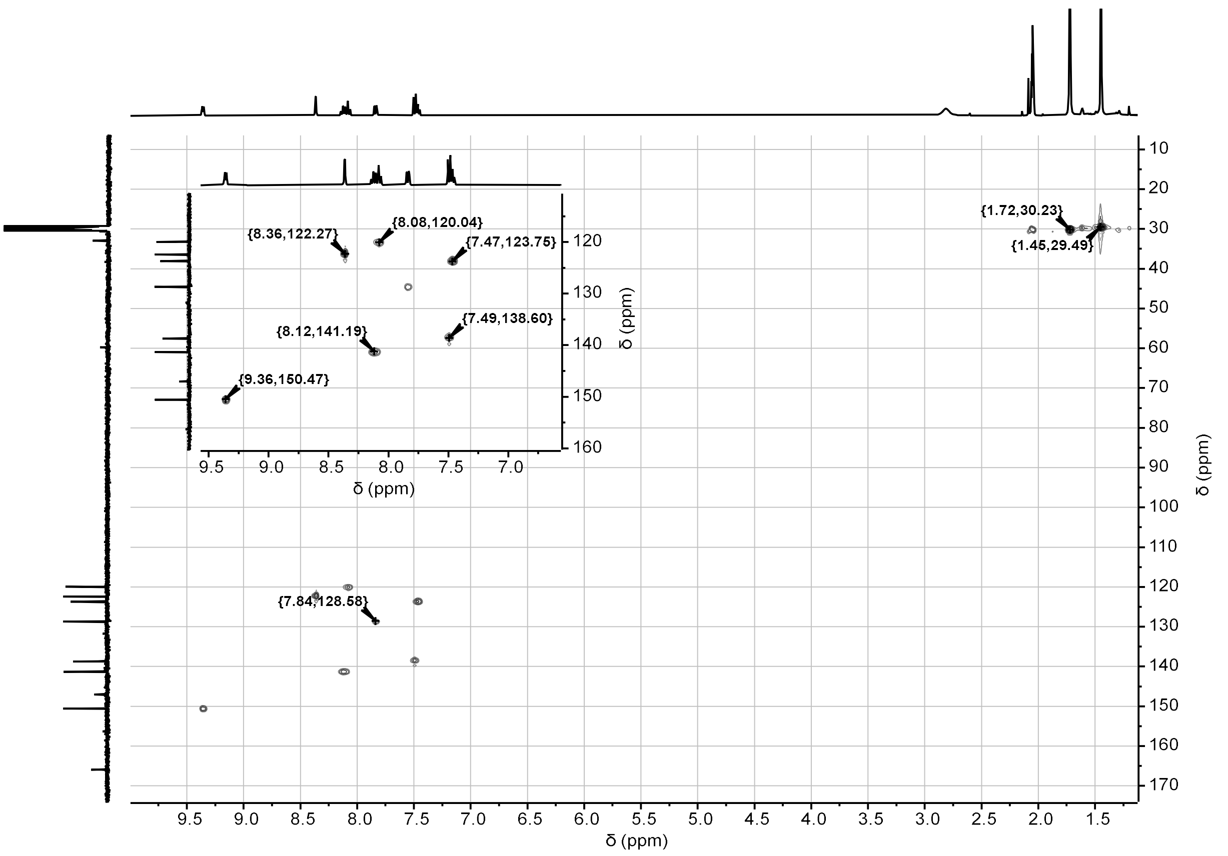

Figure S49. ^{1}H,^{13}C-HSQC-NMR spectrum (500 MHz, 126 MHz, acetone-*d*$_6$) of **[HLPdCN*t*Bu]**.

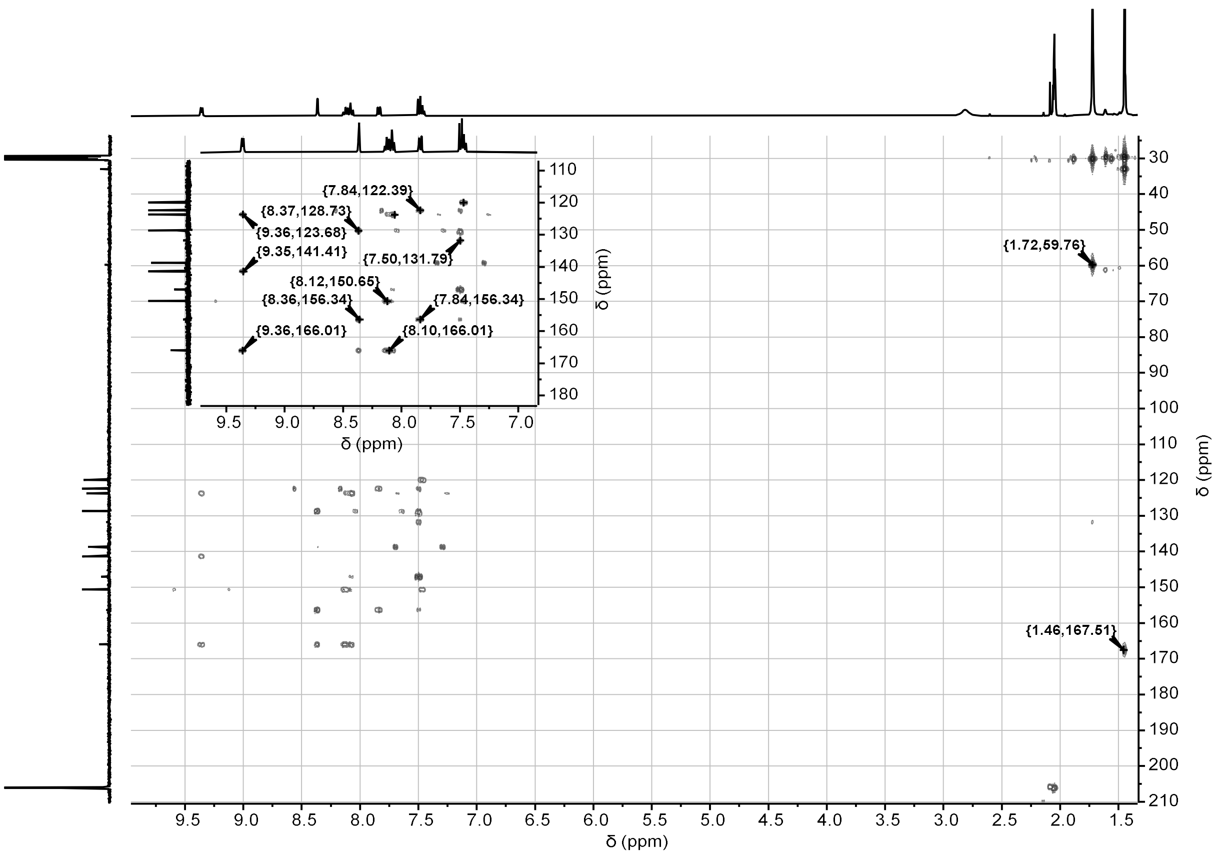

Figure S50. ^{1}H,^{13}C-HMBC-NMR spectrum (500 MHz, 126 MHz, acetone-*d*$_6$) of **[HLPdCN*t*Bu]**.

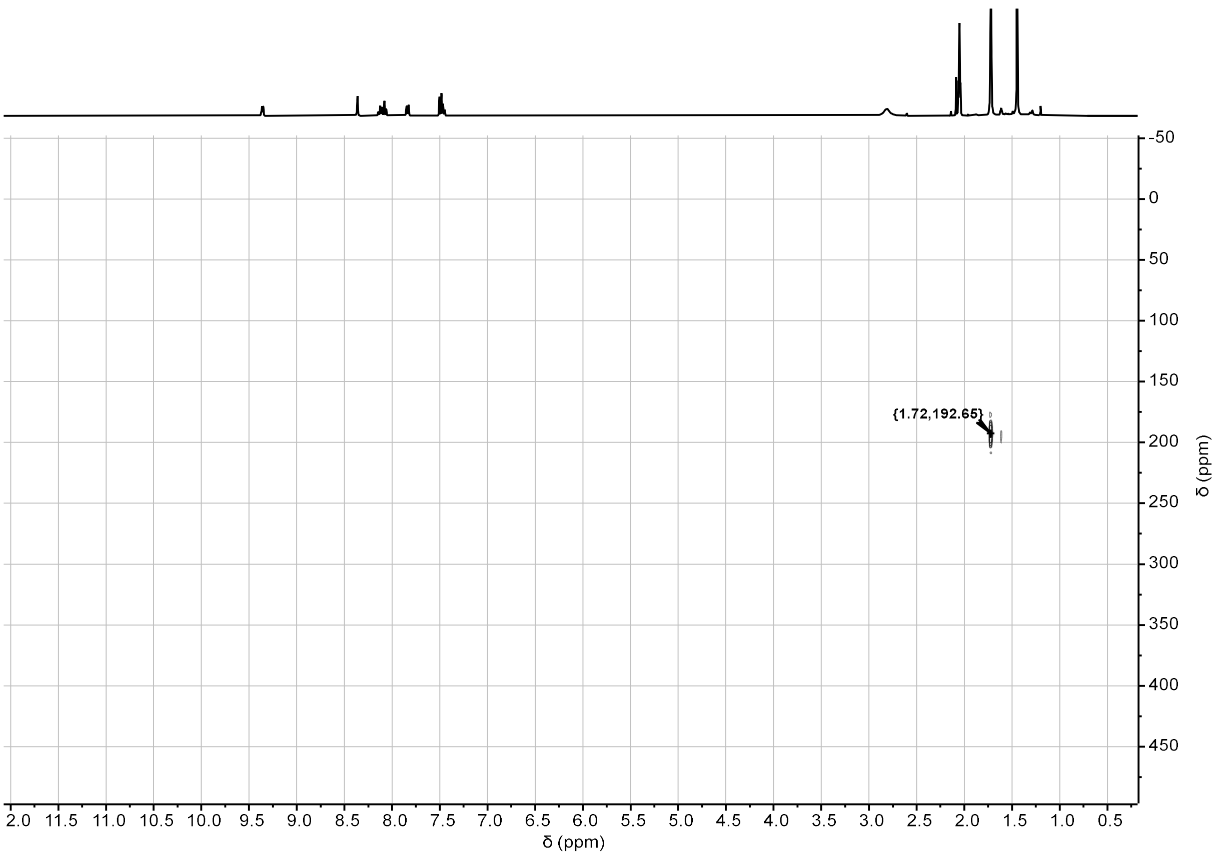

Figure S51. $^{1}H,^{15}N$-HMBC-NMR spectrum (500 MHz, 51 MHz, acetone-d_6) of **[HLPdCN*t*Bu]**.

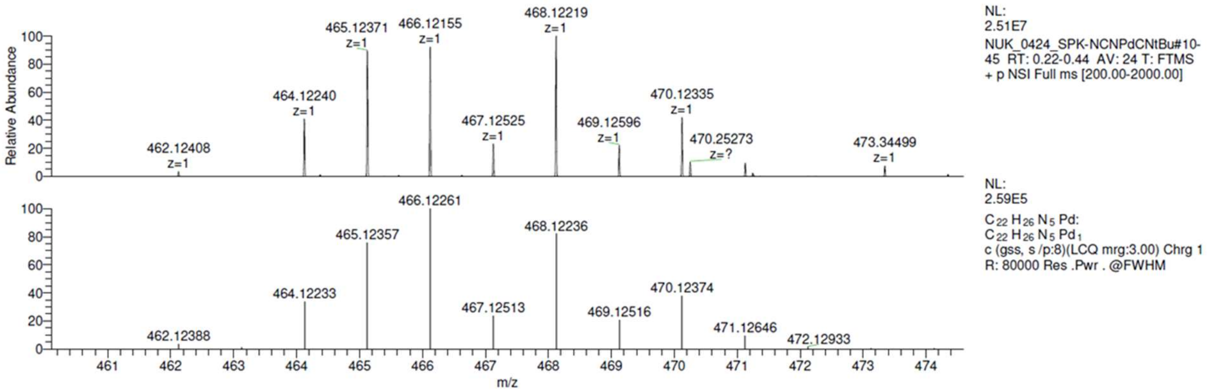

Figure S52. Mass spectrum of **[HLPdCN*t*Bu]** (top). Additional simulation of the [**[HLPdCN*t*Bu]**-Cl]$^{+}$ adduct (bottom).

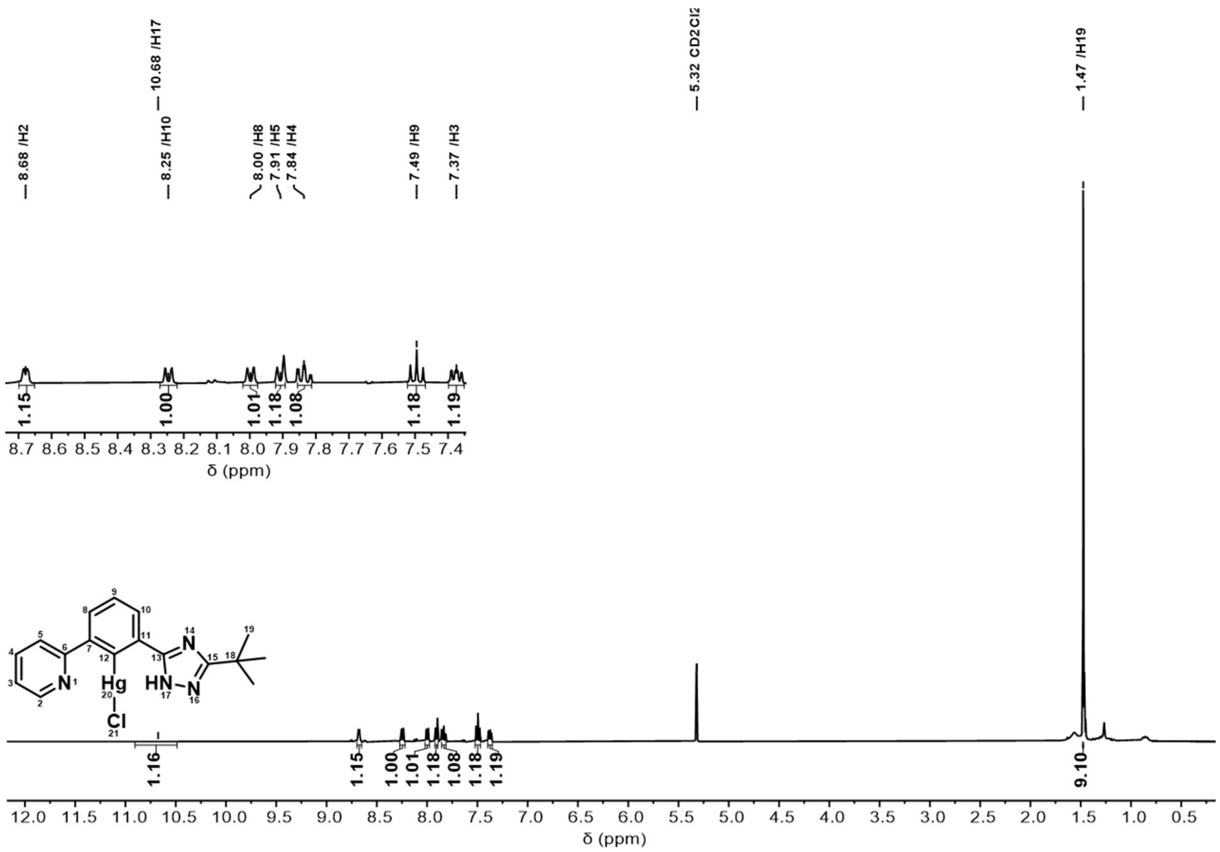

Figure S53. ^{1}H-NMR spectrum (400 MHz, CD_2Cl_2) of **[HLHgCl]**.

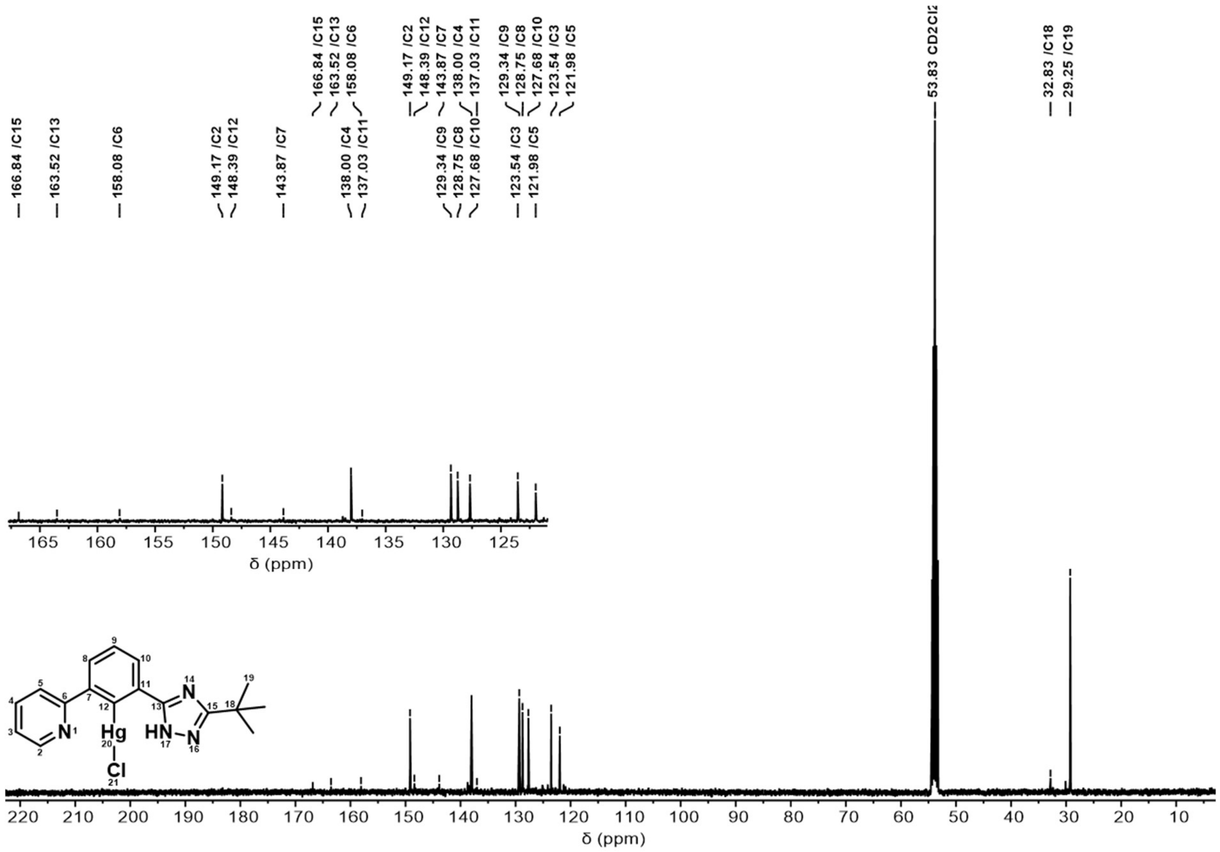

Figure S54. ^{13}C-NMR spectrum (101 MHz, CD_2Cl_2) of **[HLHgCl]**.

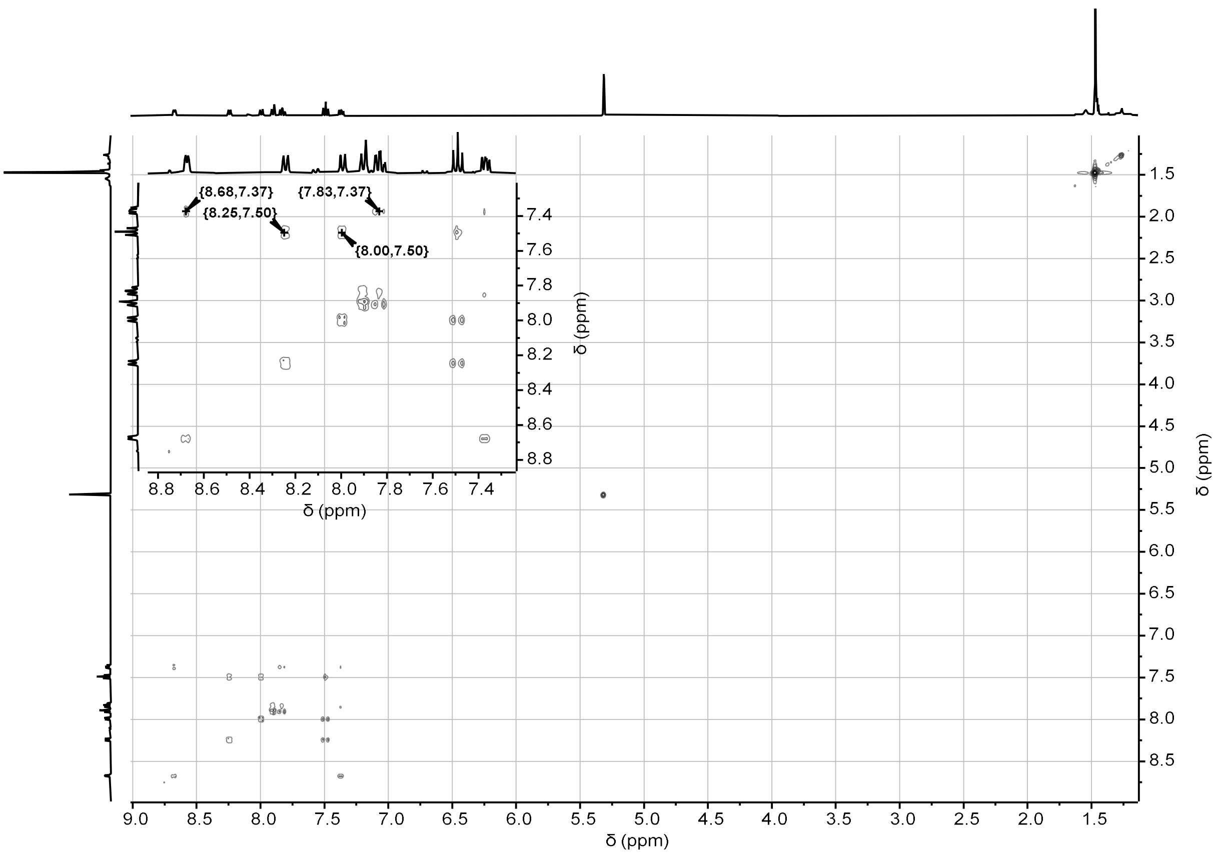

Figure S55. $^{1}H,^{1}H$-COSY-NMR spectrum (400 MHz, CD_2Cl_2) of **[HLHgCl]**.

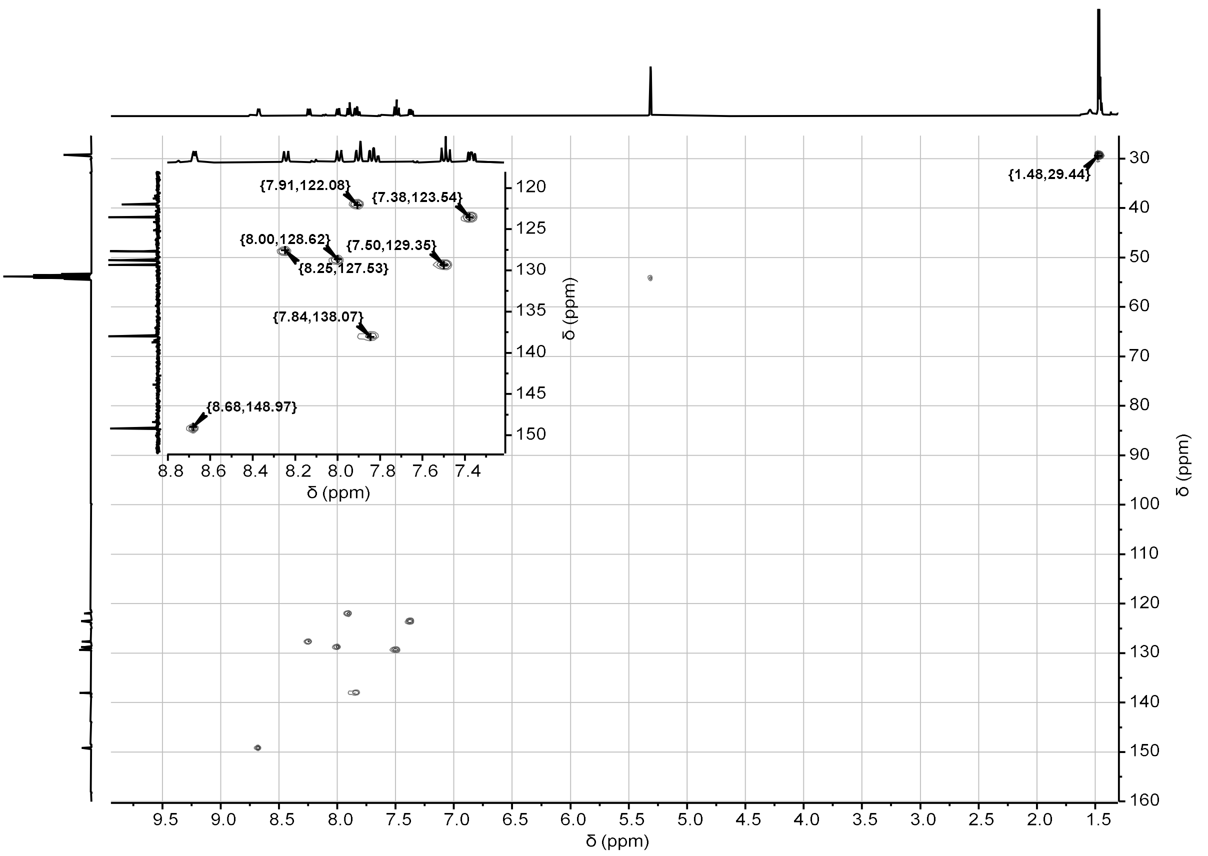

Figure S56. $^{1}H,^{13}C$-HSQC-NMR spectrum (400 MHz, 101 MHz, CD_2Cl_2) of **[HLHgCl]**.

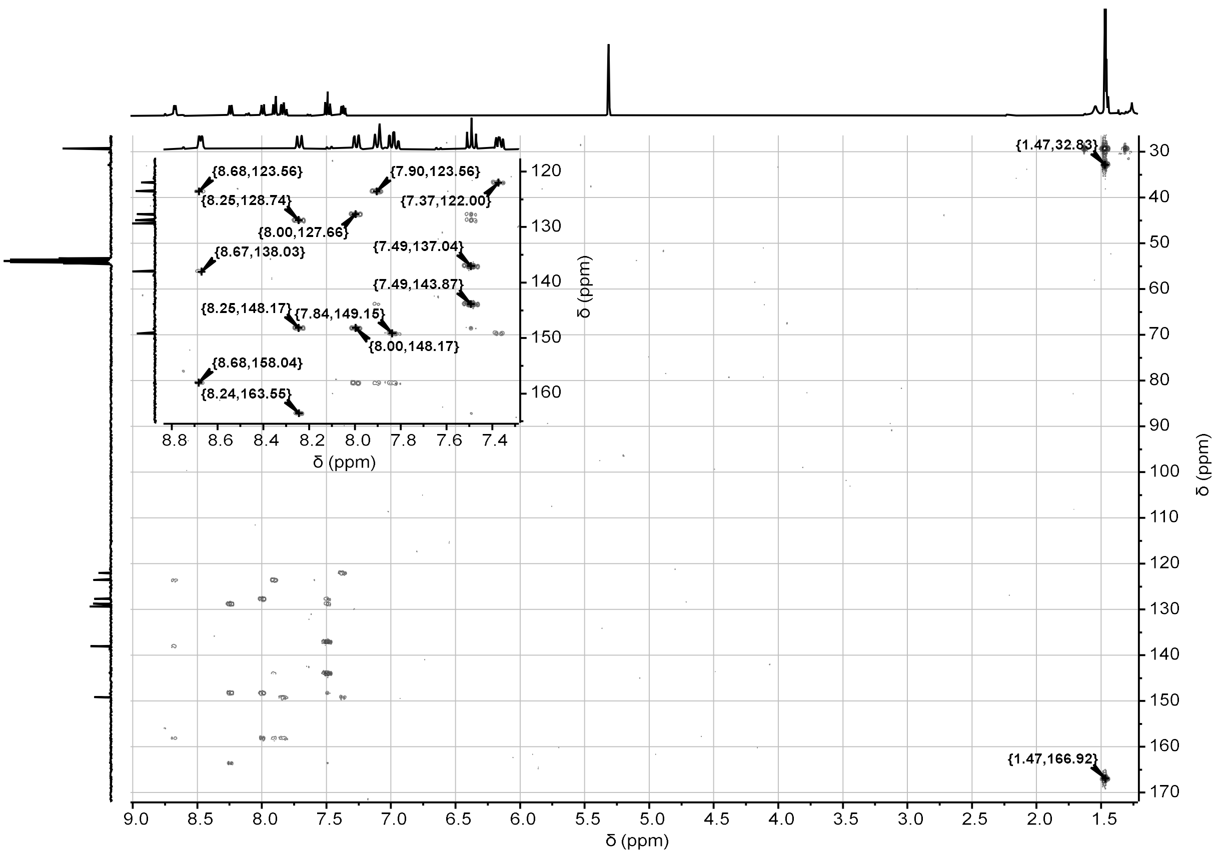

Figure S57. $^{1}H,^{13}C$-HMBC-NMR spectrum (400 MHz, 101 MHz, CD_2Cl_2) of **[HLHgCl]**.

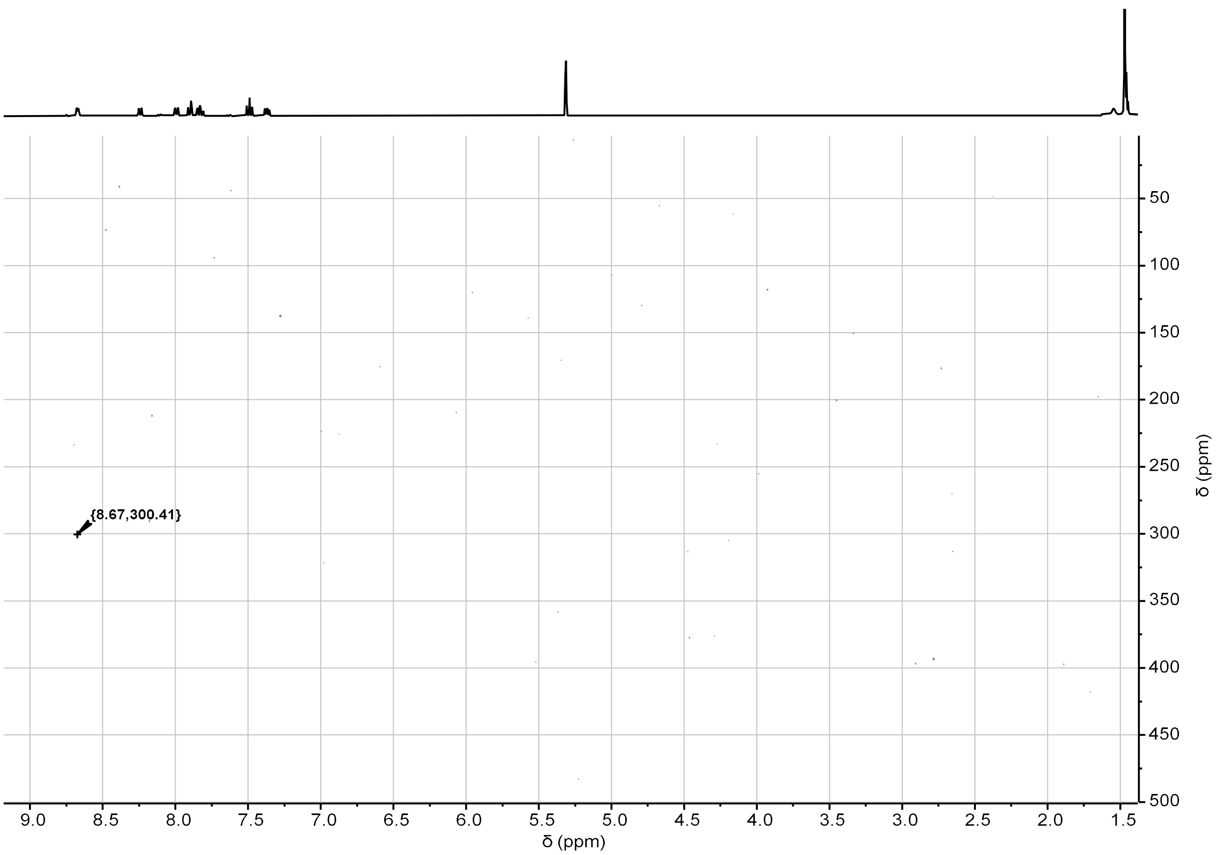

Figure S58. $^{1}H,^{15}N$-HMBC-NMR spectrum (400 MHz, 41 MHz, CD_2Cl_2) of **[HLHgCl]**.

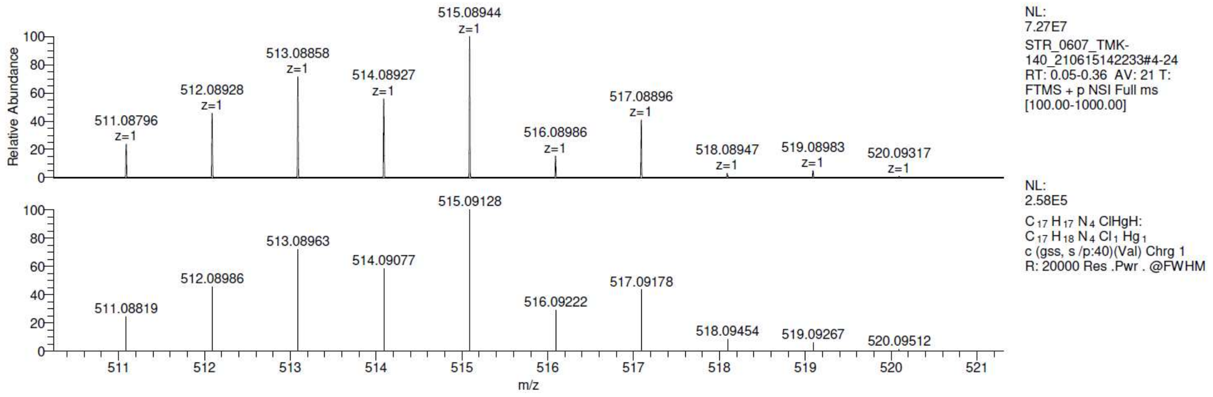

Figure S59. Mass spectrum of **[HLHgCl]** (top). Additional simulation of the [**[HLHgCl]**+H]$^+$ adduct (bottom).

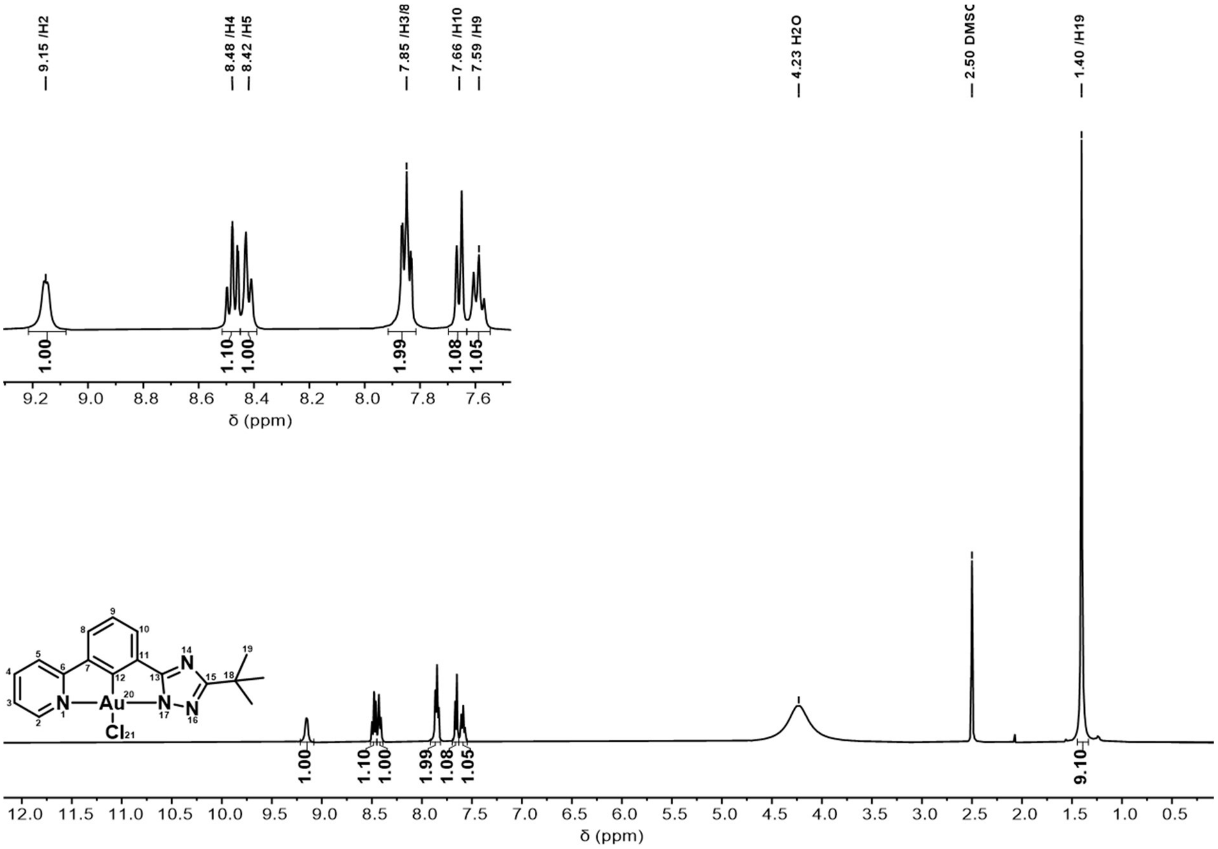

Figure S60. ^{1}H-NMR spectrum (400 MHz, DMSO-d_6) of **[LAuCl]**.

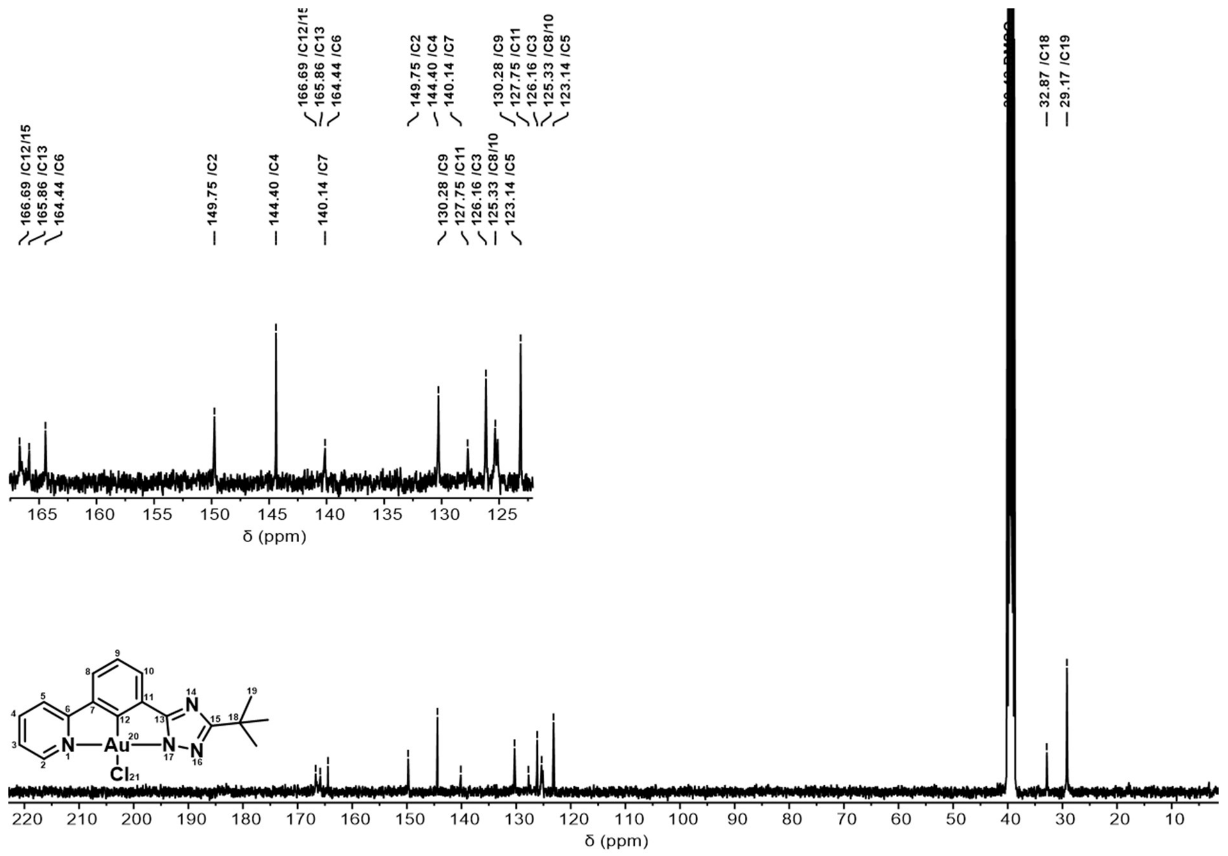

Figure S61. ^{13}C-NMR spectrum (101 MHz, DMSO-d_6) of **[LAuCl]**.

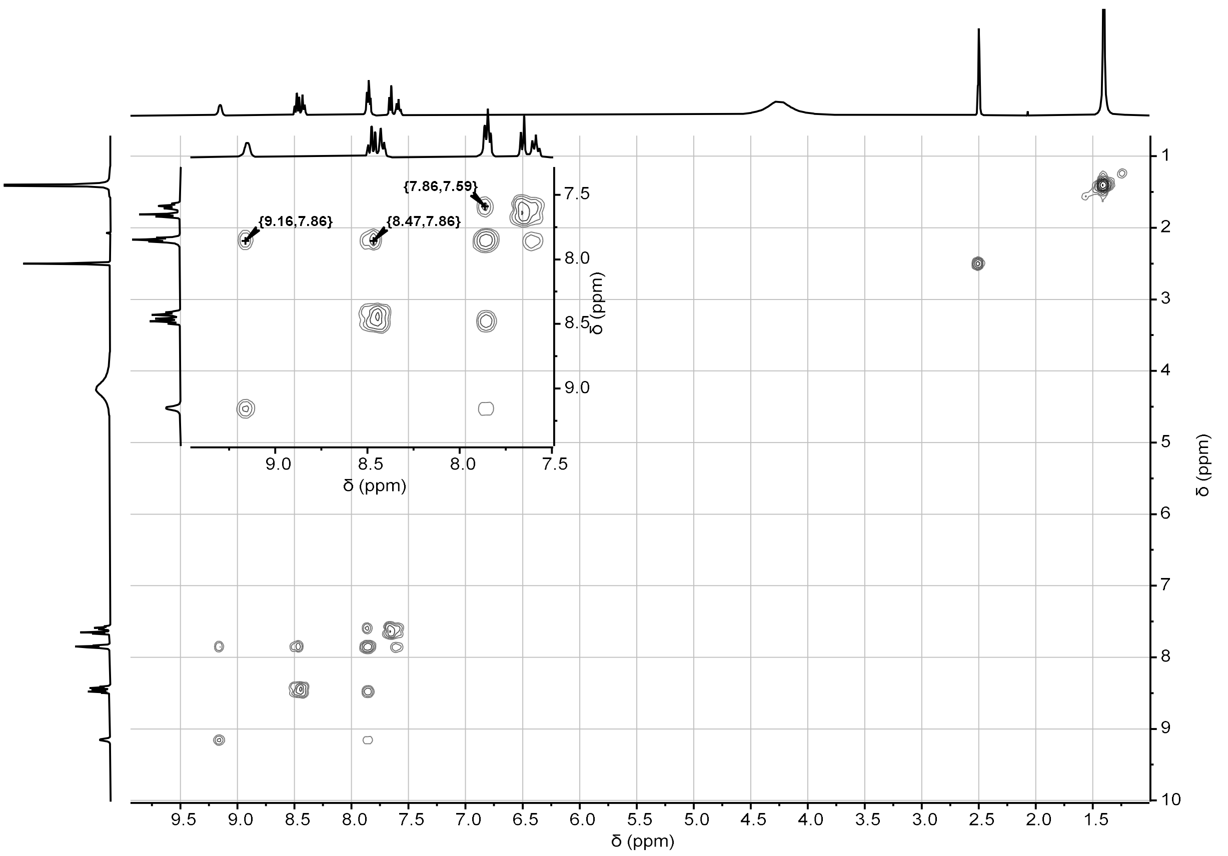

Figure S62. ^{1}H,^{1}H-COSY-NMR spectrum (400 MHz, DMSO-d_6) of **[LAuCl]**.

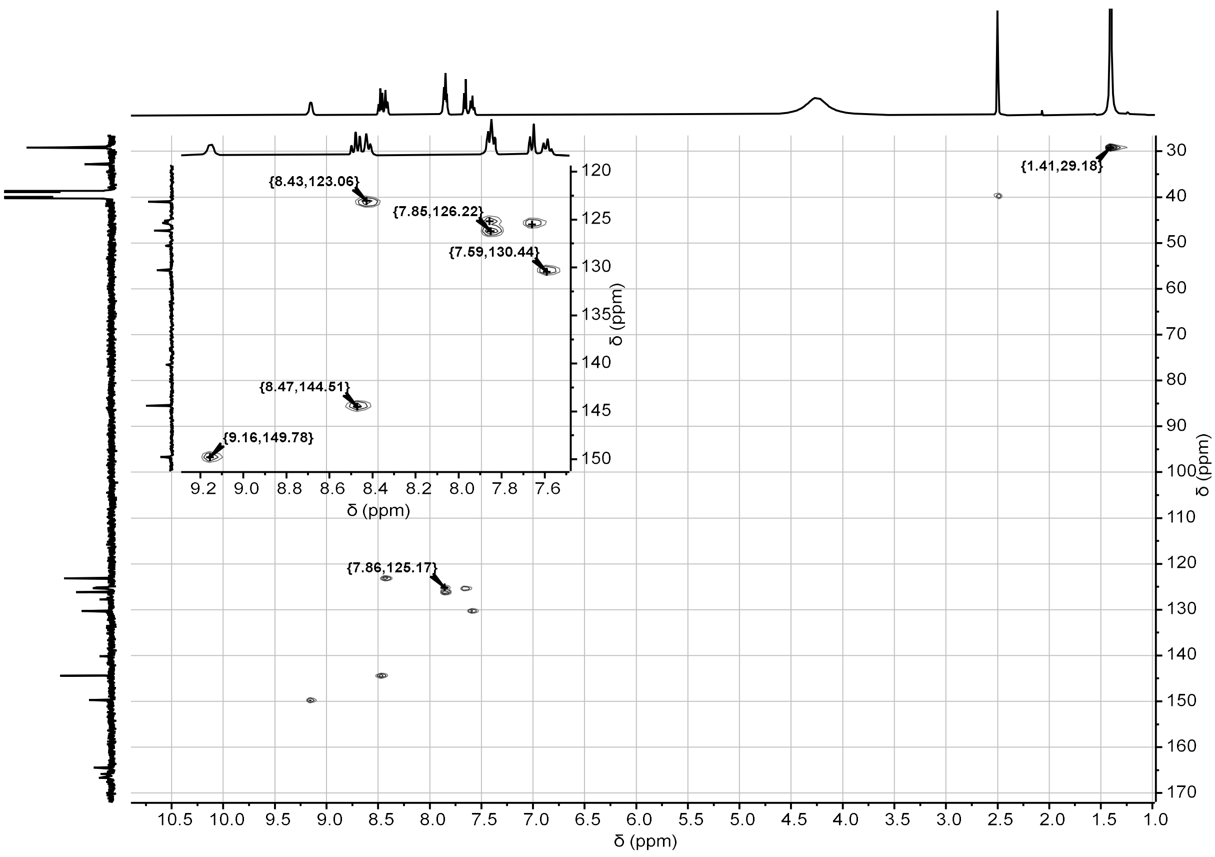

Figure S63. $^{1}H,^{13}C$-HSQC-NMR spectrum (400 MHz, 101 MHz, DMSO-d_6) of **[LAuCl]**.

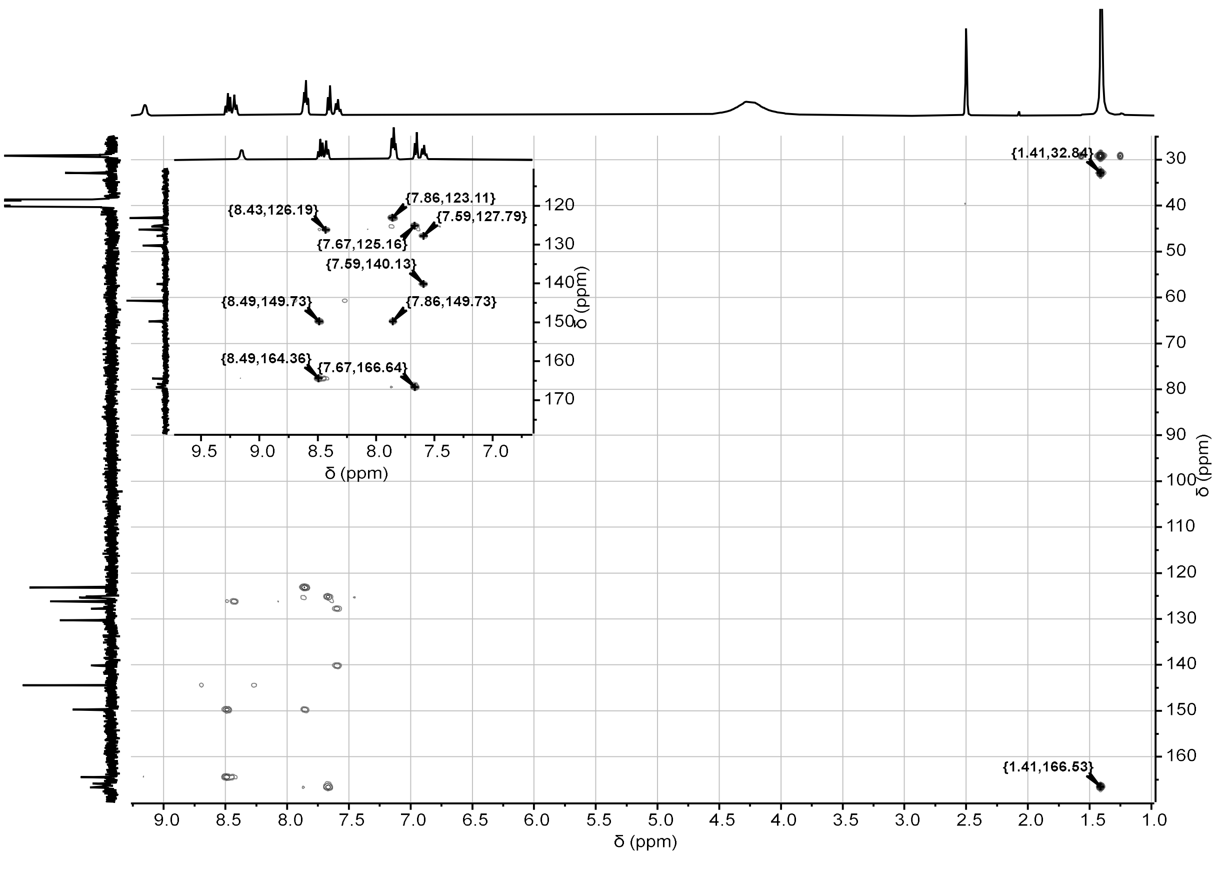

Figure S64. $^{1}H,^{13}C$-HMBC-NMR spectrum (400 MHz, 101 MHz, DMSO-d_6) of **[LAuCl]**.

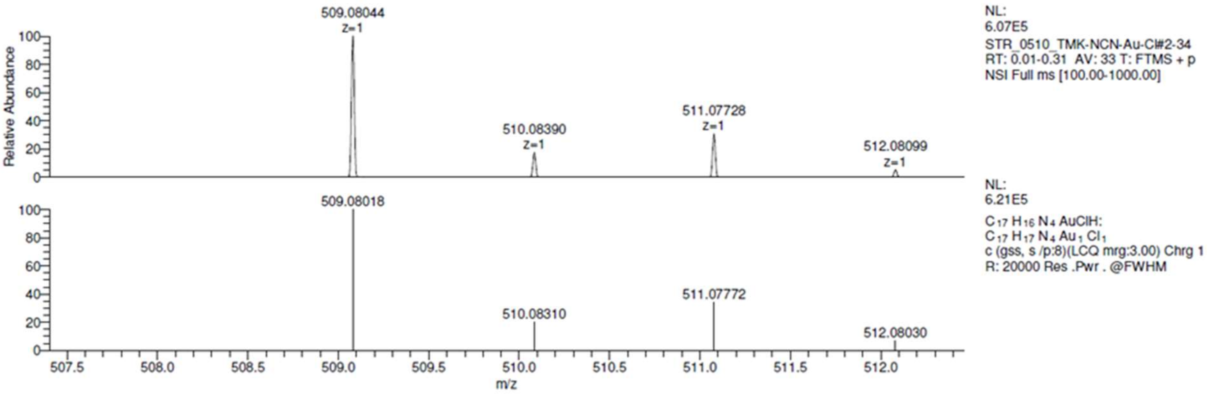

Figure S65. Mass spectrum of **[LAuCl]** (top). Additional simulation of the [**[LAuCl]**+H]$^+$ adduct (bottom).

X-ray diffractometry on single crystals

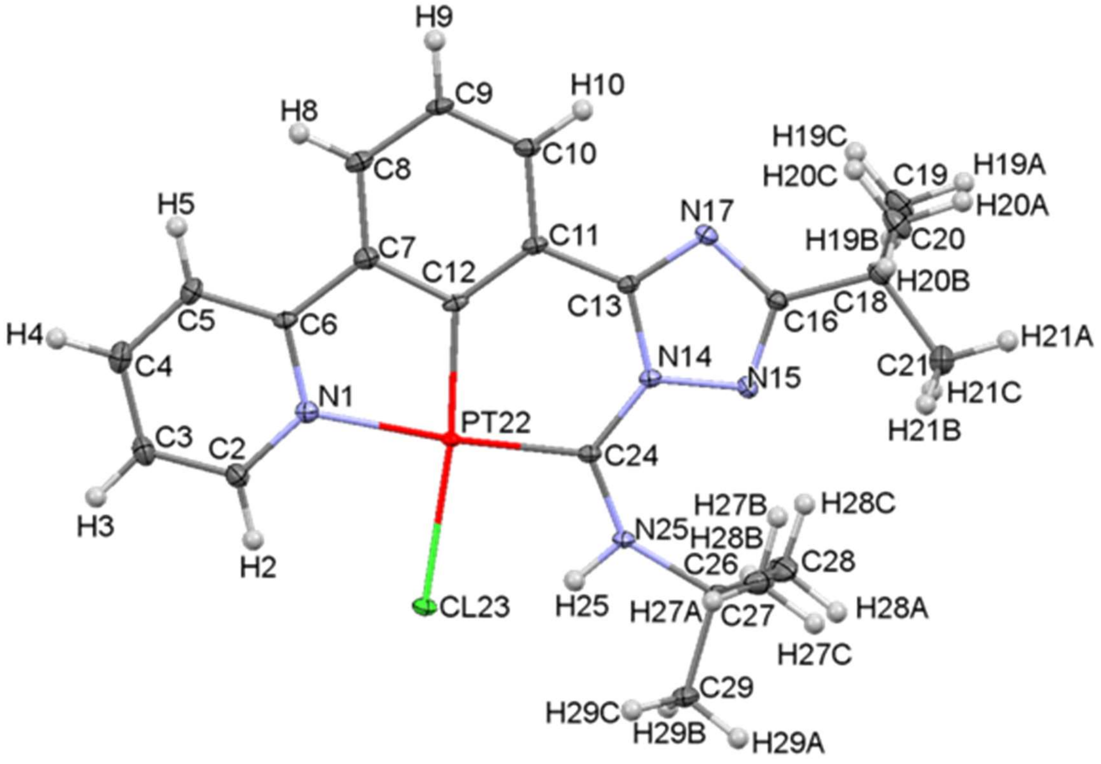

Figure S66. Molecular structure as ORTEP diagram (displacement ellipsoids are shown at 50% probability) of **[LPtCNtBu]**.

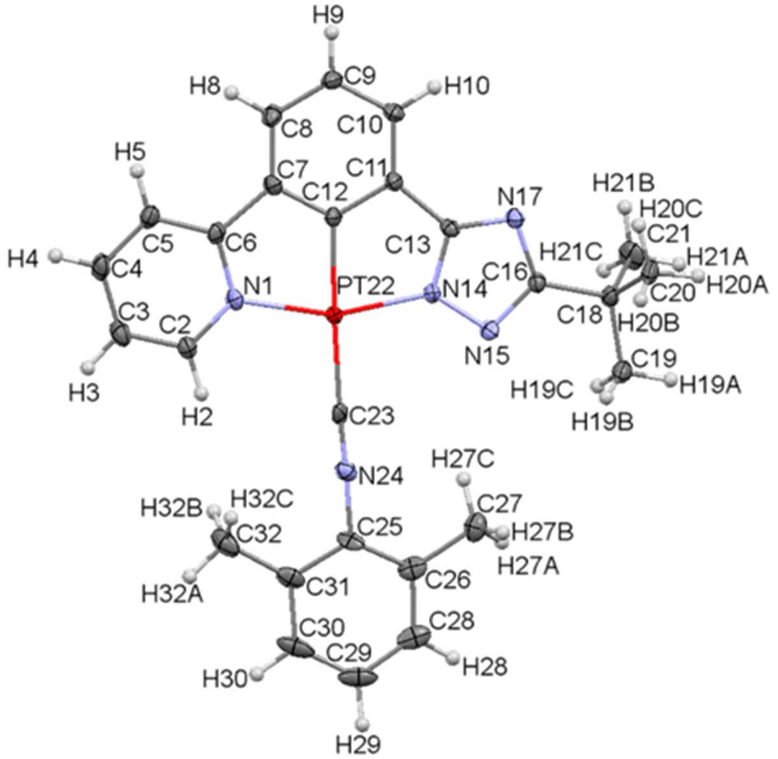

Figure S67. Molecular structure as ORTEP diagram (displacement ellipsoids are shown at 50% probability) of **[LPtCNPhen]**.

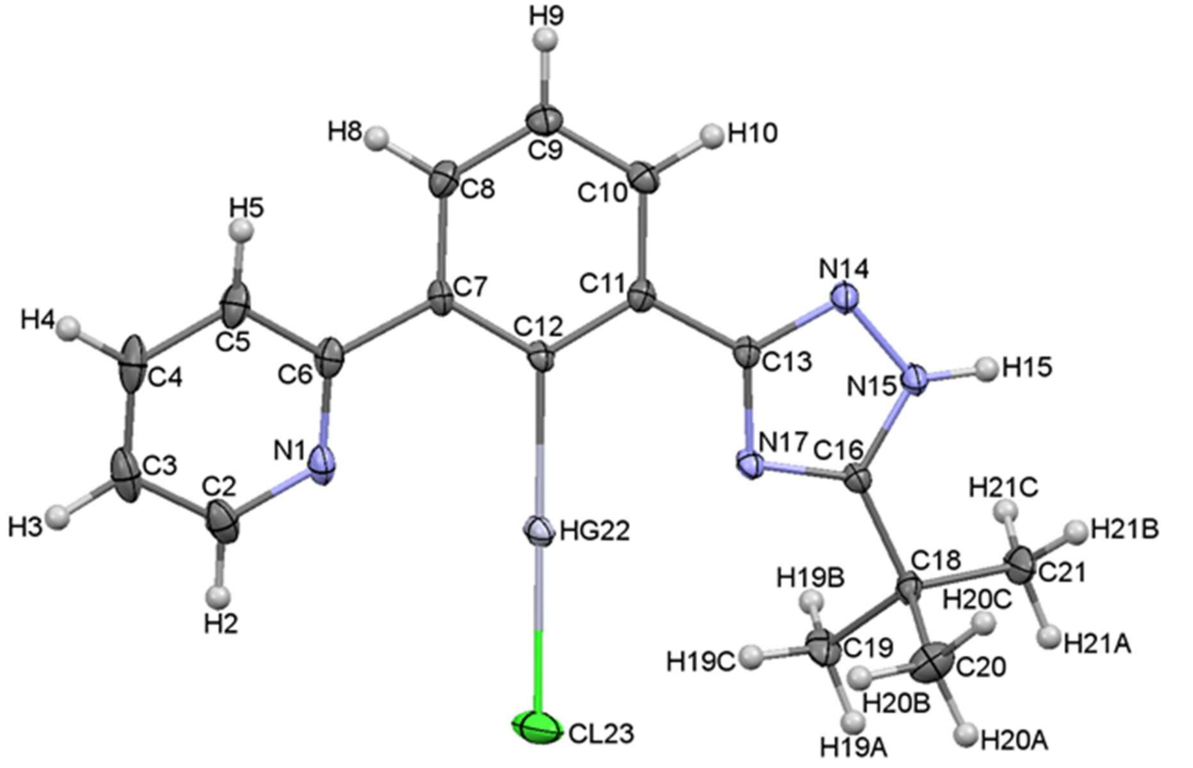

Figure S68. Molecular structure as ORTEP diagram (displacement ellipsoids are shown at 50% probability) of **[HLHgCl]**.

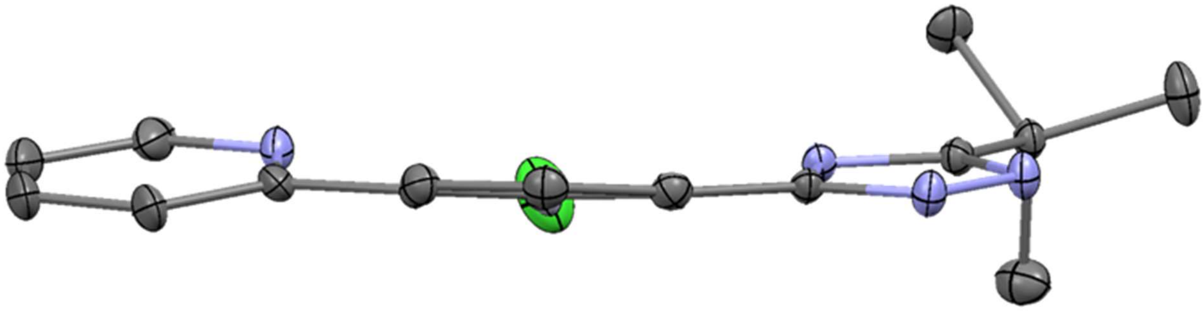

Figure S69. Molecular structure as ORTEP diagram (displacement ellipsoids are shown at 50% probability) of **[HLHgCl]**. View along the C-Hg-Cl axis, to depict the rotation of the triazole and pyridine (9.8° and 14.9°, respectively) unit out of the C-M-Cl coordination plane.

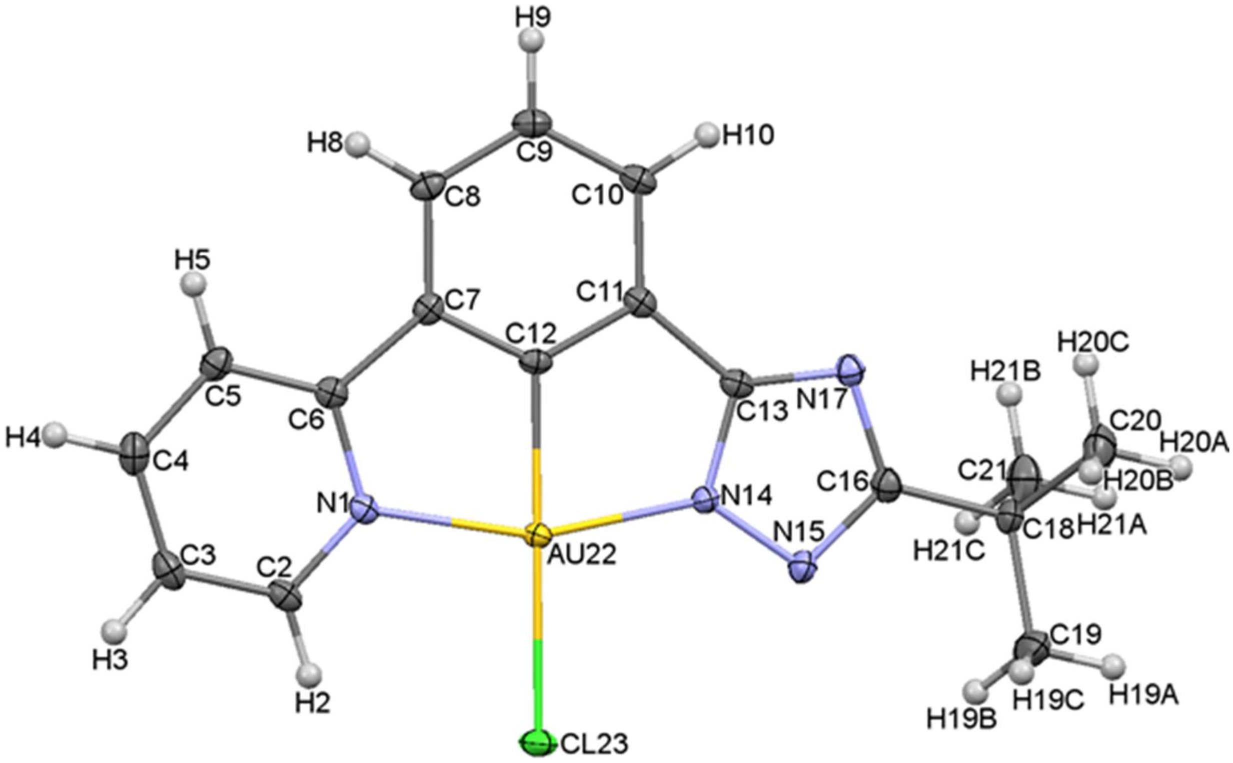

Figure S70. Molecular structure as ORTEP diagram (displacement ellipsoids are shown at 50% probability) of **[LAuCl]**.

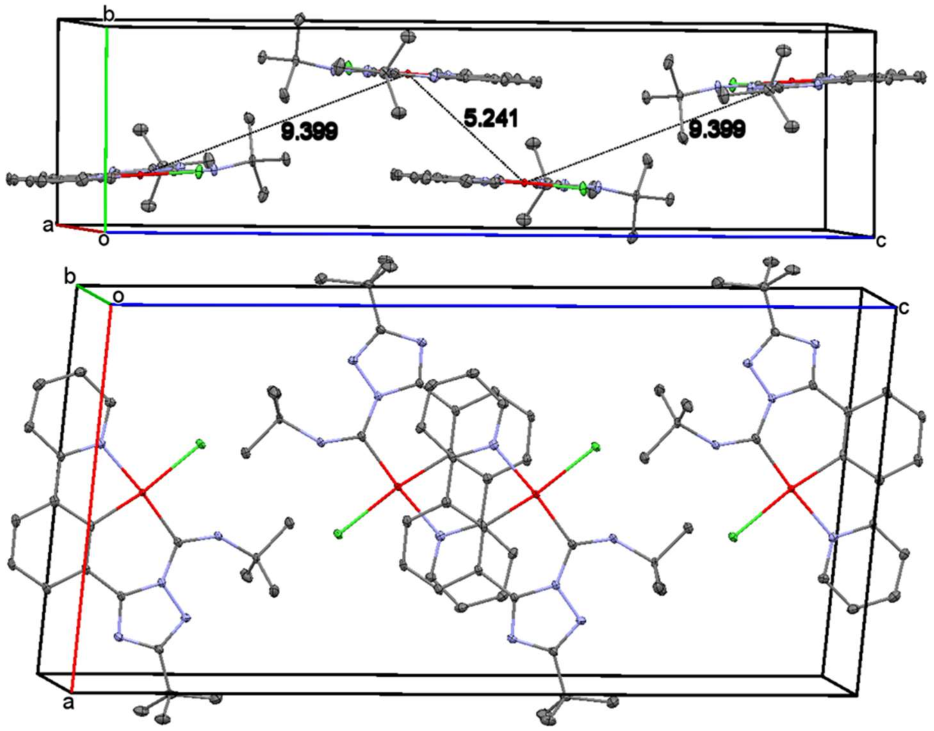

Figure S71. Crystal packing of **[LPtCN*t*Bu]**. Displacement ellipsoids are shown at 50% probability.

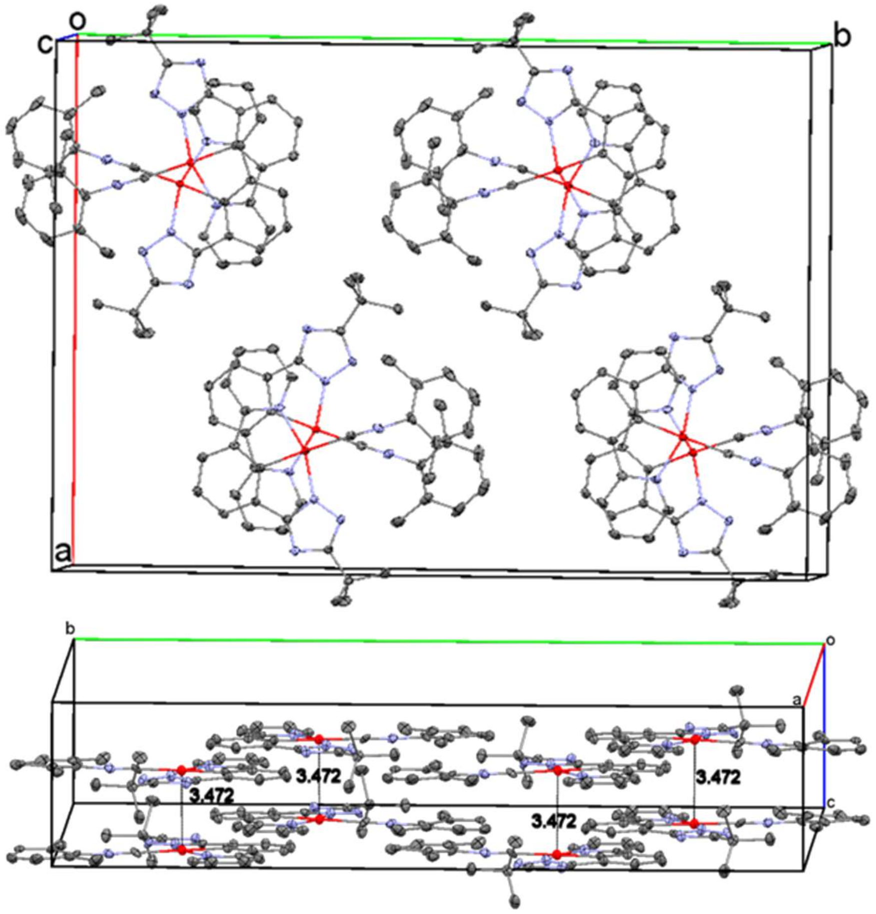

Figure S72. Crystal packing of **[LPtCNPhen]**. Displacement ellipsoids are shown at 50% probability.

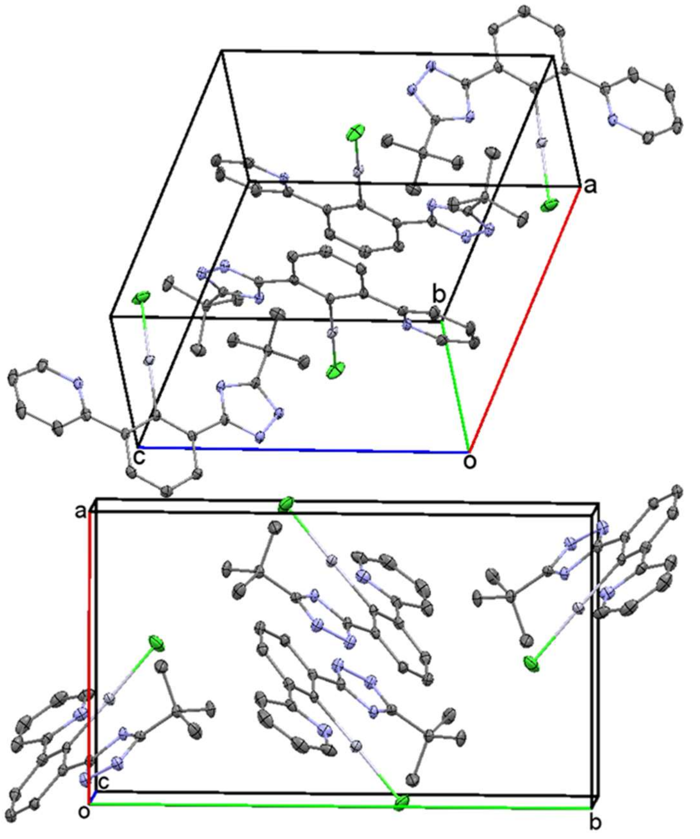

Figure S73. Crystal packing of **[HLHgCl]**. Displacement ellipsoids are shown at 50% probability.

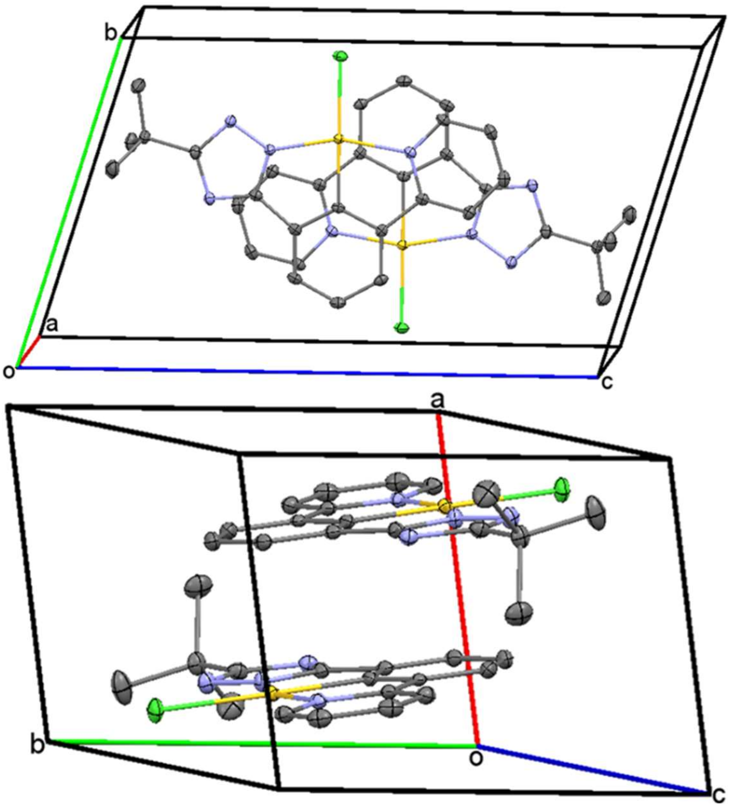

Figure S74. Crystal packaging of **[LAuCl]**. Displacement ellipsoids are shown at 50% probability.

Table S1. Overview of the crystallographic data for all the six compounds.

Complex	[LPtCN*t*Bu]	[LPtCNPhen]	[HLHgCl]	[LAuCl]
**Formula**	$C_{22}H_{26}ClN_5Pt$	$C_{26}H_{25}N_5Pt$	$C_{17}H_{17}ClHgN_4$	$C_{17}H_{16}AuClN_4$,
**MW**	591.02	602.59	513.39	586.89
**CCDC No.**	2284705	2284706	2284704	2284703
a **/ Å**	12.4332(4)	21.5351(7)	10.0279(3)	6.5585(3)
b **/ Å**	6.7495(2)	30.8227(10)	15.9330(4)	9.7929(5)
c **/ Å**	25.2633(7)	6.7839(2)	11.2101(3)	16.4484(7)
V **/ Å³**	2112.47(11)	4502.9(2)	1681.80(8)	997.07(8)
α **/ °**	90	90	90	73.520(1)
β **/ °**	94.843(1)	90	110.119(1)	88.097(2)
γ **/ °**	90	90	90	79.866(1)
**Space group**	$P2_1/c$	*Aba*2	$P2_1/n$	$P\bar{1}$
**Crystal system**	monoclinic	orthorhombic	monoclinic	triclinic
Z **value**	4	8	4	2
F_{000}	1152.0	2352.0	976.0	568.0
h, k, l_{max}	18, 10, 37	30, 43, 9	14, 23, 16	8, 12, 21
N_{ref}	7328	6570	5611	4680
T_{min}, T_{max}	0.296, 0.746	0.564, 0.746	0.010, 0.075	0.483, 0.746
**μ(MoKα) / mm−1**	6.787	6.256	9.314	7.633

Photophysical studies

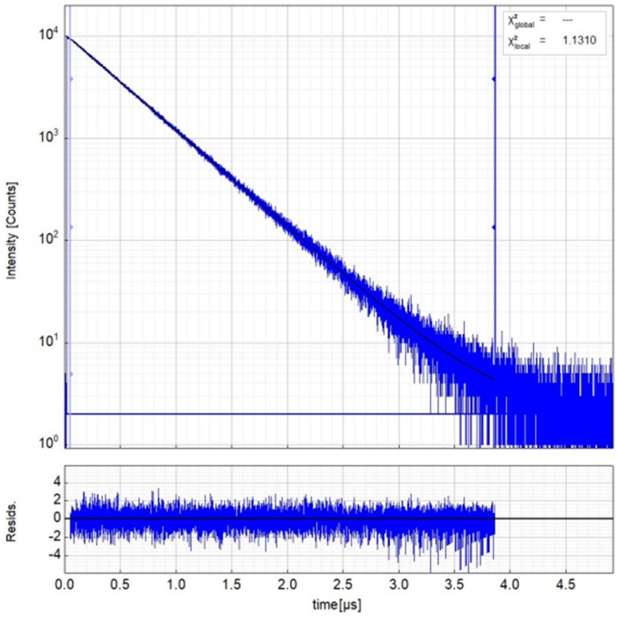

Parameter	Value	Δ	δ
A_1[kCnts/Chnl]	9.521	±0.012	0.1%
τ_1[ns]	458.83	±0.42	0.1%
I_1[kCnts]	8 736.5	±4.4	0.0%
A_{Rel1}[%]	100.0	---	---
I_{Rel1}[%]	100.0	---	---
$Bkgr_{Dec}$[kCnts]	0.0020	±0.0001	3.1%
τ_{AvInt}[ns]	458.83	±0.42	0.1%
τ_{AvAmp}[ns]	458.83	±0.42	0.1%

Figure S75. Left: Raw (experimental) time-resolved photoluminescence decay of **[LPtCN*t*Bu]** (10^{-5} M) in liquid DCM at 298 K (air-equilibrated, λ_{ex} = 375 nm, λ_{em} = 500 nm). Right: Fitting parameters including pre-exponential factors and confidence limits.

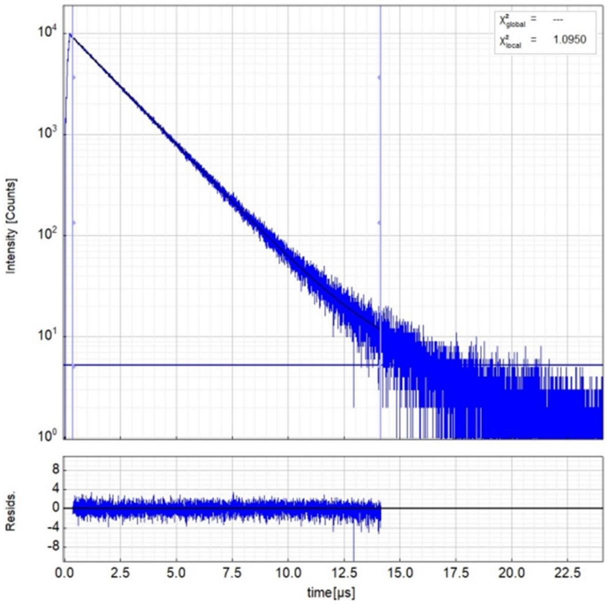

Parameter	Value	Δ	δ
A_1[kCnts/Chnl]	9.026	±0.012	0.1%
τ_1[ns]	1 904.3	±1.6	0.1%
I_1[kCnts]	8 593.0	±6.3	0.1%
A_{Rel1}[%]	100.0	---	---
I_{Rel1}[%]	100.0	---	---
$Bkgr_{Dec}$[kCnts]	0.0053	±0.0003	5.6%
τ_{AvInt}[ns]	1 904.3	±1.6	0.1%
τ_{AvAmp}[ns]	1 904.3	±1.6	0.1%

Figure S76. Left: Raw (experimental) time-resolved photoluminescence decay of **[LP*t*CNtBu]** (10^{-5} M) in liquid DCM at 298 K (Ar-purged, λ_{ex} = 375 nm, λ_{em} = 500 nm). Right: Fitting parameters including pre-exponential factors and confidence limits.

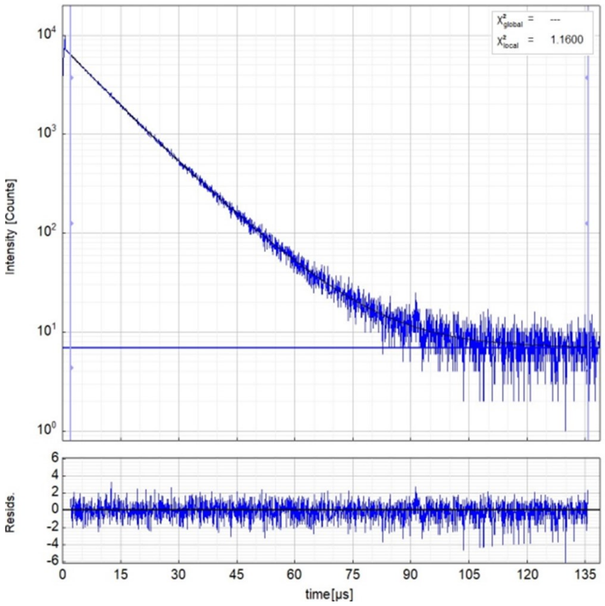

Parameter	Value	Δ	δ
A_1[kCnts/Chnl]	3.85	±0.20	5.1%
τ_1[ns]	9 440	±170	1.8%
I_1[kCnts]	568	±39	6.8%
A_{Rel1}[%]	61.1	±3.3	5.4%
I_{Rel1}[%]	51.7	±3.6	6.8%
A_2[kCnts/Chnl]	2.45	±0.22	8.7%
τ_2[ns]	13 850	±190	1.3%
I_2[kCnts]	530	±39	7.3%
A_{Rel2}[%]	39.0	±3.3	8.4%
I_{Rel2}[%]	48.4	±3.6	7.3%
$Bkgr_{Dec}$[kCnts]	0.0070	±0.0002	1.6%
τ_{AvInt}[ns]	11 566.6	±8.9	0.1%
τ_{AvAmp}[ns]	11 152	±20	0.2%

Figure S77. Left: Raw (experimental) time-resolved photoluminescence decay of **[LP*t*CNtBu]** (10^{-5} M) in a frozen glassy matrix (DCM-MeOH 1:1) at 77 K (λ_{ex} = 375 nm, λ_{em} = 500 nm). Right: Fitting parameters including pre-exponential factors and confidence limits.

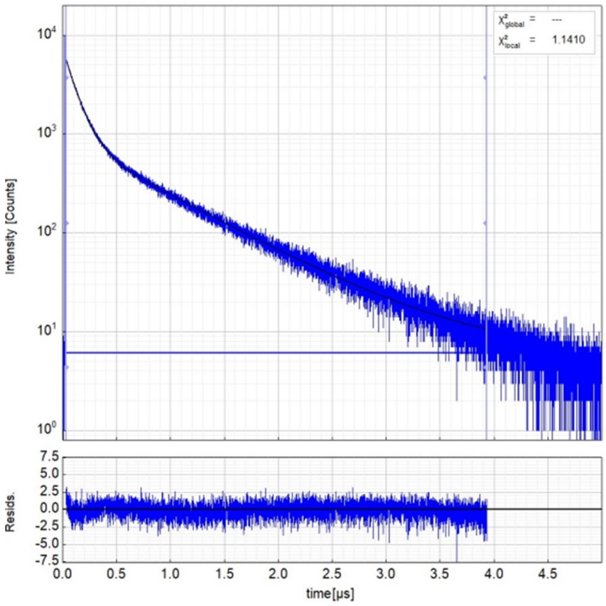

Parameter	Value	Δ	δ
A_1[kCnts/Chnl]	4.660	±0.033	0.7%
τ_1[ns]	109.48	±0.89	0.8%
I_1[kCnts]	1 020.3	±7.3	0.7%
A_{Rel1}[%]	84.2	±0.2	0.2%
I_{Rel1}[%]	44.1	±0.3	0.5%
A_2[kCnts/Chnl]	0.8749	±0.0067	0.8%
τ_2[ns]	741.8	±3.9	0.5%
I_2[kCnts]	1 298.0	±5.2	0.4%
A_{Rel2}[%]	15.9	±0.2	0.8%
I_{Rel2}[%]	56.0	±0.3	0.4%
$Bkgr_{Dec}$[kCnts]	0.0061	±0.0004	5.0%
τ_{AvInt}[ns]	463.6	±1.6	0.3%
τ_{AvAmp}[ns]	209.5	±1.2	0.5%

Figure S78. Left: Raw (experimental) time-resolved photoluminescence decay of **[LPtCNPhen]** (10^{-5} M) in liquid DCM at 298 K (air-equilibrated, λ_{ex} = 375 nm, λ_{em} = 515 nm). Right: Fitting parameters including pre-exponential factors and confidence limits.

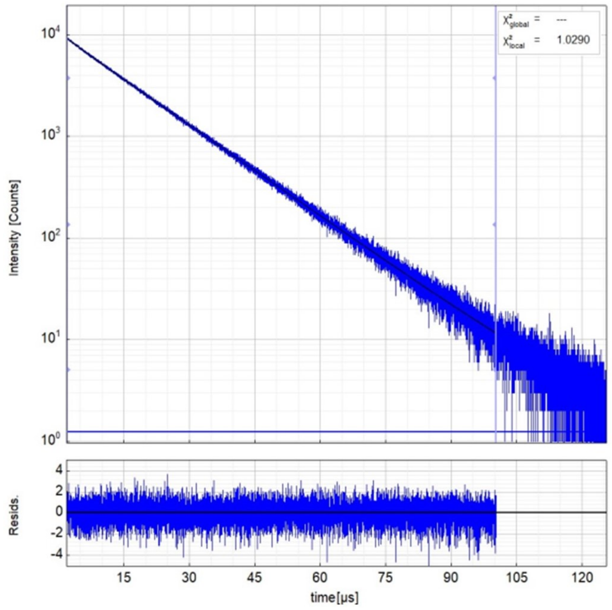

Parameter	Value	Δ	δ
A_1[kCnts/Chnl]	0.365	±0.037	9.9%
τ_1[ns]	4 070	±810	20%
I_1[kCnts]	185	±54	29%
A_{Rel1}[%]	4.0	±0.5	10%
I_{Rel1}[%]	1.2	±0.4	29%
A_2[kCnts/Chnl]	8.820	±0.047	0.5%
τ_2[ns]	14 565	±33	0.2%
I_2[kCnts]	16 057	±51	0.3%
A_{Rel2}[%]	96.1	±0.5	0.4%
I_{Rel2}[%]	98.9	±0.4	0.3%
$Bkgr_{Dec}$[kCnts]	0.0013	±0.0003	23%
τ_{AvInt}[ns]	14 445	±12	0.1%
τ_{AvAmp}[ns]	14 148	±36	0.3%

Figure S79. Left: Raw (experimental) time-resolved photoluminescence decay of **[LPtCNPhen]** (10^{-5} M) in liquid DCM at 298 K (Ar-purged, λ_{ex} = 375 nm, λ_{em} = 515 nm). Right: Fitting parameters including pre-exponential factors and confidence limits.

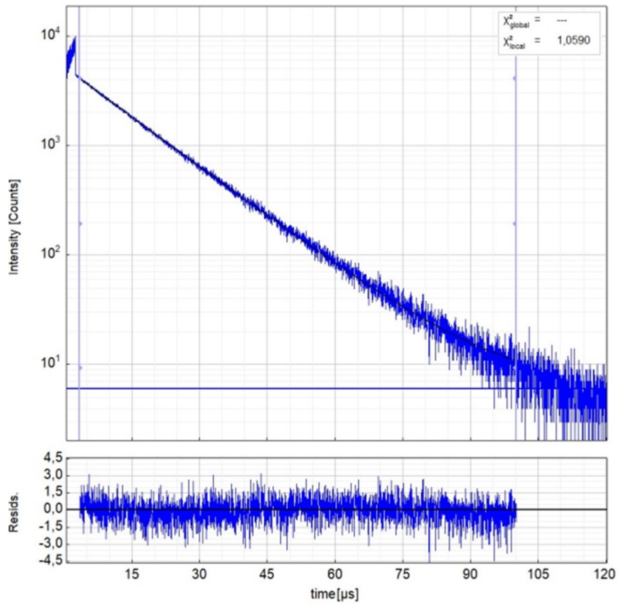

Parameter	Value	Δ	δ
A_1[kCnts/Chnl]	4,0447	±0,0069	0,2%
τ_1[ns]	14 367	±22	0,2%
I_1[kCnts]	1 815,9	±2,1	0,1%
A_{Rel1}[%]	100,0	---	---
I_{Rel1}[%]	100,0	---	---
$Bkgr_{Dec}$[kCnts]	0,0061	±0,0003	4,9%
τ_{AvInt}[ns]	14 367	±22	0,2%
τ_{AvAmp}[ns]	14 367	±22	0,2%

Figure S80. Left: Raw (experimental) time-resolved photoluminescence decay of **[LPtCNPhen]** (10^{-5} M) in a frozen glassy matrix (DCM-MeOH 1:1) at 77 K (λ_{ex} = 375 nm, λ_{em} = 470 nm). Right: Fitting parameters including pre-exponential factors and confidence limits.

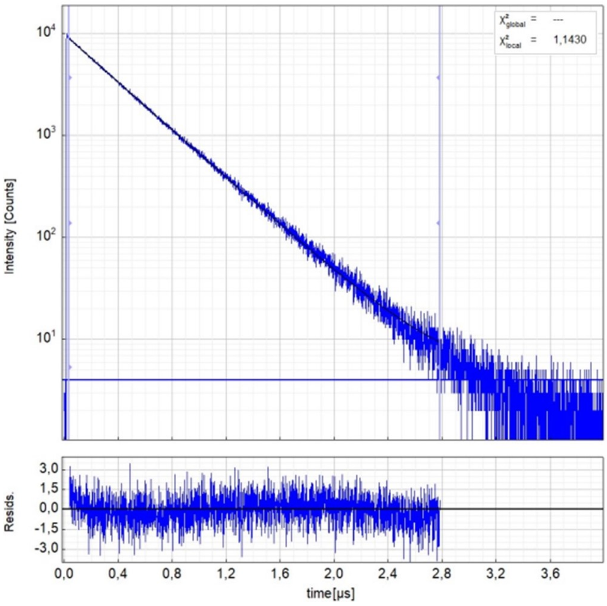

Parameter	Value	Δ	δ
A_1[kCnts/Chnl]	8,892	±0,016	0,2%
τ_1[ns]	370,57	±0,52	0,1%
I_1[kCnts]	3 294,9	±3,1	0,1%
A_{Rel1}[%]	100,0	---	---
I_{Rel1}[%]	100,0	---	---
$Bkgr_{Dec}$[kCnts]	0,0040	±0,0004	8,9%
τ_{AvInt}[ns]	370,57	±0,52	0,1%
τ_{AvAmp}[ns]	370,57	±0,52	0,1%

Figure S81. Left: Raw (experimental) time-resolved photoluminescence decay of **[LPtCl]** (10^{-5} M) in liquid DCM at 298 K (air-equilibrated, λ_{ex} = 375 nm, λ_{em} = 480 nm). Right: Fitting parameters including pre-exponential factors and confidence limits.

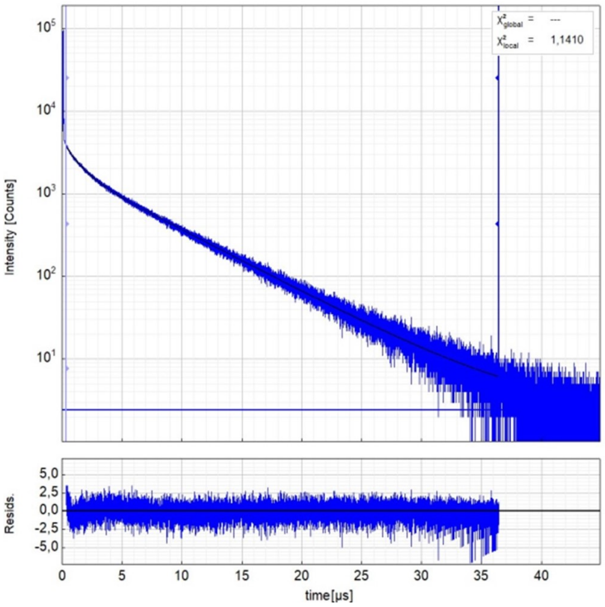

Parameter	Value	Δ	δ
A_1[kCnts/Chnl]	1,783	±0,013	0,7%
τ_1[ns]	1 158	±16	1,4%
I_1[kCnts]	1 032	±14	1,3%
A_{Rel1}[%]	47,7	±0,3	0,5%
I_{Rel1}[%]	15,5	±0,3	1,3%
A_2[kCnts/Chnl]	1,9593	±0,0089	0,5%
τ_2[ns]	5 764	±14	0,2%
I_2[kCnts]	5 647	±14	0,2%
A_{Rel2}[%]	52,4	±0,3	0,4%
I_{Rel2}[%]	84,6	±0,3	0,2%
$Bkgr_{Dec}$[kCnts]	0,0025	±0,0002	5,7%
τ_{AvInt}[ns]	5 052,1	±7,1	0,1%
τ_{AvAmp}[ns]	3 570	±14	0,4%

Figure S82. Left: Raw (experimental) time-resolved photoluminescence decay of **[LPtCl]** (10^{-5} M) in liquid DCM at 298 K (Ar-purged, λ_{ex} = 375 nm, λ_{em} = 480 nm). Right: Fitting parameters including pre-exponential factors and confidence limits.

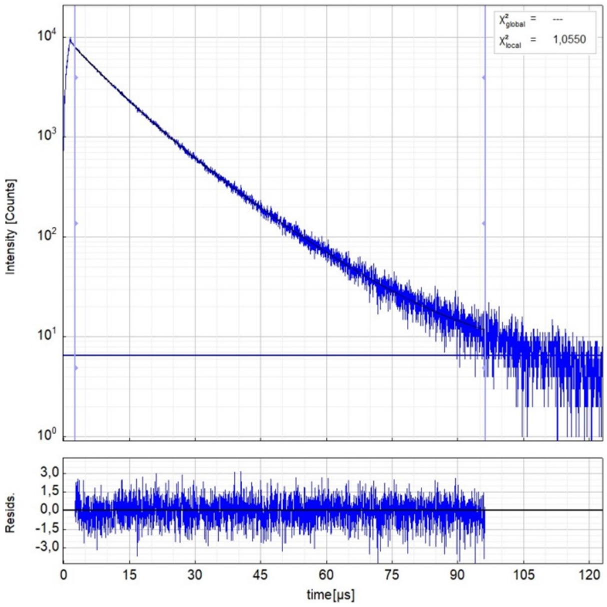

Parameter	Value	Δ	δ
A_1[kCnts/Chnl]	2,90	±0,27	9,1%
τ_1[ns]	14 820	±370	2,4%
I_1[kCnts]	1 342	±87	6,4%
A_{Rel1}[%]	36,7	±3,3	9,0%
I_{Rel1}[%]	52,0	±3,5	6,5%
A_2[kCnts/Chnl]	5,01	±0,26	5,1%
τ_2[ns]	7 930	±180	2,2%
I_2[kCnts]	1 240	±90	7,2%
A_{Rel2}[%]	63,4	±3,3	5,2%
I_{Rel2}[%]	48,1	±3,5	7,1%
$Bkgr_{Dec}$[kCnts]	0,0066	±0,0008	11%
τ_{AvInt}[ns]	11 506	±46	0,4%
τ_{AvAmp}[ns]	10 449	±36	0,3%

Figure S83. Left: Raw (experimental) time-resolved photoluminescence decay of **[LPtCl]** (10^{-5} M) in a frozen glassy matrix (DCM-MeOH 1:1) at 77 K (λ_{ex} = 375 nm, λ_{em} = 525 nm). Right: Fitting parameters including pre-exponential factors and confidence limits.

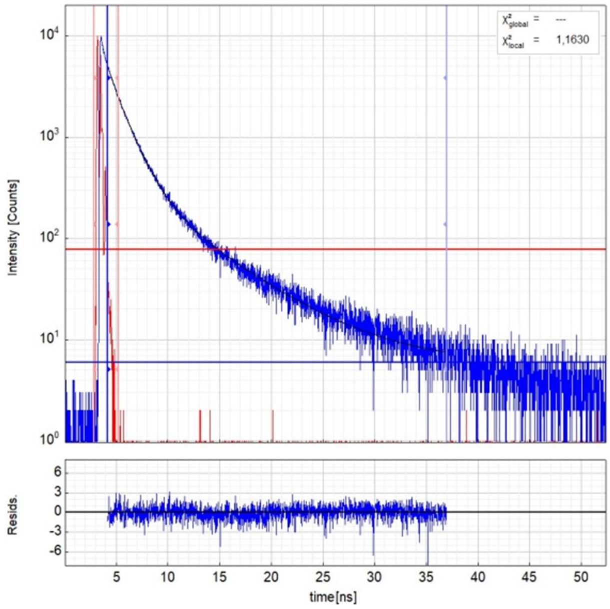

Parameter	Value	Δ	δ
A_1[kCnts/Chnl]	8,830	±0,099	1,1%
τ_1[ns]	1,5041	±0,0024	0,2%
I_1[kCnts]	830,1	±9,0	1,1%
A_{Rel1}[%]	94,2	±0,2	0,2%
I_{Rel1}[%]	80,3	±0,5	0,6%
A_2[kCnts/Chnl]	0,553	±0,014	2,5%
τ_2[ns]	5,910	±0,030	0,5%
I_2[kCnts]	204,1	±4,7	2,3%
A_{Rel2}[%]	5,9	±0,2	3,0%
I_{Rel2}[%]	19,8	±0,5	2,4%
$Bkgr_{Dec}$[kCnts]	0,0060	±0,0007	11%
$Bkgr_{IRF}$[Cnts/Chnl]	76,91	±0,77	1,0%
$Shift_{IRF}$[ps]	- 247,992	±0,025	0,0%
A_{Scatt}[kCnts]	64	±66	102%
τ_{AvInt}[ns]	2,374	±0,019	0,8%
τ_{AvAmp}[ns]	1,7636	±0,0072	0,4%

Figure S84. Left: Raw (experimental) time-resolved photoluminescence decay of **[HLHgCl]** (10^{-5} M) in liquid MeOH at 298 K (air-equilibrated, λ_{ex} = 375 nm, λ_{em} = 440 nm) and the instrument response function (red), including the residuals (λ_{ex} = 376.7 nm, λ_{em} = 550 nm). Right: Fitting parameters including pre-exponential factors and confidence limits.

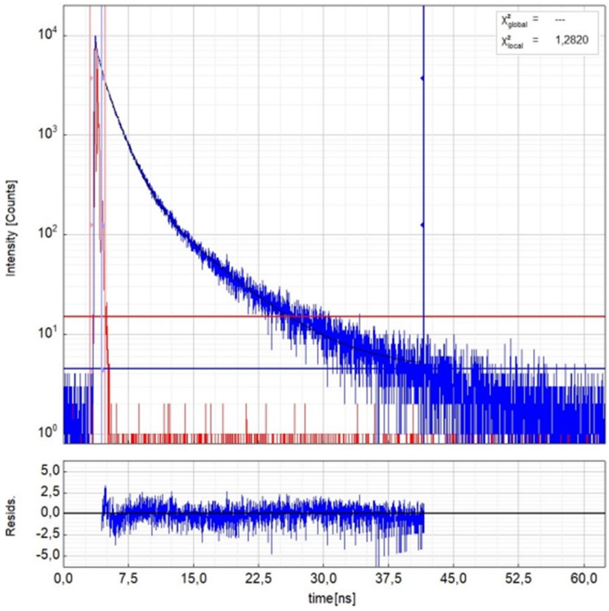

Parameter	Value	Δ	δ
A_1[kCnts/Chnl]	0,523	±0,037	7,0%
τ_1[ns]	5,74	±0,18	3,1%
I_1[kCnts]	187,4	±7,6	4,0%
A_{Rel1}[%]	8,5	±0,6	6,0%
I_{Rel1}[%]	25,2	±1,0	3,9%
A_2[kCnts/Chnl]	5,67	±0,21	3,7%
τ_2[ns]	1,574	±0,027	1,7%
I_2[kCnts]	557	±20	3,5%
A_{Rel2}[%]	91,6	±0,6	0,6%
I_{Rel2}[%]	74,9	±1,0	1,3%
$Bkgr_{Dec}$[kCnts]	0,0045	±0,0004	8,5%
$Bkgr_{IRF}$[Cnts/Chnl]	15,0000	---	---
$Shift_{IRF}$[ps]	60	±54	89%
A_{Scatt}[kCnts]	230	±120	49%
τ_{AvInt}[ns]	2,621	±0,035	1,3%
τ_{AvAmp}[ns]	1,926	±0,023	1,2%

Figure S85. Left: Raw (experimental) time-resolved photoluminescence decay of **[HLHgCl]** (10^{-5} M) in liquid MeOH at 298 K (Ar-purged, λ_{ex} = 375 nm, λ_{em} = 480 nm) and the instrument response function (red), including the residuals (λ_{ex} = 376.7 nm, λ_{em} = 550 nm). Right: Fitting parameters including pre-exponential factors and confidence limits.

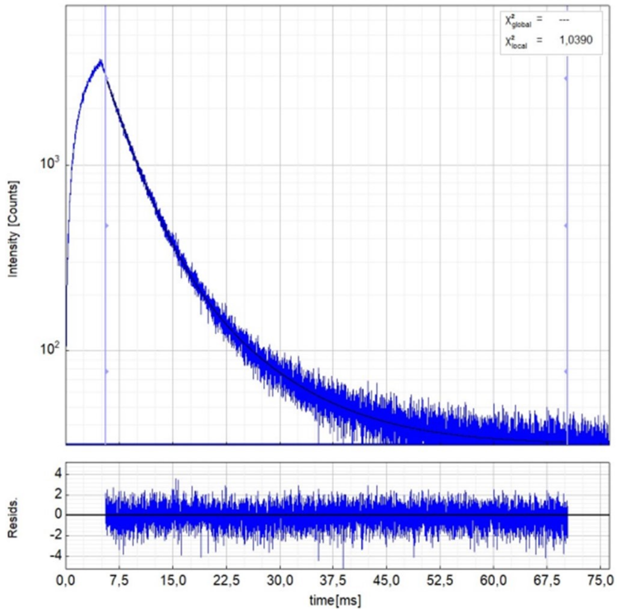

Parameter	Value	Δ	δ
A_1[kCnts/Chnl]	0,469	±0,028	5,9%
τ_1[ns]	10 230 000	±290 000	2,8%
I_1[kCnts]	586	±20	3,3%
A_{Rel1}[%]	16,0	±1,0	5,7%
I_{Rel1}[%]	36,3	±1,3	3,3%
A_2[kCnts/Chnl]	2,465	±0,025	1,0%
τ_2[ns]	3 425 000	±43 000	1,2%
I_2[kCnts]	1 031	±21	2,0%
A_{Rel2}[%]	84,1	±1,0	1,1%
I_{Rel2}[%]	63,8	±1,3	1,9%
$Bkgr_{Dec}$[kCnts]	0,0316	±0,0004	1,2%
τ_{AvInt}[ns]	5 887 000	±50 000	0,8%
τ_{AvAmp}[ns]	4 512 000	±19 000	0,4%

Figure S86. Left: Raw (experimental) time-resolved photoluminescence decay of **[HLHgCl]** (10^{-5} M) in a frozen glassy matrix (DCM-MeOH 1:1) at 77 K (λ_{ex} = 317 nm, λ_{em} = 500 nm). Right: Fitting parameters including pre-exponential factors and confidence limits.

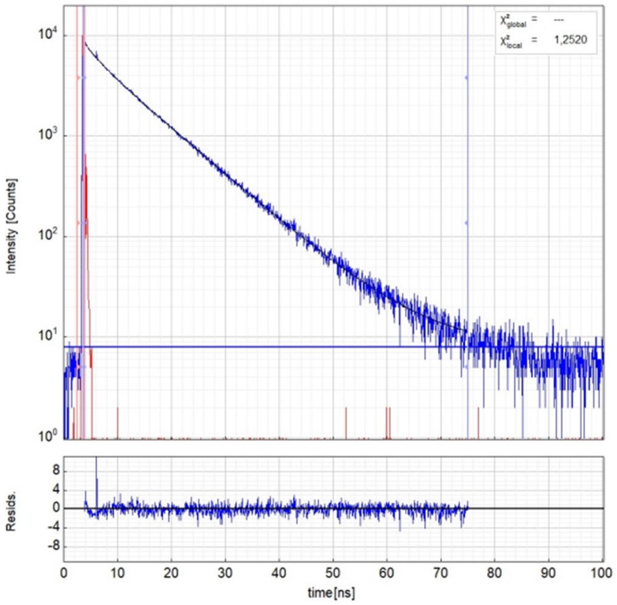

Parameter	Value	Δ	δ
A_1[kCnts/Chnl]	5,79	±0,14	2,3%
τ_1[ns]	9,430	±0,068	0,7%
I_1[kCnts]	853	±19	2,1%
A_{Rel1}[%]	77,2	±1,1	1,4%
I_{Rel1}[%]	92,6	±1,1	1,1%
A_2[kCnts/Chnl]	1,72	±0,12	6,8%
τ_2[ns]	2,55	±0,33	13%
I_2[kCnts]	69	±11	15%
A_{Rel2}[%]	22,9	±1,1	4,8%
I_{Rel2}[%]	7,5	±1,1	14%
$Bkgr_{Dec}$[kCnts]	0,0080	±0,0006	7,5%
$Bkgr_{IRF}$[Cnts/Chnl]	- 316	±32	10%
$Shift_{IRF}$[ps]	10	±13	129%
A_{Scatt}[kCnts]	13,4	±2,4	18%
τ_{AvInt}[ns]	8,920	±0,029	0,3%
τ_{AvAmp}[ns]	7,86	±0,12	1,5%

Figure S87. Left: Raw (experimental) time-resolved photoluminescence decay of **[HLPdCN*t*Bu]** (10^{-5} M) in liquid MeOH at 298 K (air-equilibrated, λ_{ex} = 375 nm, λ_{em} = 450 nm) and the instrument response function (red), including the residuals (λ_{ex} = 376.7 nm, λ_{em} = 550 nm). Right: Fitting parameters including pre-exponential factors and confidence limits.

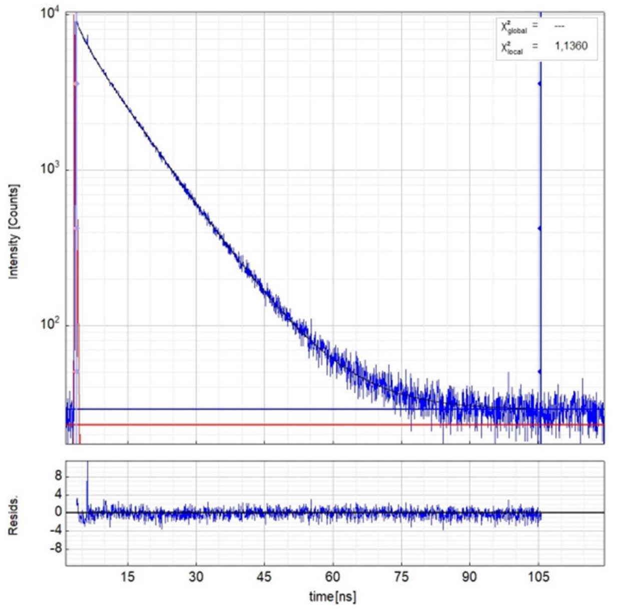

Parameter	Value	Δ	δ
A_1[kCnts/Chnl]	8,13	±0,12	1,5%
τ_1[ns]	10,336	±0,075	0,7%
I_1[kCnts]	1 313	±20	1,5%
A_{Rel1}[%]	79,0	±1,4	1,7%
I_{Rel1}[%]	94,8	±1,0	1,0%
A_2[kCnts/Chnl]	2,17	±0,18	8,0%
τ_2[ns]	2,14	±0,48	22%
I_2[kCnts]	73	±15	20%
A_{Rel2}[%]	21,1	±1,4	6,5%
I_{Rel2}[%]	5,3	±1,0	18%
$Bkgr_{Dec}$[kCnts]	0,0289	±0,0006	1,8%
$Bkgr_{IRF}$[Cnts/Chnl]	23	±13	53%
$Shift_{IRF}$[ps]	- 193	±33	17%
τ_{AvInt}[ns]	9,908	±0,034	0,3%
τ_{AvAmp}[ns]	8,62	±0,22	2,5%

Figure S88. Left: Raw (experimental) time-resolved photoluminescence decay of **[HLPdCN*t*Bu]** (10^{-5} M) in liquid MeOH at 298 K (Ar-purged, λ_{ex} = 375 nm, λ_{em} = 450 nm) and the instrument response function (red), including the residuals (λ_{ex} = 376.7 nm, λ_{em} = 550 nm). Right: Fitting parameters including pre-exponential factors and confidence limits.

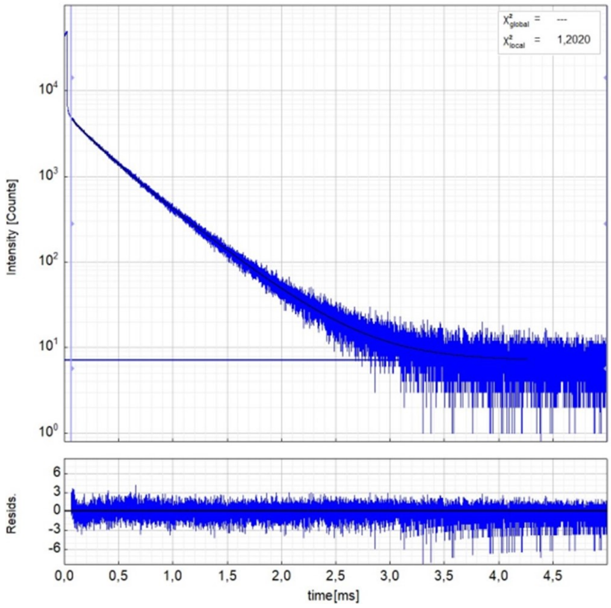

Parameter	Value	Δ	δ
A_1[kCnts/Chnl]	3,285	±0,079	2,4%
τ_1[ns]	445 300	±3 000	0,7%
I_1[kCnts]	5 712	±98	1,7%
A_{Rel1}[%]	69,3	±1,5	2,2%
I_{Rel1}[%]	83,5	±1,5	1,7%
A_2[kCnts/Chnl]	1,456	±0,069	4,7%
τ_2[ns]	199 500	±8 200	4,1%
I_2[kCnts]	1 134	±99	8,7%
A_{Rel2}[%]	30,8	±1,5	4,9%
I_{Rel2}[%]	16,6	±1,5	8,7%
$Bkgr_{Dec}$[kCnts]	0,0071	±0,0001	1,0%
τ_{AvInt}[ns]	404 530	±460	0,1%
τ_{AvAmp}[ns]	369 800	±1 200	0,3%

Figure S89. Left: Raw (experimental) time-resolved photoluminescence decay of **[HLPdCN*t*Bu]** (10^{-5} M) in a frozen glassy matrix (DCM-MeOH 1:1) at 77 K (λ_{ex} = 317 nm, λ_{em} = 500 nm). Right: Fitting parameters including pre-exponential factors and confidence limits.

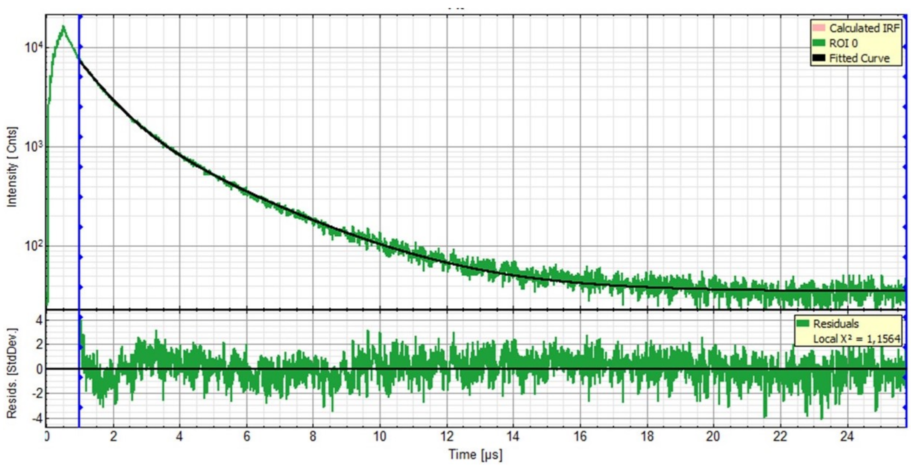

Parameter		Data Set (3)
t_0[ns]		002,0000 ± ------
A_1 [kCnts]	Limits	1,980 ± 0,044
A_2 [kCnts]	Limits	5,280 ± 0,026
τ_1 [ns]	Limits	2686 ± 18
τ_2 [ns]	Limits	821 ± 12
$Bkgr_{Dec}$[Cnts]	Limits	36,200 ± 0,062
I_1 [kCnts]		520,0 ± 8,2
I_2 [kCnts]		424,0 ± 8,4
I_{Sum} [kCnts]		943,0 ± 1,5
A_{Sum} [kCnts]		7,30 ± 0,03
$\tau_{Av Int}$[ns]		1849 ± 3
$\tau_{Av Amp}$[ns]		1330,0 ± 3,9
$A_{rel 1}$		0,2728 ± 0,0059
$A_{rel 2}$		0,7272 ± 0,0059

Figure S90. Left: Raw (experimental) time-resolved photoluminescence decay of **[LPtCN*t*Bu]** as a crystal at 298 K using a one-photon laser as the excitation source (λ_{ex} = 375 nm). Right: Fitting parameters including pre-exponential factors and confidence limits.

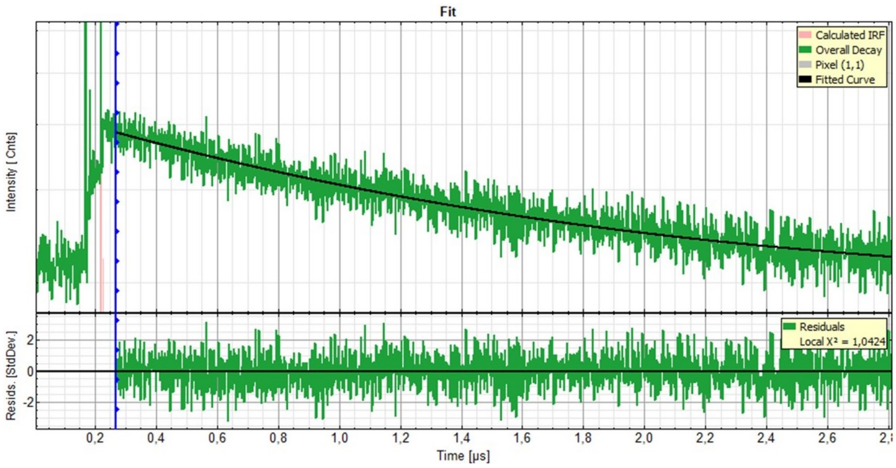

Parameter		Overall Decay
t_0[ns]		267,4000 ± ------
A_1 [kCnts]	Limits	0,1917 ± 0,0036
τ_1 [ns]	Limits	1368 ± 72
$Bkgr_{Dec}$[Cnts]	Limits	195,0 ± 4,9
I_1 [kCnts]		205 ± 14
I_{Sum} [kCnts]		205 ± 14
A_{Sum} [kCnts]		0,1917 ± 0,0036
$\tau_{Av Int}$[ns]		1368 ± 72
$\tau_{Av Amp}$[ns]		1368 ± 72
$A_{rel 1}$		1,000 ± 0,000

Figure S91. Left: Raw (experimental) time-resolved photoluminescence decay of **[LPtCN*t*Bu]** as a crystal at 298 K using a two-photon laser as the excitation source (λ_{ex} = 810 nm). Right: Fitting parameters including pre-exponential factors and confidence limits.

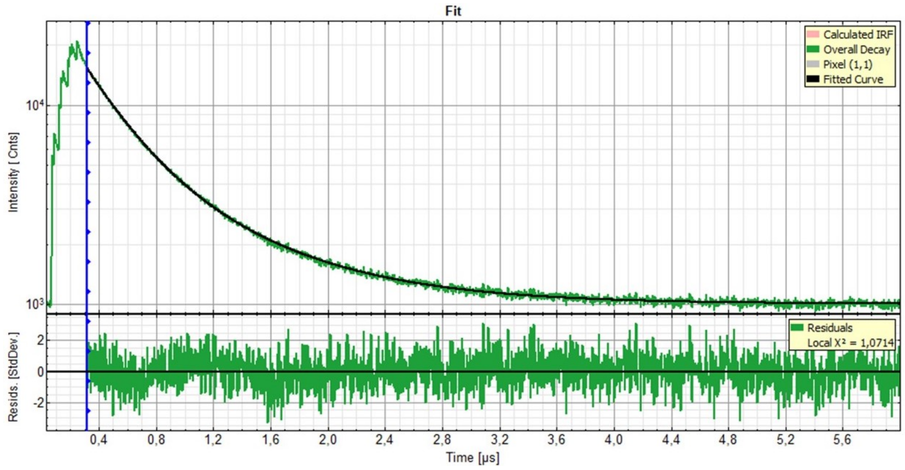

Parameter	Overall Decay
t_0[ns]	316,1000 ± ------
A_1[kCnts]	4,80 ± 0,22
A_2[kCnts]	9,50 ± 0,19
τ_1[ns]	797 ± 17
τ_2[ns]	296,0 ± 4,8
$Bkgr_{Dec}$[Cnts]	1006,0 ± 2,5
I_1[kCnts]	1499 ± 38
I_2[kCnts]	1099 ± 40
I_{Sum}[kCnts]	2598,0 ± 3,8
A_{Sum}[kCnts]	14,300 ± 0,012
τ_{AvInt}[ns]	585,0 ± 3,3
τ_{AvAmp}[ns]	464,0 ± 1,1
A_{rel1}	0,336 ± 0,015
A_{rel2}	0,664 ± 0,015

Figure S92. Left: Raw (experimental) time-resolved photoluminescence decay of **[LPtCNPhen]** as a crystal at 298 K using a one-photon laser as the excitation source (λ_{ex} = 375 nm). Right: Fitting parameters including pre-exponential factors and confidence limits.

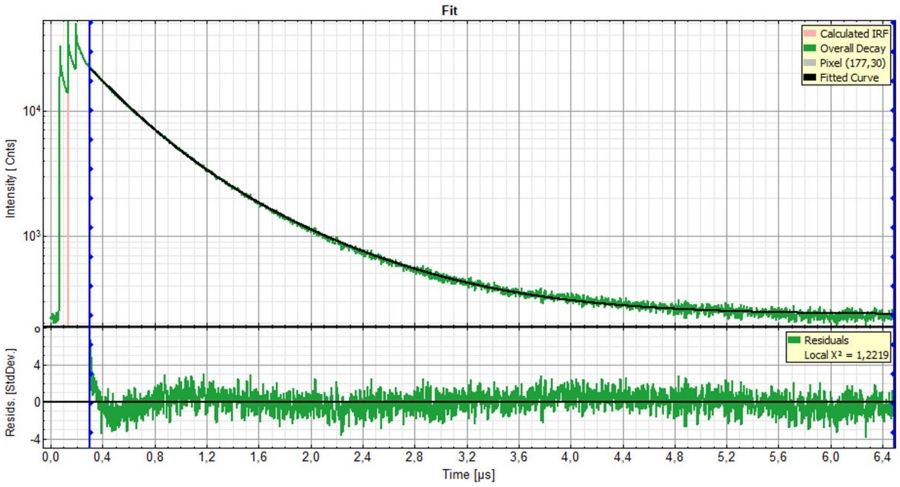

Parameter	Overall Decay
t_0[ns]	298,3000 ± ------
A_1[kCnts]	6,4 ± 0,2
A_2[kCnts]	15,00 ± 0,12
τ_1[ns]	819,0 ± 9,1
τ_2[ns]	330,0 ± 3,9
$Bkgr_{Dec}$[Cnts]	234,00 ± 0,42
I_1[kCnts]	2047 ± 41
I_2[kCnts]	1977 ± 41
I_{Sum}[kCnts]	4025,0 ± 2,2
A_{Sum}[kCnts]	21,700 ± 0,066
τ_{AvInt}[ns]	579,0 ± 1,7
τ_{AvAmp}[ns]	474,0 ± 1,3
A_{rel1}	0,2945 ± 0,0081
A_{rel2}	0,7055 ± 0,0081

Figure S93. Left: Raw (experimental) time-resolved photoluminescence decay of **[LPtCNPhen]** as a crystal at 298 K using a two-photon laser as the excitation source (λ_{ex} = 810 nm). Right: Fitting parameters including pre-exponential factors and confidence limits.

References

[1] R. K. Harris, E.D. Becker, S. M. Cabral De Menezes, R. Goodfellow, P. Granger, *Pure Appl. Chem.* **2001**, *73*, 1795–1818.
